# Biogreen Synthesis of Carbon Dots for Biotechnology and Nanomedicine Applications

**DOI:** 10.1007/s40820-018-0223-3

**Published:** 2018-10-17

**Authors:** Kok Ken Chan, Stephanie Hui Kit Yap, Ken-Tye Yong

**Affiliations:** School of Electrical and Electronic Engineering, Nanyang Technological University, Singapore, 639798 Singapore

**Keywords:** Carbon dots, Heavy metal sensing, Photoluminescence mechanism, Sensing mechanism, Sensor design

## Abstract

Over the past decade, carbon dots have ignited a burst of interest in many different fields, including nanomedicine, solar energy, optoelectronics, energy storage, and sensing applications, owing to their excellent photoluminescence properties and the easiness to modify their optical properties through doping and functionalization. In this review, the synthesis, structural and optical properties, as well as photoluminescence mechanisms of carbon dots are first reviewed and summarized. Then, we describe a series of designs for carbon dot-based sensors and the different sensing mechanisms associated with them. Thereafter, we elaborate on recent research advances on carbon dot-based sensors for the selective and sensitive detection of a wide range of analytes, including heavy metals, cations, anions, biomolecules, biomarkers, nitroaromatic explosives, pollutants, vitamins, and drugs. Lastly, we provide a concluding perspective on the overall status, challenges, and future directions for the use of carbon dots in real-life sensing.
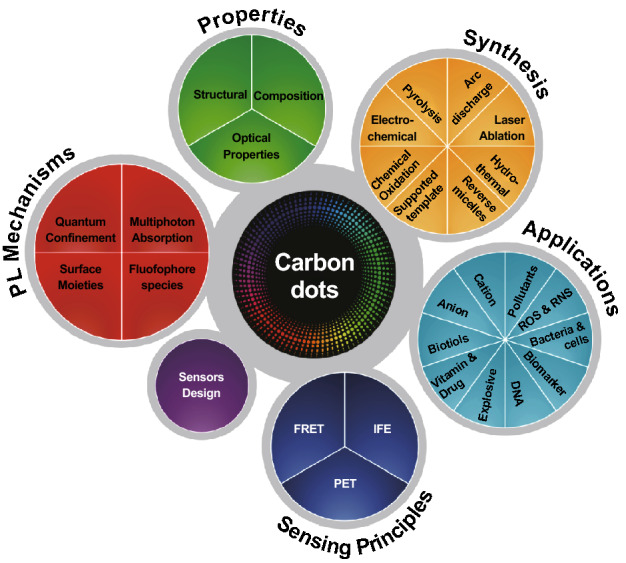

## Highlights

- Introduction to carbon dots: properties, photoluminescence mechanism, and synthesis.
- The design principles of carbon dot-based sensors and sensing mechanisms.
- The versatility of carbon dots for the sensing of different analytes.

## Introduction

The detection of chemical and biological agents is of great concern not only among the research community and environmentalists but among the general population, who are now more informed about the grave consequences associated with them. Although some of these agents may play vital roles in our daily lives, excessive exposure can be toxic and detrimental to the well-being of humans and the environment. For instance, metal ions such as iron and manganese play significant roles in many vital biological events such as oxygen transportation, oxidative phosphorylation, myelin production and synthesis, and metabolism of neurotransmitters [1]. However, high concentrations of iron in the body are known to be carcinogenic, presumably due to the generation of reactive oxygen species [2–4]. On the other hand, excessive exposure to manganese has been linked to neurobehavioral disorder in both adults and children [5]. It is well known that heavy metal ions, e.g., lead, mercury, cadmium, and arsenic, are among the most toxic ions to the human body without any known beneficial effects. Continuous exposure and accumulation over time will lead to serious debilitating diseases. Hence, the development of heavy metal ion detection methods with high selectivity and sensitivity has been receiving increasing attention from the research community.

Atomic absorption spectroscopy, inductively coupled plasma atomic emission spectroscopy, and electrochemical sensing are the conventional methods to detect heavy metal ions in minute quantity [6–11]. However, these methods require expensive equipment and often involve time-consuming analytical procedures that can only be carried out by trained personnel in laboratories. On the other hand, fluorescence-based sensing is gaining popularity due to its virtues of excellent sensitivity, fast response, simple fabrication procedure or straightforward sensing, and low development cost. This sensing method involves various types of fluorescent materials, such as organic dyes, fluorescent proteins, metal–organic frameworks, and quantum dots (QDs), which are often associated with low photostability, blinking effects, and may also pose toxicity concerns. For instance, group II–VI semiconductor QDs such as cadmium sulfide and cadmium telluride, which have been previously employed for biological sensing purposes, can be viewed as a legitimate toxicity concern as these QDs may potentially degrade and release toxic cadmium ions intracellularly [12]. This has led to the exploration of new materials with negligible toxicity and excellent physical and optical properties. Over the past decade, carbon nanomaterials have been widely investigated for their diverse applications, from electronics and solar cells to biological and sensing applications. Carbon nanomaterials can be prepared with various dimensions, from two-dimensional graphene and graphene nanoribbons, to one-dimensional carbon nanotubes (CNTs) and nanohorns, to zero-dimensional nanodiamonds, graphene quantum dots, and carbon dots (CDs) [13]. These carbon nanomaterials present vast differences in the spatial arrangement of carbon atoms, which ultimately leads to their distinct properties.

**Fig. 1:**
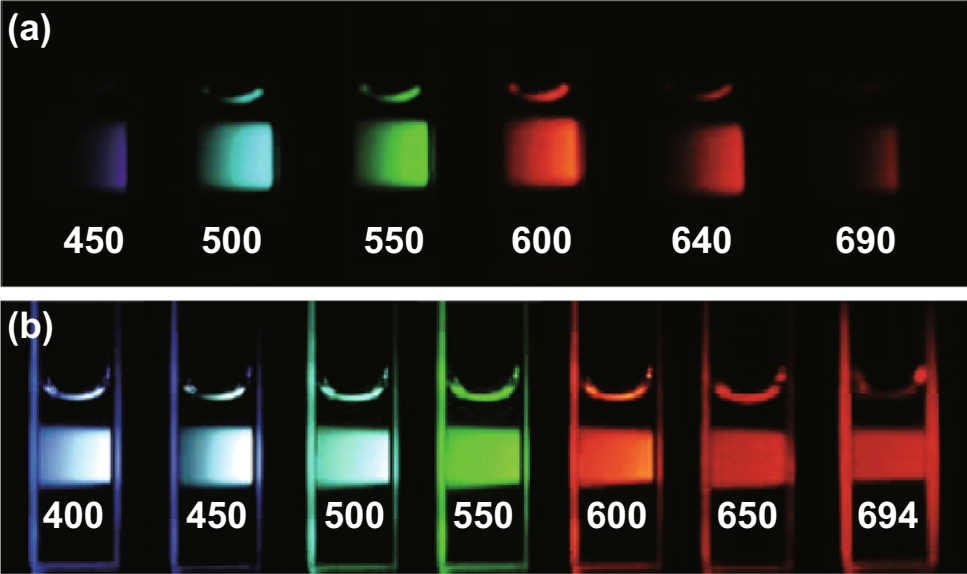
Aqueous solution of passivated CDs. **a** Excited at 400 nm and imaged through bandpass filters as indicated, and **b** excited at the indicated wavelength [15]. Copyright © 2006 American Chemical Society

CDs are the latest addition to the carbon family, which were serendipitously discovered by Xu et al. [14] during gel electrophoresis purification of single-walled CNTs produced by the arc-discharge method. However, CDs went largely unnoticed until the trailblazing works by Sun et al. [15]. Sun prepared carbon nanoparticles and passivated them with diamine-terminated oligomeric poly(ethylene glycol), producing carbon nanoparticles with luminescence emission across the visible range and near-infrared region (Fig. 1). It was then when the term “carbon dots” was coined and used to identify fluorescent carbon nanoparticles. CDs are therefore the newest addition to the carbon family and have become increasingly popular owing to their distinct optical properties, good biocompatibility, low toxicity, facile synthesis, and versatile precursor sources. In view of the advantages offered by CDs, this review will cover the variety of synthesis methods, the structural and optical properties of CDs, and their photoluminescence (PL) mechanisms, followed by the design of sensors, sensing mechanisms, and lastly the latest advances and developments in the application of CDs for the sensing of various analytes. Our intention is for this article to provide a detailed overview and valuable insight into the design and development of CD-based sensors.

## Synthesis of CDs

Generally, the synthetic procedures for CDs can be divided into two main groups, namely top-down and bottom-up methods. Top-down synthesis routes involve breaking or cutting bulk carbon precursors into nanosized particles. Laser ablation, electrochemical oxidation, hydrothermal, and arc-discharge approaches are the common top-down methods employed to prepare CDs. Meanwhile, bottom-up strategies include direct pyrolysis, microwave-assisted pyrolysis, reverse-micelle approaches, and supported route methods. The advantages and disadvantages of the different synthesis methods are summarized in Table 1.

**Table 1:** Summary of the advantages and disadvantages of different synthesis methods

Synthesis method	Advantages	Disadvantages
Laser ablation	Simple experimental setup Can produce particles with different size by changing experimental parameters	Low yield
Arc discharge	Can be obtained from the by-products from purification of carbon nanotubes	Arc-discharged soot contains impurities that are to remove Expensive equipment
Electrochemical approach	Low cost High output Easy manipulation	Doping with heteroatoms is difficult
Chemical oxidation	Requires inexpensive equipment Large-scale production	Tedious steps Toxic reagents Requires strong acid/base
Hydrothermal	Low cost Easy to manipulate parameters such as temperature, time, and pressure of vessel	Long synthesis duration
Pyrolysis of precursors	Simple procedure Short synthesis duration	Difficult to achieve high temperatures for aqueous reaction using domestic microwave oven Simple pyrolysis is difficult to be scaled up due to uneven heating Broad size distribution
Reverse micelles	Can be carry out in room temperature	Complicated steps involved
Supported template	Uniform particle sizes	Complicated steps involved Requires additional passivation steps Difficult to purify

### Arc Discharge

Xu et al. [14] unexpectedly discovered CDs during the purification of single-walled CNTs derived from arc-discharged soot. The soot was first oxidized in 3.3 M HNO_3_ to introduce carboxyl groups, followed by extraction with sodium hydroxide resulting in a stable black suspension. After gel electrophoresis, one fast-moving highly fluorescent band was observed. The fluorescent particles presented a PL quantum yield (QY) of 0.016% and were found to be 18 ± 0.4 nm in diameter. The CDs were comprised of carbon (53.93%), hydrogen (2.56%), nitrogen (1.2%), and oxygen (40.33%). Likewise, Bottini et al. [16] isolated fluorescent nanoparticles from both pristine and nitric-oxidized CNTs prepared by the electric arc method. The fluorescent nanoparticles obtained from pristine CNTs were found to be hydrophobic with a small size distribution. In contrast, the fluorescent nanoparticles obtained from oxidized CNTs had the ability to aggregate when dissolved in water as they were superficially oxidized and/or coated by a thin carbon layer and presented a broader size distribution.

### Laser Ablation

**Fig. 2:**
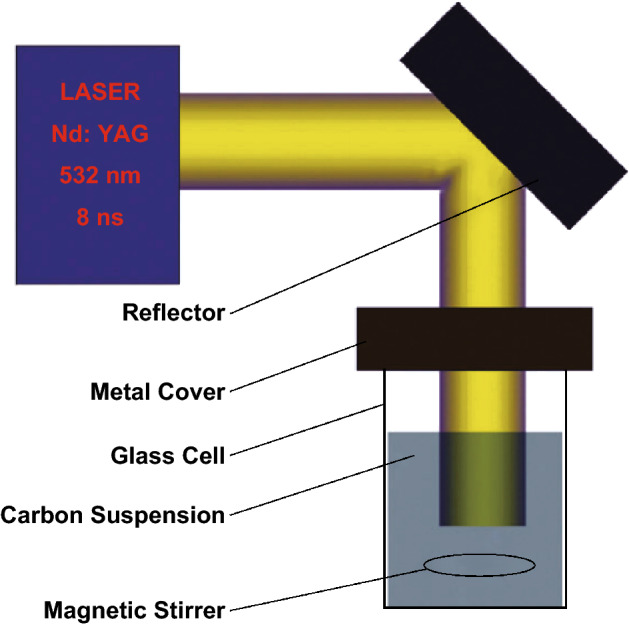
Example of a laser ablation technique experimental setup [18]. Copyright © 2011 The Royal Society of Chemistry

The laser ablation technique to produce CDs was first introduced by Sun et al. In their pioneering work, CDs were produced by exposing a carbon target made of hot-pressed graphite powder and cement to laser ablation in the presence of water vapor and argon as the carrier gas [15]. Similarly, Goncalves reported a simpler one-step facile strategy by irradiating carbon targets in water for 1 min using a pulsed UV laser [17]. The size of the particle could be controlled by the separation between the focusing lens and the carbon target, which determined the incidence area and fluence of the laser. A distance of 107 cm (incidence area = 348 mm^2^, fluence = 115 mJ cm^−2^) afforded carbon particles in the hundred nanometer range, while a shorter distance of 85 cm (incidence area = 139 mm^2^, fluence = 288 mJ cm^−2^) produced carbon particles of ~ 27 nm in size. Based on these reports, it was found that carbon nanoparticles synthesized in the presence of water do not display PL of their own, but rather acquire their PL properties through post-functionalization. Li et al. [18] further investigated the effect of the solvent in which the carbon target was submerged during the laser ablation process on the PL properties using the experimental setup shown in Fig. 2. The group found that the CDs prepared from a carbon target submerged in acetone-exhibited PL, while CDs prepared in water displayed no PL whatsoever. The group suggested that the functional groups generated during the synthesis process on the surface of the CDs were responsible for the observed PL emission.

Similarly, Hu et al. [19] prepared luminescent CDs by irradiating graphite flakes dispersed in a poly(ethylene) glycol solution with a Nd:YAH-pulsed laser with 1064 nm wavelength and power density of 5 × 10^6^ W cm^−2^. The CD dimensions can be manipulated by changing the pulsed size, where smaller pulse widths yield smaller particles and vice versa. When the laser beam hits the graphite flakes, a high-temperature and high-pressure plasma plume forms at the interface of the graphite flakes and the surrounding medium. Due to liquid confinement effects, a bubble forms at the laser focus and rapidly expands to its maximum radius. As the laser pulse goes into the off state, the bubble starts to shrink due to external pressure from the surrounding medium, resulting in the cooling of its inner region and formation of clusters or nuclei. As the interaction time is determined by the pulse width, bubbles containing different nuclei densities can be formed under the same laser energy but different pulse widths. The group further performed thermodynamic calculations, suggesting that higher nuclei densities are advantageous for nucleation as they minimize the distance between nuclei. Furthermore, the movement of nuclei is facilitated in the case of small nuclei, resulting in nuclei coming into close contact upon shrinking of the bubble. As a result, coagulation takes place leading to the formation of larger nuclei. Also, as the bubble size diminishes, the nuclei come into contact with poly(ethylene) glycol molecules, which passivate the particle surface and endow the particle with PL properties. Overall, the PL QY of CDs is influenced by the size distribution, which in turn can be optimized by adjusting the pulse width of the laser.

### Electrochemical Approaches

**Fig. 3:**
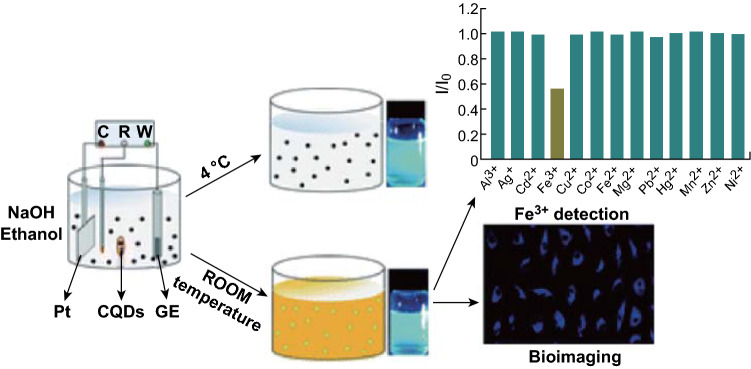
Schematic illustration of CQD generation via electrochemical oxidation of a graphite electrode in alkaline alcohols. The color change of the dispersion during room temperature storage is due to oxygenation of the surface species [21]. Copyright © 2016 The Royal Society of Chemistry. (Color figure online)

In a typical electrochemical synthesis setup, three electrodes are employed: a carbon precursor serves as the working electrode while the remaining two electrodes serve as the counter and reference electrode. Different carbon precursors can be used and the experimental setup can be modified for better performance. Bao et al. [20] utilized carbon fibers as the working electrode with platinum and silver wire as the counter electrode and quasi-reference electrode, respectively. By adjusting the applied potentials, the particle size of the CDs could be well controlled. The as-synthesized CDs presented narrow size distributions without the need for further purification and separation steps. The CDs were suggested to exfoliate from the surface of carbon precursors through a combined effect of the applied voltage and intercalation of the electrolyte into the turbostratic graphite structure of the carbon fibers. In a similar manner, Liu et al. [21] also explored the effect of the applied voltage on the physical and optical properties of carbon quantum dots (CQDs). The average size of CQDs prepared at 3 V was 2.9 nm while, at 7 V, CQDs with an average size of 5.2 nm were obtained. Moreover, the PL QY of CQDs also differed when prepared at different applied voltages. An applied voltage of 3 V yielded CQDs with a PL QY of 9.5%, which was reduced to 4.6% at 7 V. The CQDs exhibited high specificity for ferric ions and can thus be potentially used as a fluorescence sensor for the detection of ferric ions in the environment and in biological imaging (Fig. 3).

On the other hand, Hou et al. [22] proposed a different experimental setup arrangement for the synthesis of CDs. In their report, two electrodes serving as the cathode and anode were submerged in the carbon precursor, which consisted of a mixture of sodium citrate and urea. After applying 1 VDC for 5 h, the transparent mixture turned brown and was further dialyzed to obtain fluorescent CDs with an average size of 2.4 nm and PL QY of 11.9%. The fluorescent CDs exhibited excellent fluorescence stability under extreme pH, high ionic strength, or long illumination exposure. Deng et al. [23] reported the electrochemical synthesis of fluorescent CDs from low molecular weight alcohols, such as ethanol, as the carbon precursors under basic conditions. Two platinum sheets were employed as the working and counter electrodes, while a calomel electrode was used as the reference electrode. The alcohol precursors were mixed with water and sodium hydroxide before applying a voltage potential. Upon CD formation, the solution turned dark brown. Ethanol was added to the mixture overnight to salt out the sodium hydroxide before further purification through dialysis to afford CDs with a PL QY of 15.9%.

### Chemical Oxidation

Chemical oxidation methods usually employ strong oxidants such as concentrated acids to provide an oxidative environment for the treatment of a variety of carbon precursors. Tao et al. [24] prepared CDs via this approach using different carbon precursors such as single-walled CNTs, multiwalled CNTs, and graphite. Sulfuric acid and nitric acid have been used as the oxidizing agent and it is worth noting that the resultant CDs from different precursors exhibited similar size distributions, composition, PL properties, and surface properties. These CDs were found to be biocompatible with low cytotoxicity and are thus suitable for biomedical applications. Sun et al. [25] reported a novel method using human hair fibers as the carbon precursor for the synthesis of sulfur and nitrogen co-doped CDs. Hair fibers were first added to concentrated sulfuric acid, sonicated for 30 min, and stirred for 24 h at 40, 100, or 140 °C. It was found that higher synthesis temperatures favor the development of sulfur and nitrogen and sulfur co-doped CDs with smaller size, higher sulfur composition, and the occurrence of both down- and up-conversion PL. The resultant CDs showed excellent photostability, negligible toxicity, and high biocompatibility and solubility.

Chemical oxidation of green precursors has also been reported to produce homogeneous CDs. Chang et al. [26] reported the synthesis of CDs by oxidizing sucrose with phosphoric acid. The resultant CDs displayed yellow PL (*λ*_em_ = 560 nm) under 360 nm excitation, which remained unaffected in the pH range of 4.0–11.4. The CDs also exhibited high ionic stability and were used for the detection of chromium(III) with a limit of detection (LOD) of 24.6 µM in the range up to 200 µM. Environmental waste has also been explored as the carbon precursor for the chemical oxidation of CDs. Hu et al. [27] developed a one-step synthesis method to prepare sulfur-doped CDs from waste frying oil with concentrated sulfuric acid as the dehydrating and oxidizing agent. The CDs were homogeneous in size and exhibited a partially disordered graphite-like structure. The CDs emitted in the UV region and displayed distinct pH-sensitive PL from pH 3 to 9, making them suitable for application as an optical pH probe. Additionally, their satisfactory optical properties and low toxicity render them as suitable fluorescent probes for biological imaging.

### Hydrothermal Synthesis

Hydrothermal synthesis is one of the simplest, economical, and widely synthetic routes used to prepare CDs. This method has the advantages of cheap instrumentation, low energy consumption, and ease of manipulation of the synthesis parameters for the preparation of homogeneous and high-quality CDs [28–30]. For instance, Xu et al. [31] prepared sulfur-doped CDs from sodium citrate and sodium thiosulfate using a hydrothermal synthesis route. By optimizing the ratio of precursors, temperature, and reaction time, CDs with homogeneous size distributions and good fluorescence properties were obtained. The CDs had an average size of 4.6 nm and high PL QY of up to 67%. Fourier transform infrared spectroscopy revealed a large amount of hydroxyl moieties on the surface of the CDs, endowing them with good aqueous solubility. Li et al. [32] developed the hydrothermal synthesis of citric acid and poly(ethylenimine), which exhibited both up- and down-conversion PL. The CDs were found to exhibit a high PL QY (48.3 ± 5.3%), pH-dependent PL, and excellent photostability and ionic strength. Such outstanding dual-mode PL provided them with the capability to detect morin in human urine samples with high sensitivity of 0.6 µmol L^−1^.

Many research groups have also developed hydrothermal syntheses using green precursors such as beverages [33, 34], fruit extract [35, 36], food products [37–39], and biomass [40, 41]. Sahu et al. [42] reported a one-step hydrothermal treatment of orange juice to prepare highly luminescent CDs. The as-synthesized CDs provided a 26% PL QY with excellent photostability and low toxicity, suitable for application as fluorescent probes in cellular imaging. Wu et al. used *Bombyx mori* silk, which has high nitrogen content, as the carbon and nitrogen source to synthesize nitrogen-doped CDs. Despite the simple steps involved in the hydrothermal synthesis, this preparation route usually requires long synthesis times. This is a major impediment for the large-scale production of CDs. Hence, microwave-assisted hydrothermal synthesis routes have been explored to prepare CDs, as they offer more rapid, uniform, and simple synthesis conditions. Purbia et al. [43] developed a microwave-assisted hydrothermal synthesis using coconut water that was able to afford CDs under a minute. The properties of the as-synthesized CDs could be manipulated by changing the synthesis temperature and duration. The as-synthesized CDs exhibited blue and green PL when excited at 390 and 450 nm, respectively. The CDs were employed as a turn-on fluorescence sensor for thiamine with an LOD range of 10–50 µM.

### Pyrolysis of Precursors

Pyrolysis processes leading to the carbonization of precursors under high temperature have been widely reported for the preparation of CDs. Generally, the CD precursor undergoes several processes, including dehydration, polymerization, carbonization, and subsequent formation of CDs. Niu et al. [44] reported the simple one-step thermal conversion of l-glutamic acid into fluorescent CDs. In a typical reaction, the precursors were heated to 200 °C using a heating mantle for 5 min. The precursors were liquefied and the color of the solution changed from clear to brown, indicating the formation of CDs. Chandra et al. [45] developed a solid-phase pyrolysis route to prepare nitrogen and phosphorus co-doped CDs. Citric acid (the carbon source) was ground with diammonium hydrogen phosphate (nitrogen and phosphate source) at a molar ratio of 1:4 (citric acid/diammonium hydrogen phosphate). The ground salts were then placed in a silica crucible and heated at 180 °C for 1 h in an oven. Subsequently, the carbonized precursors were dissolved with deionized water and purified for use in Fe^3+^ ion sensing. Green precursors, such as watermelon peel, have also been used to synthesize CDs in a similar manner. Briefly, fresh watermelon peels were subjected to heat treatment at 220 °C for 2 h in air for the carbonization process. The obtained product was dispersed in water and sonicated for 30 min before purification via filtration and dialysis. The as-synthesized CDs exhibited excellent water solubility and emitted blue PL with a QY of 7.1%.

Besides pyrolysis of the precursors in the solid state, pyrolysis in liquid phase is another route to prepare fluorescent CDs. It is worth noting that the solvent where the precursors are dissolved in can play a significant role in the synthesis of CDs. Mao et al. [46] performed the one-step heating of poly(acrylic acid) (PAA) with glycerol or water as the solvent to prepare fluorescent CDs. In brief, PAA was dissolved in water or glycerol and subjected to 200 °C for 2 h. After naturally cooling down to room temperature, the obtained CDs were purified. The CDs synthesized using glycerol exhibited white PL, while those prepared using water displayed blue PL. This is due to the role of glycerol, which serves as the solvent as well as a carbonization promoter and surface passivation agent. Wang et al. [47] reported a novel strategy to prepare highly luminescent CDs soluble in oil. The CDs were prepared with anhydrous citric acid as the carbon precursor, octadecene as a noncoordinating solvent, and 1-hexadecylaine as the surface passivation agent. In a typical reaction, 15 mL of octadecene and 1.5 g of 1-hexadecylaine were heated up in a three-neck flask under argon flow, followed by injection of 1 g of citric acid. The CDs could be dissolved in common nonpolar organic solvents such as hexane, chloroform, and toluene with a PL QY of up to 53%. However, when the solvent and passivation agent were replaced with glycerin and PEG_1500_, respectively, the CDs were found to be water-soluble with a much lower PL QY of 17%. These results indicate that the solvents used in the synthesis of CDs influence the physical and optical properties of the as-synthesized CDs. He et al. [48] reported a simple microwave-assisted synthesis route for ultrahigh PL QY CDs using citric acid and ethylenediamine. By using a microwave digestion furnace, the temperature of the reaction solution could be rapidly increased to 200 °C. The CDs were prepared within 5 min exhibiting an ultrahigh PL QY of 96%.

Microwave-assisted pyrolysis has also been widely reported as a facile synthesis route for CDs. This synthesis route has the added advantages of being rapid and providing uniform heating, with the possibility of being carried out using a conventional microwave oven. Li et al. [49] reported the preparation of nitrogen and sulfur co-doped CDs through a one-step microwave-assisted synthesis route. By using ammonium citrate and l-cysteine as precursors, the synthesis was accomplished in 2.5 min using a 750 W microwave domestic oven. The as-synthesized CDs presented a high PL QY of 62% and could be employed for drug sensing. In another report, Xiao et al. [50] prepared nitrogen-doped CDs using citric acid and ethylenediamine as the carbon and nitrogen sources, respectively. The precursor mixture was subjected to low power (80 W) and medium–high temperatures for 15 min. The resultant CDs displayed a high PL QY of 80% and were found to be non-toxic toward nasopharyngeal carcinoma cells and embryonic kidney cells.

### Reverse Micelles

Kwon et al. [51] developed a one-step preparation of graphitic CQDs employing water-in-oil reverse micelles. Briefly, an aqueous glucose solution was mixed with decane under the assistance of bis(2-ethylhexyl) sulfosuccinate sodium salt (AOT) as the surfactant to form water-in-oil reverse micelles. After heating up to 160 °C, condensation–polymerization of the glucose molecules occurred to form oligosaccharides. The micelles would reach a critical supersaturation point due to water evaporation, resulting in the simultaneous carbonization of the oligosaccharides and in situ passivation. The diameter of the CQDs could be controlled by manipulating the water/surfactant molar ratio within the micelles. Zhang et al. [52] reported the synthesis of CQDs by reversible addition–fragmentation chain transfer (RAFT) polymerization. A poly(acryloylglucosamine)(acrylic acid) [P(AGA)(AA)] copolymer was first prepared in aqueous form using the RAFT technique from *N*-acryloyl-d-glucosamine (AGA) as the carbon precursor. Subsequently, heptanol was injected in the mixture and sonicated to form a light-yellow solution. Since P(AGA) is insoluble in heptanol while PAA is soluble, reversed polymeric micelles were formed upon addition of heptanol, where AGA was confined in the micelle-like nanoreactors. The mixture was then pyrolyzed at 170 °C and refluxed for 40 min under nitrogen gas until the mixture turned brown. AGA underwent carbonization to form CQDs in this stage and heptanol was then removed through evaporation under vacuum. Finally, the obtained CQDs were purified by centrifugation and filtration. The surface of CQDs was covered with carboxylic acid moieties, rendering them dispersible in aqueous and polar organic solvents. The size and properties of the as-synthesized CQDs could be tuned by manipulating the amount or AGA/AA ratio. This method of synthesis has potential for scale-up for mass production of CDs using conventional synthesis facilities. In another report, Linehan reported a room-temperature reverse-micelle synthesis of monodisperse, alkyl-capped CQDs [53]. Typically, carbon tetrachloride was added to tetraoctyl ammonium bromide (TOAB) and dispersed in anhydrous toluene, forming reverse micelles where the carbon precursor was confined within the hydrophilic interior of TOAB. The carbon precursor was then reduced with the addition of lithium aluminum hydride to form hydrogen-terminated CQDs confined within the micelle interior. The surface of the CQDs was finally passivated through a platinum-catalyzed process.

### Supported Template Approaches

Template approaches have also been used in the synthesis of CDs with controlled morphologies and properties. This synthesis route consists of two steps: (1) the synthesis of CDs by calcination in a template; and (2) etching of the template to remove the support and release the nanosized CDs. Liu et al. [54] introduced satellite-like polymer/F127/silica composites using silica colloid spheres modified with amphiphilic copolymer F127 as the carrier and resol as the carbon precursor. The composites were subjected to high-temperature treatment and etching to produce nanosized CDs. Further acid treatment and surface passivation resulted in aqueous solubility and multicolor emission with a PL QY of 14.7%. Similarly, Yang et al. [55] developed a soft–hard template approach to synthesize fluorescent CDs with uniform morphology using Pluronic P123 as the soft template and ordered mesoporous silica SBA-15 as the hard template. In their report, different organic precursors, such as pyrene and diaminebenzene, were employed as the carbon sources. After carbonization through a hydrothermal process, the product was purified and dried before further calcination. The CDs were released by etching the silica template with sodium hydroxide and purified through dialysis. Lastly, the CDs were surface-functionalized with nitric acid and passivated with PEG1500 N. The as-synthesized CDs presented tunable sizes, composition, and crystallinity degree with PL QYs ranging 3.3–4.7%. This method allows the preparation of CDs with small size distributions on account of the size confinement within the templates. However, such template approach for the synthesis of CDs involves many tedious steps. Also, a strong acid or alkali is required to etch away the template for the release of CDs and additional passivation steps are required to endow the CDs with fluorescence properties. These drawbacks make this method less desirable as a facile route to prepare fluorescent CDs.

## Properties

### Structural and Compositional Properties

**Fig. 4:**
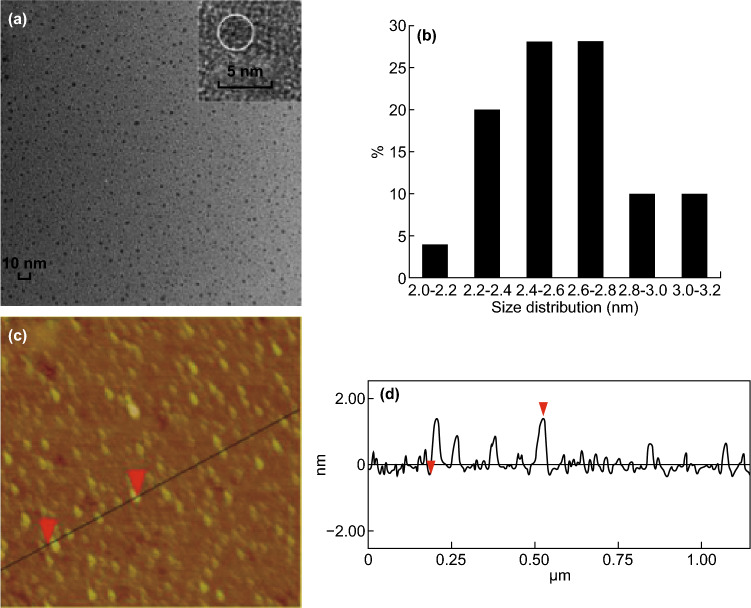
**a** TEM images, **b** size distribution, and **c**, **d** AFM image and profile of CDs [78]. Copyright © 2015 Elsevier Ltd.

CDs, also known as carbon nanodots (CNDs) or CQDs, are quasi-spherical carbon nanoparticles with a particle size below 10 nm and lattice spacing ranging 0.18–0.24 nm [13, 56] (Fig. 4). Generally, they comprise amorphous or nanocrystalline cores with *sp*^2^ clusters [57]. Some studies have also reported diamond-like structures formed by *sp*^3^-hybridized carbon atoms [58]. Qu et al. [59] prepared CDs through a one-step microwave synthesis route that contained both *sp*^2^- and *sp*^3^-hybridized carbon atoms. Raman spectroscopy measurements were carried out to characterize the as-synthesized CDs and two broad bands were observed at 1365 and 1575 cm^−1^ originating from the D band (*sp*^3^-hybridization) and G band (*sp*^2^-hybridization), respectively. The D band corresponds to the vibration of carbon atoms with incomplete bonds in the termination plane of disordered graphite, while the G band is associated with the *E*_2g_ mode of graphite and the vibration of *sp*^2^-hybridized carbon atoms in a two-dimensional hexagonal lattice. The group further confirmed the presence of both types of carbon hybridization through ^13^C NMR spectroscopy, where the spectrum signals in the range of 30–45 ppm correspond to *sp*^3^-hybridized carbon atoms while signals at 90–185 ppm are associated with *sp*^2^-hybridized carbon atoms. The relative intensity (*I*_D_/*I*_G_) of the spectrum bands refers to the in-plane crystal domain size, which is often used to evaluate the degree of graphitization and crystallization of the core [60].

Surface functional groups can be determined using Fourier transform infrared spectroscopy or X-ray photoelectron spectroscopy. Typically, moieties such as C–O, C=O and C–OH can exist on the surface and are usually introduced from the precursors during the synthesis process. In addition, specific functional groups can also be introduced on the surface of CDs by selecting suitable carbon precursors or dopant precursors for the synthesis process. Dong et al. prepared CQDs via low-temperature pyrolysis of citric acid and branched polyethylenimine (BPEI). The resultant CQDs were capped with amino-abundant BPEI on the surface in this one-step process [61]. CDs can also be functionalized post-synthesis, which usually involves multiple steps. Goncalves adopted a three-step process to functionalize CDs prepared by laser ablation of carbon targets submerged in water [17]. First, CDs were activated under reflux with nitric acid for 12 h. Subsequently, the CD solution was refluxed with PEG_200_ for another 28 h. Lastly, the solution was modified with *N*-acetyl-l-cysteine for another 31 h. The obtained CD solution changed from colorless to yellow-brown and was further employed for mercury ion sensing. The surface groups are vital for the modulation of the CD properties and determination of their specificity during the detection of target substances. The surface functional groups can also be used to improve the PL properties. Liu et al. demonstrated PL enhancement of CDs by passivating the surface with polyethyleneimine. The nitrogen-rich polyethyleneimine served both as a passivation agent and a polyelectrolyte to condense DNA for gene therapy [62].

Element doping is another method to modify and improve the properties of CDs, especially the PL QY. The addition of heteroatoms such as nitrogen is known to improve the PL QYs [63]. Other heteroatoms, such as sulfur, phosphorus, boron, silicon, and gadolinium have been incorporated to tune the properties of CDs or insert new functionalities [25, 31, 64–67]. As an example, Bourlinos et al. [64] demonstrated that CDs doped with boron can significantly enhance the nonlinear optical response compared to un-doped CDs. On the other hand, Gong et al. [67] introduced gadolinium in the synthesis of CDs and the resultant products could be used as fluorescent labels and magnetic resonance imaging contrast agents. Co-doping CDs are another facile method to enhance the properties of CDs. Co-doping can be categorized in terms of the type of heteroatom being doped, which mainly refers to nitrogen and sulfur co-doped or nitrogen and phosphorus co-doped CDs. Li et al. [59, 68] prepared sulfur and nitrogen co-doped CDs through a one-step microwave-mediated synthesis using citric acid and thiourea as the precursors, which presented significantly higher PL QYs compared to CDs doped with nitrogen only.

### Optical Properties

In general, CDs are effective in absorbing light in the short-wavelength region of 230–340 nm with a tail extending into the visible region. The first absorption band located around 230–280 nm is associated with *π*–π* transitions of the C=C bonds of the carbon core, while the second band at around 300–340 nm is due to *n*–π* transitions of C=O surface groups [69, 70]. The absorption spectrum can be modulated by heteroatom doping and surface passivation. For instance, Das et al. [71] prepared three different types of doped CQDs, namely BCQDs (doped with boron), BNCQDs (co-doped with boron and nitrogen), and BNSCQDs (co-doped with boron, nitrogen, and sulfur). Only BNSCQDs were found to present three bands centered at 260, 320, and 440 nm, while BCQDs and BNQDs only exhibited two bands. The two higher energy bands of BNSCQDs correspond to *π*–π* and *n*–π* transitions of the aromatic *sp*^2^ domains and nonbonding orbitals, respectively, while the transition band at 440 nm is associated with the trapping of excited state energy by certain surface states.

**Fig. 5:**
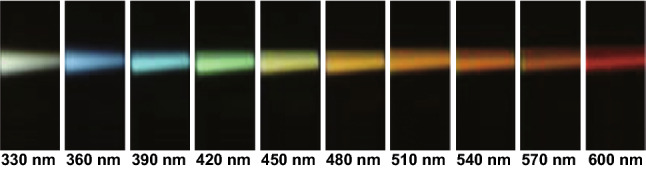
PL emission of CDs excited from 330 to 600 nm in 30 nm increments [73]. Copyright © 2015 WILEY–VCH Verlag GmbH & Co. KGaA, Weinheim

One of the most interesting optical properties of CDs is their tunable PL across the entire visible spectrum. Jiang et al. prepared CDs with blue, green, and red emission from distinctive phenylenediamines isomers using 365 nm excitation. The different precursors employed resulted in CDs with different sizes and nitrogen contents, leading to differences in the PL emission [72]. Typically, CDs display excitation-dependent PL. The PL bands red-shift with the increasing excitation wavelength. Pan et al. [73] prepared excitation-dependent CDs using a facile one-step microwave-assisted pyrolysis treatment of a citric acid–formamide mixture. As the excitation wavelength was increased from 330 to 600 nm, the corresponding emission covered almost the entire visible spectrum, as shown in Fig. 5. Interestingly, CDs have also been reported to exhibit excitation-independent PL emission. Zhang et al. [74] reported that the emission wavelength of hydrothermally synthesized CDs at 418 nm remained unchanged with the increasing excitation wavelength. This phenomenon is attributed to uniform surface moieties, which are responsible for the CD emission. By controlling the synthesis parameters, such as the synthesis time and temperature, the surface properties can be modulated, thus systematically controlling the emission spectrum. Li et al. [75] prepared CDs from citric acid and urea by a hydrothermal method at different temperatures. In this report, the emission was tuned to be excitation-dependent or excitation-independent by changing the synthesis temperature. It was found that CDs prepared at 160 °C were excitation-independent due to suitable passivation of the surface traps on the CDs, thus leading to a single transition mode. When the CDs were synthesized at 240 °C, the surface was passivated with a much smaller number of amino groups, resulting in surface traps with multiple energy levels, subsequently leading to excitation-dependent PL emission.

**Fig. 6:**
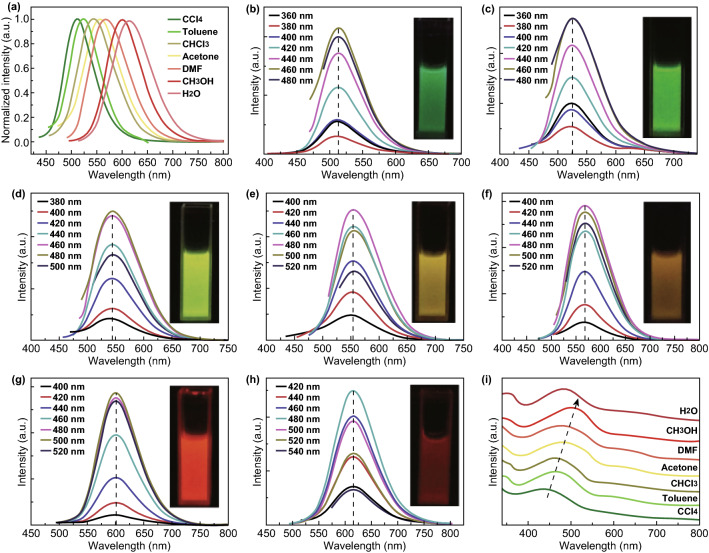
PL spectra of CDs prepared from *p*-phenylenediamine dispersed in different solvents. CDs dispersed in **a** tetrachloromethane, **b** toluene, **c** chloroform, **d** acetone, **e** dimethyl formamide, **f** ethanol, and **g** water, and **h** absorption spectra of the CDs in different solvents [76]. Copyright © 2017 The Royal Society of Chemistry

**Fig. 7:**
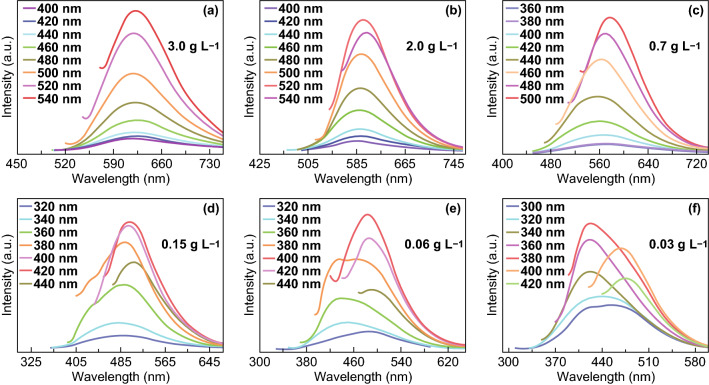
PL emission peak blue-shifting as the concentration was reduced from 3.0 to 0.03 g L^−1^ [77]. Copyright © 2017 The Royal Society of Chemistry. (Color figure online)

CDs have also been reported to exhibit solvent-dependent PL. Wang et al. [76] found that CDs synthesized from *p*-phenylenediamine could emit at wavelengths ranging from green to red, independent of the excitation wavelength, when dispersed in different solvents. The as-synthesized CDs emitted green PL when dispersed in tetrachloromethane, while the PL emission was red-shifted when the CDs were dispersed in other solvents (Fig. 6). This phenomenon is attributed to intramolecular charge transfer and is useful in optoelectronic applications as all colors can be obtained from a single type of CDs. Additionally, CDs may also exhibit concentration-dependent PL. As the concentration of CDs was reduced from 3.0 to 0.03 g L^−1^, the PL emission peak gradually shifted from 630 to 400 nm, as shown in Fig. 7 [77]. When CDs are dispersed in high-concentration solvents, the distance between CD particles is extremely short and with high surface energy. Similar to crystallization of an oversaturated solution, aggregation of CDs can minimize the distance between CDs and reduce the supersaturation degree. As the concentration of CDs decreases with the increasing solvent volume, intramolecular interactions induce the CD aggregates to dissolve. These concentration-induced morphological changes can affect the energy traps associated with the surface state.

The PL properties of CDs can also be influenced by the pH value. Wang et al. [78] reported that CDs showed increasing PL intensity with the gradual increase of the pH, where the maximum intensity was recorded at pH 3. Subsequently, the PL intensity dramatically decreased with the increasing pH from 4 to 13. The group elucidated this phenomenon by analyzing the changes in the particle size by transmission electron microscopy. CDs exist as isolated particles at low pH values, but aggregate at high pH values due to noncovalent molecular interactions, resulting in fluorescence quenching. Other groups have also correlated pH-dependent PL to the protonation and deprotonation of the surface functional groups [79–81]. With the increasing pH value, the surface charge of CDs may can change from positively to negatively due to the dissociation of amine and oxygenated surface functional moieties, such as carboxyl and hydroxyl, resulting in modulation of the PL properties in solvents with different pH. Li et al. [82] showed that the resultant CDs displayed strong emission at pH 7.0, but that the PL was almost quenched completely under strong alkaline or acidic conditions. The PL intensity was recovered when the pH was restored to pH 7.0.

Furthermore, the temperature has also a noteworthy effect on the PL intensity of CDs. Yu et al. [83] reported that the PL intensity of CDs decreased with the increasing temperature. This can be potentially attributed to thermal activation of non-radiative trapping. At low temperatures, the non-radiative channel is not activated; hence, PL with higher intensity is observed. However, as the temperature increases, non-radiative channels such as trapping are thermally activated by surface groups, defects, and dopants. The non-radiative lifetime decreases with the increasing temperature, resulting in a reduction in the quantum efficiency and fluorescence intensity. In a separate report, Chen et al. [84] developed organosilane-functionalized CDs responsive to temperature changes. CDs were prepared using a one-step hydrothermal synthesis route to obtain highly fluorescent CDs with excellent solubility. Similarly, the reduction in the PL intensity at elevated temperatures was attributed to the activation of non-radiative pathways. The proposed sensor had a linear operating range from 293 to 343 K. Such properties confirm the potential of CDs as temperature sensors.

In contrast to the usually observed down-conversion PL emission, CDs are also known to display up-conversion PL (UCPL). Cao et al. [85] first reported UCPL in as-synthesized CDs with a two-photon absorption cross section of 39,000 ± 5000 GM, which were able to emit green PL upon excitation by 800 nm laser pulses. The group further exploited the UCPL properties for two-photon imaging of human breast cancer cells. Since then, UCPL has gained much attention as a favorable quality for various applications, particularly for bioimaging and photo-mediated therapy for cancer treatment. UCPL employs near-infrared (NIR) light as the excitation source and has the virtues of having deeper tissue penetration depth, reduced tissue scattering, and minimal photo-damage to the surrounding tissues. In sensing applications, CDs with UCPL properties have the additional advantage of providing a dual readout channel, increasing their selectivity toward the target ions. For instance, Castillo et al. [86] found that their CDs were responsive toward copper and iron metal ions when evaluating their down-conversion PL signals. However, only copper ions had an influence on the UCPL signals, which indirectly assisted the determination of the type of metal ion present. The dual-mode readout channel is also capable of widening the response range. Yin et al. [87] prepared green CDs from sweet pepper as the carbon source with a PL QY of 19.3% for the detection of the oxidizing substance ClO^−^. Both the down-conversion PL and UCPL signals of the as-prepared CDs increased with the decreasing concentration of ClO^−^. The detection range was 0.1–10 µmol L^−1^ with an LOD of 0.05 µmol L^−1^ using down-conversion PL, while the detection range was 10–300 µmol L^−1^ with an LOD of 0.06 µmol L^−1^ using UCPL.

**Fig. 8:**
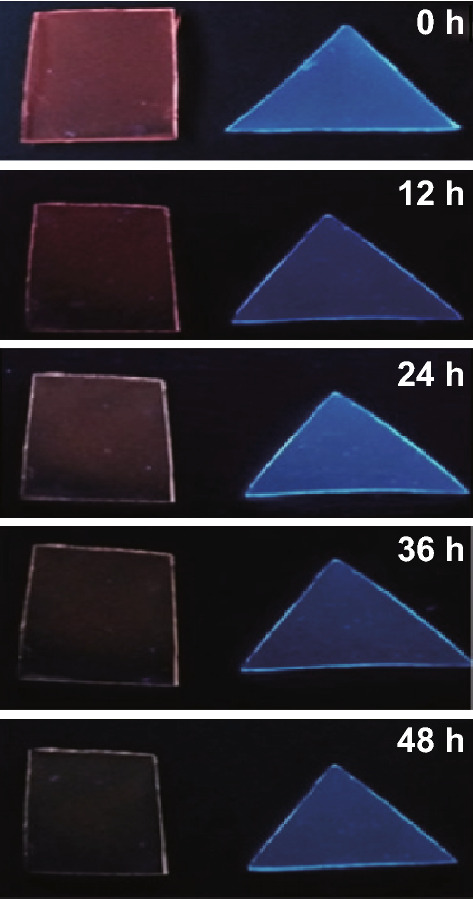
Photographs of a CdTe film and a CD epoxy resin film under UV illumination taken at different time intervals [88]. Copyright © 2016 The Authors. Published by Elsevier B.V.

The excellent stability of CDs further contributes to the wide applications of CDs. Photobleaching is a major setback for many fluorophores, in which the PL degrades over time with or without continuous excitation, limiting the shelf-life of the fluorophores and long-term imaging. In a study carried out by Zhang et al. [88], the photostability of CDs was evaluated and compared to that of CdTe QDs. Water-soluble CDs were combined with poly(ethylene glycol) diglycidyl ether and diaminooctane to form an epoxy resin film. Compared to the CdTe QD film, the CD film only afforded a QY decay of 5% after 48 h of continuous illumination (Fig. 8). This study showed that CDs have excellent photostability and their applications can thus be extended to other biological areas such as long-term imaging.

## Photoluminescence Mechanisms

**Fig. 9:**
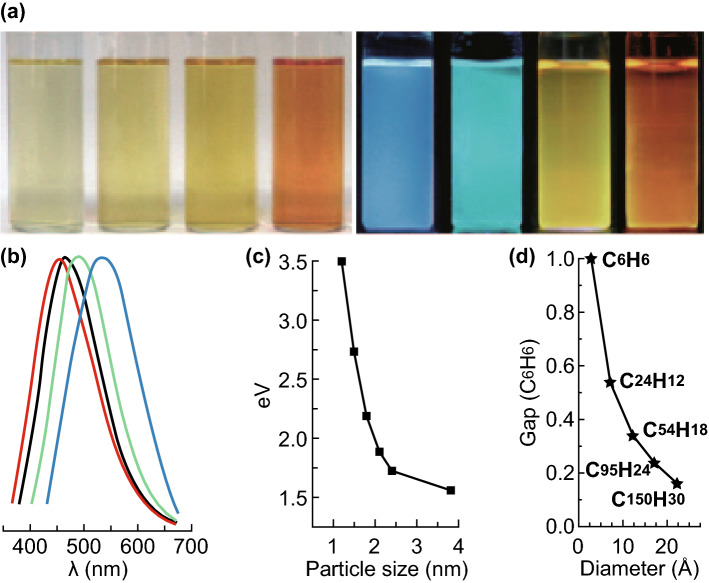
a Photographs of CQDs with increasing particle dimensions under (left) white and (right) UV light, **b** PL spectrum of CQDs with emission from blue to red, **c** relationship between the CQD particle size and PL properties, and **d** relationship between the HOMO–LUMO bandgap and the size of the graphite fragments [90]. Copyright © 2010 WILEY–VCH Verlag GmbH & Co. KGaA, Weinheim. (Color figure online)

Depending on the composition, bulk materials are characterized by their bandgap energy, which is defined as the minimum energy needed to excite an electron from the ground state to the vacant conduction band. Upon absorbing energy larger than the bandgap, electrons are excited into the conduction band, producing excitons that consist of an electron–hole pair. Subsequently, these excitons are annihilated with concomitant emission of photons, also known as radiative recombination [89]. Excitons have a finite size in the nm range, as defined by the Bohr exciton radius [12]. If particles are smaller than the excitons, their charge carriers become spatially confined within the particles, giving rise to a PL effect due to a phenomenon known as quantum confinement. Due to their particle size, which is in the nanometer range, CDs also experience said quantum confinement effect, becoming one of the factors determining their tunable PL properties. Li et al. reported the effect of the particle size on the PL properties of CQDs. In their report, three different sizes of CQDs were prepared through a one-step alkali-assisted electrochemical method [90]. Small-sized CQDs had a size of 1.2 nm and medium-sized CQDs of 1.5–3 nm, while large-sized CQDs presented a size of 3.8 nm. Small CQDs emitted in the UV range, medium CQDs in the visible range, and large CQDs in the NIR region, as shown in Fig. 9a, b. To further elucidate the quantum-sized properties, theoretical density functional calculations were carried out to study the relationship between the size of CDs and their luminescence characteristics. As shown in Fig. 9c, d, the bandgap increases with the decreasing particle size. Bandgap energies in the visible range can be obtained from graphene fragments with sizes of 14–22 Å, similar to the visible-light emitted by CDs with diameters of < 3 nm. Contrary, Kwon et al. [91] reported the blue-shifting of PL with the increasing particle size, also known as inverse PL shift. The group suggested that their CNDs comprised *sp*^2^ clusters with defined energy gaps and oleyamine ligands on the surface acting as auxochromes to reduce the energy gap, leading to inverse PL phenomena.

**Fig. 10:**
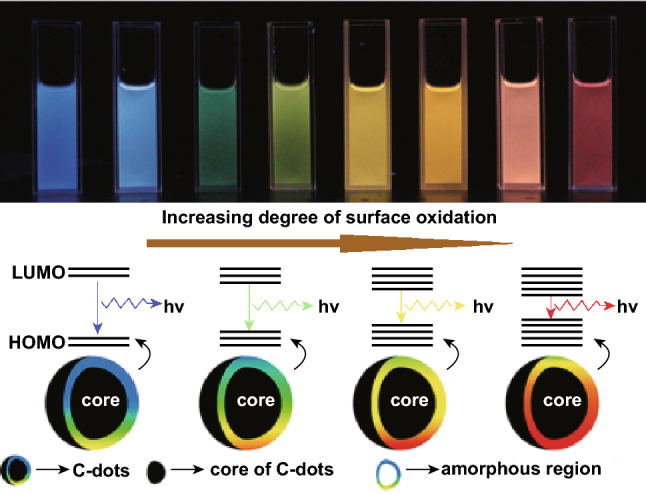
**a** CD samples with increasing surface oxidation (from left to right) imaged under 365 nm UV light irradiation, and **b** model for the tunable PL of CDs with the increasing level of oxidation (from left to right) [93]. Copyright © 2015 American Chemical Society

**Fig. 11:**
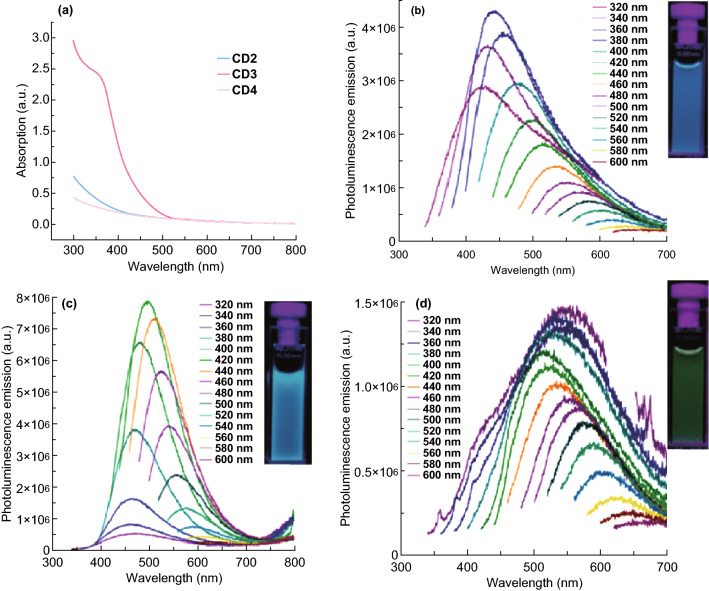
**a** Absorption spectra of CD2, CD3, and CD4; and PL spectra of **b** CD2, **c** CD3, and **d** CD4. The inset image corresponds to CDs under 365 nm UV light illumination [94]. Copyright © 2010 American Chemical Society

The functional moieties found on the surface of CDs during or post-synthesis can present diverse energy levels, which may act as excitation energy traps with different emissions. The surface states that serve as surface defects do not comprise a single unique chemical group but rather a hybrid of the carbon backbone and linked chemical groups [92]. Upon light illumination at a certain wavelength, the corresponding emissive traps dominate the emission, resulting in excitation-dependent PL. On the other hand, if the surface states are uniform or fully passivated, emission will only occur through radiative transitions of the *sp*^2^ carbons. This leads to excitation-independent CDs as a result of a single transition mode with a certain energy. Li et al. [75] reported the preparation for both excitation-dependent and excitation-independent CDs by varying the passivation degree of the CDs. Urea serves as the carbon source as well as provider of amino groups for surface passivation. The amino groups are sensitive to the temperature and tend to detach from the surface of CDs at high temperatures. The group found that CDs synthesized at higher temperatures present more surface traps, which afford their excitation-dependent characteristics. Contrarily, CDs prepared at lower temperatures had a fully passivated surface and excitation-independent emission properties. Ding et al. [93] investigated the influence of surface oxidation on the emission characteristics of CDs. CDs were hydrothermally prepared in one pot using urea and *p*-phenylenediamine as the precursors, and the resultant CDs were carefully separated using silica column chromatography into eight CD samples with distinct fluorescence characteristics and emission ranging from 440 to 625 nm (Fig. 10). All CD samples were found to exhibit excitation-independent PL and comparable monoexponential fluorescence lifetimes. The particle size distribution and graphitic structure of the core were also similar across the different CD samples. The only difference between all the CD samples was the degree of surface oxidation. The group explained the red-shift of the emission peak as a result of the gradual reduction in the bandgap upon incorporation of oxygen species on their surface. Modification of the surface state of CDs during or post-synthesis can have a beneficial effect on the optical properties of CDs. The effect of surface passivation with different passivation agents was investigated by Li et al. [94]. In their report, CDs were passivated with three different types of passivation polymers, namely polyethylene glycol (PEG) chains (CD2), polyethylenimide-*co*-polyethylene glycol-*co*-polyethylenimide copolymers (CD3), and four-armed PEG molecules (CD4). CDs with different passivation agents showed different absorption and emission peaks, as shown in Fig. 11.

**Fig. 12:**
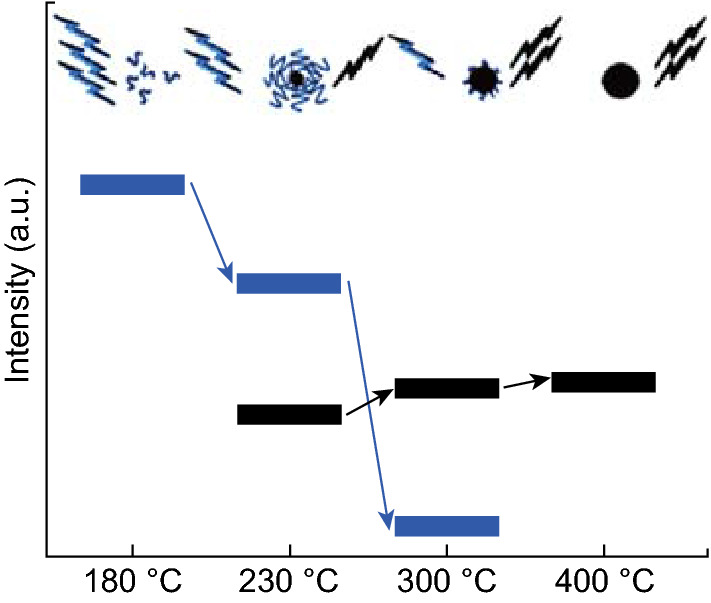
Schematic representation of the emission properties of three photoactive species obtained from heat treatment of a citric acid and ethanolamine mixture. During heating, the organic fluorophores (blue groups) are consumed to build up the carbogenic core (black sphere) so that the PL component that corresponds to the carbogenic core (black bars) increases at the expense of the component from the organic fluorophores [95]. Copyright © 2011 American Chemical Society. (Color figure online)

The down-conversion PL of CDs has also been reported to originate from fluorophore species found on the surface or interior of the carbon core able to emit PL directly. This mechanism is commonly associated with CDs prepared by bottom-up carbonization routes. Krysmann et al. [95] investigated the formation of fluorophores in carbon dots with citric acid and ethanolamine as the precursors, as shown in Fig. 12. When the precursors were pyrolyzed at low temperature (180 °C), strong intense PL with high PL QY was observed. As the temperature increased (230 °C), a carbogenic core was formed by consumption of the molecular fluorophores. During this process, PL may emerge from both the fluorophores and carbogenic core. Ultimately, the molecular fluorophores will be completely consumed and PL will exclusively originate from the carbogenic core. The PL intensity of the CDs depends on the pyrolysis temperature, as well as the amount of fluorophores and the carbogenic core. Despite the tremendous efforts channeled into understanding the PL mechanism, the actual mechanism for CDs remains under debate. The observed PL properties may originate from a combination of the proposed mechanisms, rather than a single unique mechanism.

In addition to down-conversion PL, some CDs have been reported to exhibit UCPL. UCPL is an anti-Stokes phenomenon where the emitted radiation wavelength is shorter and of higher energy than the excitation wavelength, which is significantly advantageous in biological applications. Cao et al. [85] first observed visible-light emission from their CDs upon excitation by an 800-nm pulsed laser, suggesting that those CDs exhibited UCPL. Following this, many other research groups have also observed UCPL in CDs prepared in different manners. For instance, Ma et al. [96] reported that CDs prepared by one-step ultrasonic treatment of glucose and ammonia for 24 h at room temperature exhibited excitation-dependent UCPL. Upon excitation of NIR light ranging from 650 to 1000 nm, UCPL was observed in the range from 300 to 600 nm. This phenomenon has been attributed to a multiphoton absorption process, which is one of the mechanisms commonly associated with nanoparticles exhibiting UCPL.

However, Wen et al. [97] reported that UCPL observed from CDs does not originate from a multiphoton absorption process. Instead, UCPL observed from CDs originates from normal down-conversion PL excited by the second-order maximum of light, which is half of the designated excitation wavelength. For example, when using an 800-nm wavelength as the excitation light source, a 400-nm wavelength, which is weaker, is also present and excites the CDs simultaneously. Thus, the observed PL is the sum of the fluorescence excited by both 800 and 400 nm radiation. In order to circumvent this problem, a long-pass filter can be introduced in the excitation pathway to allow only long wavelengths to pass through. The feasibility of this method was further confirmed by investigating the UCPL of CDs prepared by different methods. The CDs exhibited UCPL when excited at 800 nm, but such UCPL disappeared when a long-pass filter was used for the measurements. In view of the controverting opinions on UCPL, extra precaution should be taken during the interpretation of the UCPL emission of CDs. Moreover, more studies should be carried out to evaluate the effects of precursors, dopants, synthesis route, and functionalization on the UCPL of CDs.

## Sensor Design

In theory, any PL changes, including the PL intensity, wavelength, anisotropy, or lifetime, in the presence of different concentration of specific analytes have potential for application in fluorescence-based sensing. To allow for interaction/binding with analytes, the surface of CDs needs to contain a specific chelating agent able to bind a specific analyte. Generally, the chelating agents on the surface of CDs can be designed through the introduction of functional groups during the synthesis process, post-functionalization, or integration with other molecules such as quenchers or fluorophores. Generally, most synthetic routes introduce functional groups on the surface of CDs, either from the precursors or solvent. For instance, in the hydrothermal synthesis of nitrogen-doped CDs from *P. avium* fruits, the carbonyl groups in the precursors react with ammonia forming ammonium salts [35]. At high temperature, the hydroxyl groups are dehydrated and converted into furfural derivatives. Subsequently, these derivatives polymerize and condensate, producing water-soluble polymers that undergo carbonization via aldol condensation, cycloaddition, and furan resins, producing fluorescent CDs. The as-synthesized CDs were decorated with functional groups such as carboxylic and hydroxyl groups, serving as a passivation layer and endowing the CDs with high water solubility and stability. At the same time, these functional groups can act as chelating agents for Fe^3+^ metal ion sensing. Similarly, CDs prepared through microwave-assisted synthesis, electrochemical synthesis routes, chemical oxidation, and other synthetic methods can be decorated with functional groups that have specific binding affinity toward targeted analytes.

Post-functionalization is another strategy to design CD-based fluorescence sensors. It is known that specific chelating groups/molecules that serve as chelating agents need to be present on the surface of CDs for high specificity and sensitivity of a targeted analyte. For example, Jiang et al. [98] developed a post-functionalized CQD-based fluorescence sensor for glucose detection. Nitrogen-doped CQDs were first prepared through a hydrothermal synthesis route. After purification and acid oxidation, the as-prepared CQDs were incubated with 3-aminophenylboronic acid (APBA) and 1-ethyl-3-(3-dimethylaminopropyl) carbodiimide (EDC), forming an APBA-NCQD nanocomplex. The covalent interactions between phenylboronic acid and hydroxyl groups of glucose led to photoelectron transfer and intrinsic PL quenching of the APBA-NCQD nanocomplex. In a separate report, Zhang et al. [99] functionalized CDs with a quinoline derivative through carbodiimide chemistry. The resultant nanocomplex presented abundant chelating agents on the CD surface, leading to fast response times and high selectivity toward Zn^2+^ metal ions.

Typically, turn-on CD-based fluorescence sensors require additional integration steps with other ions or molecules [100–103]. Yuan et al. [104] reported a turn-on mercury ion sensor using bis(dithiocarbamato) copper(II) (CuDTC_2_)-functionalized carbon. CDs were functionalized with CuDTC_2_ by condensation of carbon disulfide on the nitrogen atoms of the surface functional groups, followed by coordination of ammonium *N*-(dithicarbaxy) sarcosine to form the CuDTC_2_-CD nanocomplex. The CuDTC_2_ complexes on the surface of the CDs are able to quench the PL by electron transfer and energy transfer mechanisms. After addition of mercury ions, Cu^2+^ is promptly displaced from the CuDTC_2_-CD nanocomplex, disrupting the energy transfer path and turning on the PL of the CDs. In another study, Cayuela et al. [105] first reported a turn-off CD-based fluorescence sensor for gold nanoparticles (AuNPs). The group first prepared CDs by refluxing multiwalled CNTs in concentrated acid before functionalization with cysteamine for the detection of AuNPs. The thiol moieties introduced on the surface of the CDs displayed great affinity for gold atoms, forming a CD-AuNP nanohybrid that resulted in quenching of the CD fluorescence.

## Sensing Principles

The basis of PL changes in CD-based fluorescence sensing can be attributed to three major mechanisms, namely photo-induced electron transfer (PET), Forster resonance energy transfer (FRET), and inner filter effect (IFE). Static and dynamic quenching mechanisms are behind PET and FRET processes. In static quenching, a non-fluorescent ground-state nanocomplex is formed through binding of the CDs and target analyte. The excited nanocomplex can return to the ground state without the emission of photons. Several criteria can be associated with a static quenching mechanism: (1) the lifetime of CDs does not change, (2) the formation of the ground-state nanocomplex affects the absorption spectrum of the CDs, and (3) increased temperatures reduce the stability of the ground-state nanocomplex, thus reducing the static quenching. On the other hand, dynamic quenching can be explained by excited CDs returning to the ground state upon collision between the analytes and CDs, resulting in energy transfer. As dynamic quenching only happens in the excited state, no changes to the absorption spectra are observed. This process also affects the lifetime of CDs. IFE itself is not a quenching mechanism but rather a light attenuation phenomenon, where excitation or emission light is absorbed by an excess of CDs or analytes in solution.

### Photo-Induced Electron Transfer

In its commonest manifestation, PET can be illustrated when a complex is formed between an electron donor molecule and an electron-acceptor molecule. The excited complex can return to the ground state without the release of photons. PET processes can be divided into oxidative PET and reductive PET. In reductive PET, CDs act as an electron acceptor for the target analyte, which in turn serves as the electron donor. Electrons are transferred from the highest occupied molecular orbital (HOMO) of the target analytes to the HOMO of CDs. In contrast, CDs serve as the electron donor to the target analyte in oxidative PET processes. The excited electrons are transferred from the lowest unoccupied molecular orbitals (LUMO) of CDs to the HOMO of the electron-acceptor analyte. PET processes result in a decrease in lifetime. Another factor related to a PET quenching mechanism is the existence of an energy gap between the LUMO and HOMO or LUMO and LUMO of the CDs and target analyte, respectively. PET is one of the most commonly used mechanisms to describe quenching phenomena because CDs have functional groups generated on the surface during the synthesis process or from post-functionalization, which allows their interaction with target analytes.

### Forster Resonance Energy Transfer

**Fig. 13:**
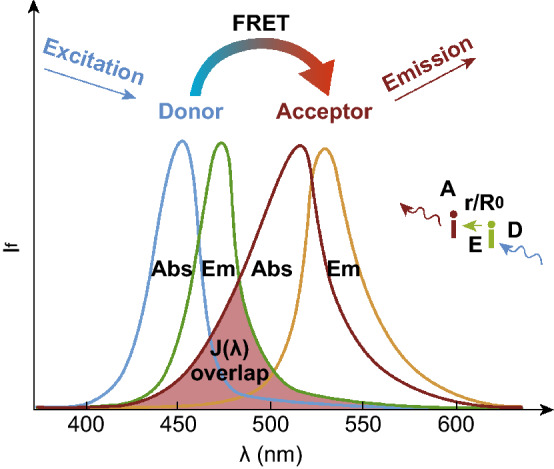
Basic principles of the FRET process. A donor fluorophore (D) is excited and the energy is transferred in a distance and orientation-dependent manner (*r*/*R*_o_) to an acceptor molecule (A). The energy transfer occurs through non-radiative dipole–dipole coupling (E). The acceptor, if a fluorescent molecule, will emit fluorescence but at a lower energy and longer wavelength [267]. Copyright © 2017 Elsevier Ltd.

FRET is another mechanism commonly used in CD-based fluorescence sensors (Fig. 13). FRET is a non-radiative energy transfer process originating from dipole–dipole interactions between donor and acceptor molecules [106]. In a typical FRET process, the absorption of light excites the donor molecule to the LUMO. The donor then returns to the ground state by simultaneously transferring the energy to excite the acceptor molecule. Hence, one of the requisites of this system is that the donor emission spectrum must overlap the acceptor absorption spectrum. The FRET efficiency is inversely proportional to the sixth power of the separation distance between the donor and acceptor, which is typically in the 60–220 Å range [107]. Hence, the donor and acceptor ought to be in close vicinity. In many works, CDs are claimed to be a potential candidate for FRET-based sensing as the PL emission of CDs can be tuned to a wide spectral range with PL QYs as high as 99% [108]; thus, they can be used to match the absorption spectrum of the target analytes. The target analytes (acceptor) can be fluorescent or non-fluorescent. Heat will be dissipated if the acceptor is non-fluorescent, leading to increased dynamic quenching.

### Inner Filter Effect

**Fig. 14:**
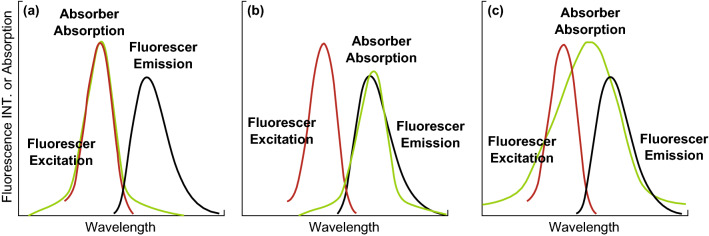
Requisites for IFE: **a** the absorber spectrum overlaps the fluorescer excitation spectrum, **b** the absorber spectrum overlaps the fluorescer emission spectrum, and **c** the absorber spectrum overlaps the fluorescer excitation and emission spectra [110]. Copyright © 2017 Elsevier B.V.

Stokes observed IFE by noticing that the blue PL emitted from a concentrated solution of quinine originated only from the irradiated solution surface [109]. This is due to the concentrated quinine, which has high optical density, absorbing all the excitation UV light in the first few millimeters. Nevertheless, such fluorescence attenuation is negligible in solutions with low optical density. The PL intensity is proportional to the excitation light, and the PL QY is the lowest for an infinitely diluted solution. This is known as IFE. Initially, IFE was considered as a contributing error in fluorometric studies and the optical density of the measurement samples was of utmost importance to minimize the effects of IFE. However, many research groups have exploited this phenomenon as a potential alternative for fluorescence sensing. Any reduction to the PL of the fluorophores is related to the presence of different concentrations of analytes. The attenuation of light in a fluorescence sensor can be attributed to primary or secondary IFE. Primary IFE refers to the absorption of excitation energy by chromophores in the solution, while secondary IFE refers to the absorption of emitted light by the same chromophores. In both cases, IFE leads to a reduction in the PL intensity without affecting the decay time. On top of this, since IFE is neither a static nor dynamic quenching process and no new complexes are formed, the absorption peaks of the fluorescer are not affected. There are several prerequisites for the construction of an IFE-based fluorescence sensor consisting of an absorber and fluorescer (Fig. 14) [110]: (1) the absorption spectrum of the absorber must present sufficient overlap with the excitation and/or emission spectrum of the fluorescer, which allows the absorber to influence the PL of the fluorescer; (2) the absorber must exhibit sensitive and selective response to the presence of the analyte; and (3) the optical properties of the fluorescer and absorber must be independent of the analyte. Hence, careful selection of the absorber and fluorescer is required, such that the excitation and/or emission intensity of the fluorescer can be modulated in the presence of the target analytes.

## Sensing Applications

Their excellent PL properties, as well their functional groups for interaction with analytes, have rendered CDs an excellent candidate for the detection of heavy metals, cations, anions, biomolecules, biomarkers, nitroaromatic explosives, pollutants, vitamins, and drugs. In the following sections, we will highlight the latest developments on CDs for sensing applications.

### Ferric Ion

Iron deficiency is the most widespread dietary deficiency in the world, having adverse impact on the work capacity and motor and mental growth in newborns, children, and adolescents. Conversely, excessive intake of iron can lead to diseases such as hemochromatosis, an iron storage ailment related to excessive oxidative stress. Hence, a simple and facile manner to monitor quantitatively and qualitatively the amount of iron in ecosystems and living systems is of paramount importance. To date, iron is the most widely sensed metal ion in terms of CD-based fluorescence sensors. Table 2 summarizes the recent reports on CD-based fluorescence sensing [38, 111–125]. The applications of CDs for the detection of iron ions are mainly due to CD fluorescence quenching, where the extent of fluorescence quenching is correlated to the amount of iron ions present. Gong et al. [111] prepared nitrogen-doped CDs by microwave-assisted heating of chitosan, acetic acid, and 1,2-ethylenediamine, which serve as the carbon precursor, condensation agent, and nitrogen source, respectively. Due to the special coordination interaction between Fe^3+^ ions and the hydroxyl and/or amine groups of CDs, Fe^3+^ ions can selectively and sensitively quench the CD fluorescence with an LOD of 10 parts per billion (ppb). Shangguan et al. [112] reported nitrogen and phosphorus co-doped CDs with a high PL QY of 43.2%, prepared from the hydrothermal treatment of adenosine 5′-triphosphate as the carbon, nitrogen, and phosphorus source. The as-synthesized CDs exhibited fluorescence quenching in the presence of Fe^3+^ ions with a wide linear range of 1–150 µM and LOD of 0.33 µM. The accuracy of the proposed nanoprobes was further evaluated in river water samples and ferrous sulfate tablets. Furthermore, the CDs were employed for direct sensing of Fe^3+^ ions in biological samples such as human blood serum and living cells. The fluorescence quenching was attributed to static quenching arising from the formation of Fe–O–P bonds, as confirmed by fluorescence lifetime analysis, which showed a nearly consistent fluorescence lifetime of 5.87–5.85 ns before and after the addition of Fe^3+^ ions.

**Table 2:** Recent advances in the preparation of CDs, their corresponding properties, and Fe^3+^ ion sensing performance

Precursors	Synthesis route	QY (%)	Linear range	LOD	References
Chemical precursor					
Citric acid and guanidinium	Solid-phase pyrolysis	19.2	0–200 µM	100 nM	[114]
2,5-diaminobenzenesulfonic acid and 4-aminophenylboronic acid hydrochloride	Hydrothermal	5.44	0.3–546 µM	90 nM	[115]
Folic acid, 3-aminopropyl trimethoxy silane, glycerol	Hydrothermal	46	10 nM–45 µM	3.8 nM	[116]
Citric acid, diammonium hydrogen phosphate	Hydrothermal	59	20–200 µM	20 µM	[117]
Adenosine 5′triphosphate	Hydrothermal	43.2	1–150 µM	0.33 µM	[112]
l-glutamic acid	Solid-phase microwave-assisted pyrolysis	41.2	–	10^−5 ^M	[118]
dl-malic acid, ethanolamine, ethane-sulfonic acid	Microwave-assisted pyrolysis	15.13	6–200 µM	0.8 µM	[119]
Chitosan, acetic acid and 1,2-ethylenediamine	Microwave-assisted pyrolysis	20.10	0.01–1.8 ppm	10 ppb	[111]
Anhydrous citric acid, 1–10 phenanthroline	Pyrolysis	10	0–50 µM	35 nM	[120]
Aminosalicylic acid	Solvothermal	16.4	1–250 µM	0.52 µM	[121]
Green precursors					
Mangosteen pulp	Pyrolysis	–	0–0.18 mM	52 nM	[122]
Coffee ground	Hydrothermal	5	–	9 nM	[123]
Jujubes	Hydrothermal	–	0–200 µM	–	[124]
Sweet potatoes	Hydrothermal	8.64	1–100 µM	0.32 µM	[125]
Jinhua bergamot	Hydrothermal	50.78	0.025–100 µM	0.075 µM	[38]
Rose-heard radish	Hydrothermal	13.6	0.02–40 µM	0.13 µM	[113]

In addition to chemical precursors, CDs prepared from green precursors have also been employed as fluorescent nanoprobes for ferric ion detection. For instant, Liu et al. [113] prepared CDs via a one-pot hydrothermal route from rose heart radish for the detection of ferric ions. The LOD was estimated to be 0.13 µM with a linear range of 0.02–40 µM. Similarly, the fluorescence quenching was attributed to the specific interactions between Fe^3+^ ions and the phenolic hydroxyl groups on the edge of CDs. The excited electrons of CDs would transfer to the unfilled orbital of Fe^3+^ ions, leading to non-radiative electron–hole recombination and fluorescence quenching. Aslandas developed a green synthesis route to prepare CDs without heat treatment [126]. Briefly, blueberries were powdered with the assistance of liquid nitrogen before stirring at 8500 rpm for 30 min and sonication for 3 h. The limpid supernatant was separated and used for Fe^3+^ ion sensing with an LOD of 9.97 µM and a linear range of 12.5–100 µM. A PET process was confirmed as the quenching mechanism by reduction in the fluorescence lifetime from 4.51 to 3.8 ns. Many other groups have also reported the detection of ferric ions using CDs prepared from green precursors such as cotton, coriander leaves, sweet potatoes, garlic, onion waste, lemon peel, and fruit extracts [127–131].

**Fig. 15:**
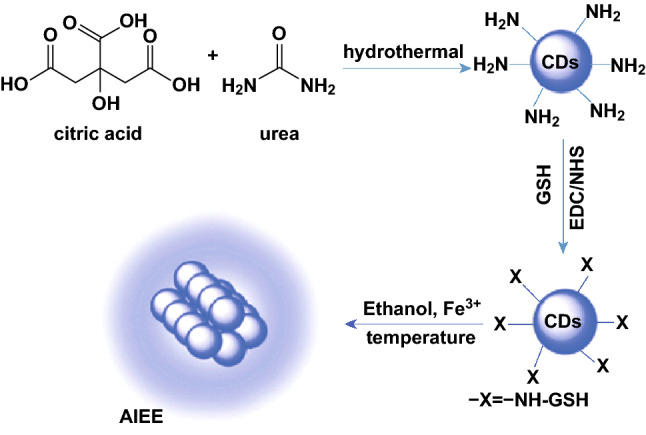
Preparation of CDs by hydrothermal treatment of CA and urea and sensing based on aggregation-induced emission enhancement effects [132]. Copyright © 2016 The Royal Society of Chemistry

In addition to fluorescence quenching-based ferric ion detection, other detection mechanisms have been studied using CDs as the nanoprobes. For instance, Wang et al. [132] developed a novel “turn-on” sensor for the detection of Fe^3+^ based on CDs with aggregation-induced emission enhancement properties (Fig. 15). CDs were prepared through a hydrothermal route with citric acid as the carbon precursor and urea as the passivation agent. The abundance of amino groups on the surface of CDs favored the surface modification. Glutathione (GSH) was then conjugated on the CDs through a carbodiimide-activated coupling reaction, endowing CDs with abundant carboxyl and hydroxyl moieties on their surface, thus improving their solubility and stability. The introduction of GSH would also endow the CDs with aggregation-induced emission enhancement properties. Upon addition of Fe^3+^, the GSH-modified CDs aggregated, resulting in an increase in the PL intensity. The LOD was found to be 0.1 µM, which is lower than the maximum permissible level allowed in drinking water by the US Environmental Protection Agency (EPA). The GSH-modified CDs were also used as a turn-on probe to detect Fe^3+^ in cells and sense the temperature. In another report, Deng et al. [133] developed a FRET-based ratiometric ferric ion nanosensor. In this design, the CDs served as the energy donor and anchoring place for the rhodamine group. The rhodamine moiety, which served as the detection unit for Fe^3+^, also acted as the energy acceptor. After the addition of Fe^3+^, the intensity of the emission peak of the CDs (donor) at 455 nm decreased, while a new emission peak emerged at 550 nm. The color of the mixture also changed from colorless to pink, allowing the visual colorimetric detection of Fe^3+^. The changes in PL emission and colorimetric response originated from the rhodamine moieties, which changed into an energy acceptor upon addition of ferric ions due to the Fe^3+^-induced ring opening of rhodamine 6G, resulting in a FRET process. The LOD of the nanoprobe was estimated to be 7.27 × 10^−7^ M and was able to operate in the range from 0 to 50 µM of ferric ions.

Most groups have reported the detection of a single type of target ions or molecules; however, multifunctional CD-based nanoprobes are of great interest as they can detect different target molecules using the same platform. For instance, Yu et al. [38] developed the hydrothermal synthesis of water-soluble CDs from Jinhua bergamot for the detection of Fe^3+^ and Hg^2+^ ions. The selective detection of Fe^3+^ and Hg^2+^ was achieved using different buffer solutions. When HAc-NaAc was used as the buffer solution, the Hg^2+^ ions quenched the PL of the CDs. Conversely, Fe^3+^ quenched the CD PL when Tris–HCl was used. The influence of the assay buffer on the selective detection of Hg^2+^ and Fe^3+^ is the result of the CDs having higher affinity toward Tris–HCl than to Hg^2+^. The quenching of the CD fluorescence by these two metal ions was attributed to the strong binding affinity of these metals to the amino and carboxylic moieties on the outer layer of the CDs. In addition, the redox potential of Hg^2+^ and Fe^3+^ lies between the conduction band and valence band of the CDs, causing photo-induced electron transfer from the conduction band to the complex states of Hg^2+^ and Fe^3+^. Likewise, Chandra et al. [45] reported the detection of iodide and Fe^3+^ ions using a nitrogen and phosphorus co-doped CD assay. The LOD for iodide and Fe^3+^ were 0.32 µM and 72 nM, respectively. Song et al. [134] developed a microwave-assisted preparation of yellow fluorescent CDs from *o*-phenylenediamine (OPD) capable of the sensitive detection of Fe^3+^ and hydrogen peroxide.

### Copper Ions

Aside from iron ions, copper ions are the most abundant essential transition metal ions in the human body, responsible for the synthesis of many important enzymes including cytochrome c oxidase, tyrosinase, and dopamine beta hydroxylase, which are involved in vital physiological processes required for growth and development [135]. Copper ions can be divided into two types, organic copper, which originates from food, and inorganic copper, which is provided from drinking water and health supplements [136]. Organic copper is processed by the liver, which does not permit the excessive release of free copper into the blood. Contrarily, inorganic copper can bypass the liver and contribute immediately to the copper levels in blood. Excessive copper intake can lead to serious health complications. For instance, high copper intake is known to induce toxicity and may cause damage to the central nervous system, resulting in neurodegenerative diseases such as Alzheimer and Parkinson diseases [137]. The situation is worsened by environmental pollution caused by copper, to the extent that copper has been listed as one of the priority pollutants by the US EPA. In view of the adverse consequences of high copper intake, a simple and fast manner to quantitatively and selectively monitor copper ions, especially in the environment and drinking water, is highly desirable.

Liu et al. [138] prepared highly luminescent nitrogen and sulfur co-doped CDs capable of sensitively and selectively detecting Cu^2+^ using a one-step microwave heating method with citric acid, l-cysteine, and dextrin as the precursors. By measuring the extent of fluorescence quenching, the as-synthesized CDs were able to detect copper concentrations as low as 20 nM. Chen et al. [139] prepared multicolor fluorescent CDs through a solvothermal route with citric acid and urea as the carbon and nitrogen precursors, respectively, and dimethylformamide as the solvent. Interestingly, upon addition of copper ions, only the long-wavelength red emission of the CDs was quenched. This was due to the strong interaction of copper ions with the nitrogen-containing moieties on the surface of CDs responsible for the red emission. Efficient energy transfer from the nitrogen-containing functional groups to the cupric ion quenching centers resulted in a significant decrease in the red PL intensity. Emission at other wavelengths was not affected by the presence of cupric ions because the interactions with oxygen-containing functional groups (responsible for emission at shorter wavelengths) are weaker than those with nitrogen-containing functional groups, as well as the good energy state matching between the nitrogen-containing emitting states of CDs and cupric ions. The proposed CDs presented an LOD of 40 nM, much lower than the upper limit requirement of copper ions in drinking water set by the US EPA.

Many groups have also channeled their efforts toward the preparation of CDs from green materials, capable of detecting cupric ions without any surface functionalization. For instance, Gedda et al. [140] developed the synthesis of CDs from prawn shells that served as a fluorescence sensing probe for Cu^2+^ ions. The prawn shells were subjected to deproteinization with sodium hydroxide, followed by a demineralization process with hydrochloric acid to remove proteins and minerals contained in the prawn shells to produce pure chitin. Chitin was then transformed to chitosan via deacetylation by sodium hydroxide treatment and the resultant product was subjected to hydrothermal treatment to produce fluorescent CDs with a PL QY of 9%. The as-synthesized CDs were able to detect cupric ions with an LOD of 5 nM in drinking water, river water, and even in seawater, which contains large amounts of minerals. In addition to biomass, other green materials such as food products or waste products have also been explored as a potential precursor for the preparation of CDs capable of selectively and sensitively detecting the presence of Cu^2+^ ions. Liu et al. developed a hydrothermal method to prepare CDs using pear juice as the precursor. The fluorescence of the resultant CDs was quenched by cupric ions in the linear concentration range of 0.1–50 mg L^−1^ [141]. Kumari et al. [142] investigated the synthesis of CDs from the pyrolysis residue of waste polyolefins. The as-prepared CDs were capable of detecting cupric ion concentrations as low as 6.33 nM with a linear detection range of 1–8.0 µM.

Despite the variety of methods and precursors explored to prepare fluorescent CDs, not all CDs respond to the presence of cupric ions. Additional functionalization with molecules can be carried out to endow CDs with the capability to detect cupric ions. Liu et al. [143] reported the synthesis of fluorescent CDs from bamboo leaves using a one-step hydrothermal method. The CDs were functionalized with branched polyethylenimine (BPEI) via electrostatic interactions, which served as a CD stabilizer for dispersion in water and various buffers, as well as binding site for cupric ions due to the abundance of amine groups. The LOD was reported to be 115 nM with a detection range from 0.333 to 66.6 µM. Fu et al. [144] functionalized CDs with thiosemicarbazide through amine bonds for the detection of copper ions. The attachment of thiosemicarbazide on CDs played a key role in cupric ion sensing as it provided abundant nitrogen-containing functional groups. It is well known that nitrogen atoms display high binding affinity toward copper ions. The functionalized CDs were able to complex the copper ions leading to fluorescence quenching due to IFE.

The presence of copper ions has also been reported to enhance the fluorescence of CDs. Vedamalai et al. [145] synthesized CDs from OPD using a hydrothermal method. The as-synthesized CDs were functionalized with OPD and, upon addition of Cu^2+^, Cu(OPD)_2_ complexes were formed inhibiting the photo-induced electron transfer and enhancing the PL intensity of CDs. The CDs were reported to present an LOD of 1.8 nM with a linear concentration range of 2–80 nM. Apart from functionalization with molecules during or post-synthesis, QDs have also been used in conjunction with CDs in a ratiometric copper ion sensor. Compared to single fluorescence quenching nanoprobes, ratiometric fluorescence probes are easier to evaluate by the naked eye. Also, the fluctuation of the excitation light intensity can be eliminated by measuring the intensity ratio of the two emission peaks. Rao et al. [146] developed a dual-emission nanoprobe using CdTe QDs modified with thioglycolic acid conjugated with amino-functionalized silica-coated CDs via carbodiimide chemistry. The red emission from the CdTe QDs was quenched in the presence of copper ions, while the blue fluorescence emission remained unaffected. The LOD was calculated to be 0.096 µM.

**Fig. 16:**
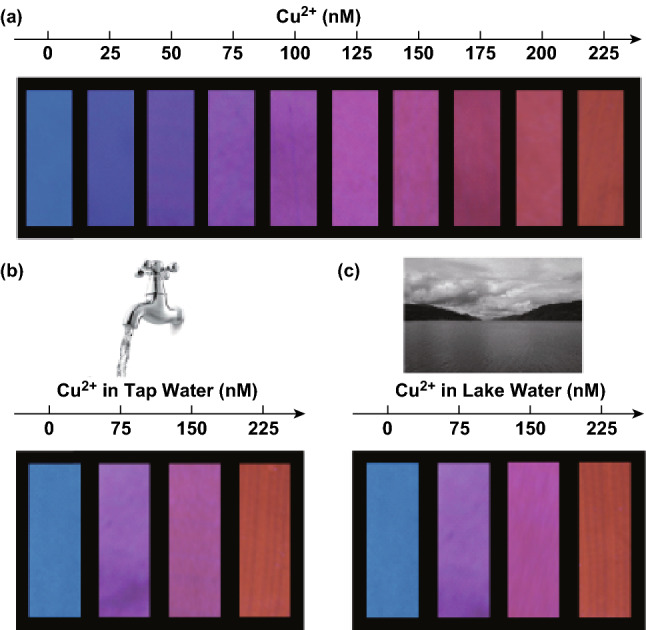
**a** Visual detection of Cu^2+^ ions using filter papers printed with dual-colored CDs, and visual detection of Cu^2+^ in **b** tap water and **c** lake water. All images were taken under 365 nm illumination [147]. Copyright © 2017 American Chemical Society. (Color figure online)

Most reported CD-based copper ion sensing assays are designed to be carried out in solution phase, requiring a fluorescence spectrometer for the detection of the target analytes. Solid-state sensors have the advantages of being portable, robust, durable, and easy to store. Liu et al. [147] developed a novel two-colored CD ratiometric fluorescence test paper for semi-quantitative copper ion detection. In this work, two different CDs with red (*r*-CDs) and blue (*b*-CDs) emission were prepared using solvothermal and hydrothermal methods, respectively. *r*-CDs and *b*-CDs were combined at a ratio of 1:7 and employed as fluorescent ink for jet-printing on filter papers (Fig. 16). The test papers were pale pink in color under ambient light and emit uniform blue fluorescence under 365 nm UV light irradiation. Upon exposure to increasing amounts of Cu^2+^ ions, the fluorescence emission of the test paper progressively and consecutively changed from blue to purple to pink to orange-red. The evolution of color was due to the fact that the blue fluorescence from the b-CDs was gradually quenched with the increasing Cu^2+^ concentration. Each discernable color, observable by the naked eye, could be correlated to a specific concentration of Cu^2+^ ions at intervals of 25 nM, thus allowing the semi-quantification of the amount of Cu^2+^ ions. Overall, Table 3 summarizes all CD-based Cu^2+^ ion sensors and their respective sensing performances [138–145, 148–154].

**Table 3:** Recent advances in the preparation of CDs, their corresponding properties, and Cu^2+^ ion sensing performance

Precursors	Synthesis route	QY (%)	Linear range	LOD	References
Chemical precursor					
Citric acid, urea	Solvothermal	39–43	1–10 µM	40 nM	[139]
Ionic liquids	Microwave-assisted pyrolysis	10.23–25.8	–	5 nM	[148]
Glucose and PEG-200	Microwave-assisted pyrolysis	–	2.0x10^−12^–1.5x10^−9^ mol mL^−1^	5.8x10^−13^ mol	[149]
*o*-phenylenediamine	Hydrothermal	2	2–80 nM	1.8 nM	[145]
Uric acid, phenylboronic acid	Hydrothermal	–	0.0033–80 µM	1.5 nM	[150]
Chitosan hydrogel	Microwave-assisted pyrolysis	–	–	0.5 μM	[151]
Aminophenylboronic acid	Thermal combustion	1.6	1–25 µM	0.3 µM	[152]
Ethylenediaminetetraacetic acid	Hydrothermal	–	0–0.4 µM	3.47 nM	[144]
Citric acid, l-cysteine, dextrin	Microwave-assisted pyrolysis	22	0–30 µM	20 nM	[138]
Green precursors					
Petroleum coke	Ultrasonic-assisted chemical oxidation	9.8	0.25–10 µM	0.0295 µM	[153]
Prawn shells	Hydrothermal	9	- 5 µM	5 nM	[140]
Vegetables	Hydrothermal	37.5	0–100 nM	9.98 nM	[154]
Pear juice	Hydrothermal	–	0.1 to 50.0 mg L^−1^	0.1 mg L^−1^	[141]
Waste polyolefins residue	Hydrothermal	4.84	1–8 µM	6.33 nM	[142]
Bamboo leaves	Hydrothermal	7.1	0.333 to 66.6 µM	115 nM	[143]

### Mercury Ions

In the 1950s, the Minamata Bay tragedy shocked the world with the dangers of mercury contamination. City dwellers around Minamata Bay experienced mercury poisoning with a detected level as high as 705 ppm in hair, resulting from the consumption of fish and shell fish contaminated by wastewater from a chemical plant [155]. Victims suffered from ataxia, numbness in hands and feet, muscle weakness, weakening of vision, and damage to hearing and speech; more severe cases involved insanity, paralysis, coma, and death of patients in a matter of weeks of the onset of symptoms. Many cases of congenital symptoms, such as intelligence disturbance, deformity of limbs, cerebellar symptoms, and dysarthria, also resulted from mercury poisoning [156]. It is known that mercury ions (Hg^2+^) can be absorbed by the human body through the skin, respiratory, and gastrointestinal tissues. In view of the severity of mercury poisoning, the US EPA has set a maximum allowable level of Hg^2+^ in drinking water of 10 nM. Tang et al. [157] developed a one-step hydrothermal synthesis of fluorescent CDs from glucose with polyamidoamine and (3-aminopropyl) triethoxysilane as the passivation agent. As a result of surface passivation, the CDs exhibited a high PL QY of 52.6% using quinine sulfate as the standard. Also, the surface-modified CDs presented abundant nitrous and oxygenated groups, which have high affinity toward Hg^2+^ forming stable chelate complexes. The CDs could detect as low as 0.087 nM of Hg^2+^ and operate in the ranges 0.2–15 nM and 500–10^4^ nM. Green materials such as pigeon feathers, eggs, and manure have also been reported to afford fluorescent CDs through facile pyrolysis carbonization methods, capable of detecting Hg^2+^ and Fe^3+^ ions with a low LOD of 10.3 and 60.9 nM, respectively [158].

Doping with heteroatoms is a way to create more active site on CDs, which may widen the applications of CDs for heavy metal sensing. Yuan et al. [159] designed germanium-doped CDs, prepared by a carbonation synthesis method and capable of detecting mercury ions at concentrations as low as 0.075 µM in the range of 0.2–3 µM. Interestingly, the CDs also exhibited excitation-independent PL characteristics, attributed to the uniform size and surface state of the CDs. In addition, the introduction of germanium in the CDs increased the PL QY from 5.6 to 8.9%. Zhang et al. [160] prepared lanthanum-doped CDs from disodium triphosphate (ATP) and LaCl_3_·2H_2_O using a solvothermal synthesis route. The as-synthesized CDs presented an LOD of 0.1 µM with a linear range from 0.5 to 40 µM. Li et al. [161] found that simultaneous doping of nitrogen and sulfur in CDs can effectively encourage the electron transfer and coordination interactions between the CDs and Hg^2+^ ions. In their report, the nitrogen and sulfur co-doped CDs served as a label-free Hg^2+^ fluorescence nanoprobe with an LOD of 2 µM. Their application could be extended to detect Hg^2+^ ions in lake water and tap water.

Functionalization of CDs with chelating agent is another route for mercuric ion detection. The additional functionalization steps can endow the CD-based probe with the capability to detect mercuric ions and further improve the sensitivity of the probe. For instance, Li et al. [162] prepared amine-functionalized CDs using a low-temperature treatment for the carbonization of citric acid and branched polyethylenimine. Thereafter, thymine moieties were conjugated on the surface of CDs through EDC/NHS coupling chemistry to acquire thymine-functionalized CDs. These CDs were able to detect Hg^2+^ ions in the linear range from 0 to 1 µM with an LOD of 3.5 × 10^−8^ M. Gupta et al. prepared a highly sensitive CD-based fluorescent probe by a two-step synthesis route. CDs were first prepared by microwave irradiation of a mixture containing chitosan gel and polyethylene glycol. Subsequently, the as-prepared CDs were functionalized with dithiothreitol for the detection of Hg^2+^ ions. Additional functionalization steps improved the lowest detectable limit from 6.8 nM to 18 pM. This was achieved via the introduction of thiol groups, which exhibit stronger affinity toward Hg^2+^ ions of the order from 10^15^ to 10^20^ compared to –NH_2_ and carbonyl groups.

**Fig. 17:**
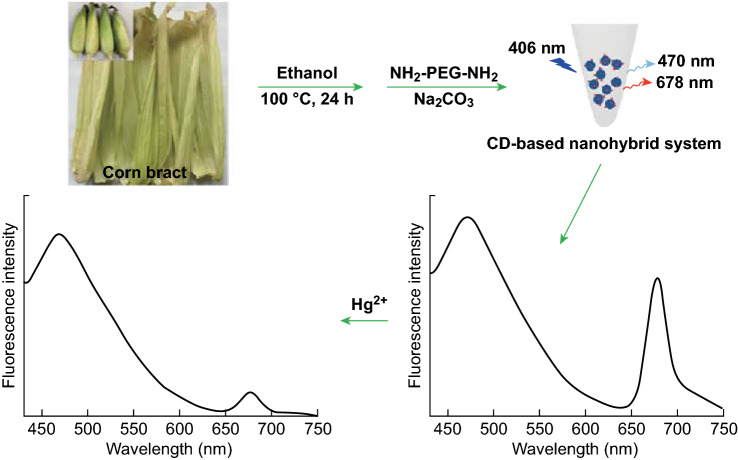
Schematic illustration of the preparation of dual-emission CDs and their application as ratiometric sensors for Hg^2+^ [165]. Copyright © 2017 American Chemical Society

Ratiometric CD-based mercury ion fluorescence probes can be achieved by conjugation with other types of QDs such as CdTe QDs. Xu et al. [163] covalently linked blue-emission CDs to the surface of silica nanoparticles containing red-emission CdTe QDs, which served as the constant reference signal. The blue-emission CDs bearing abundant hydroxyl and carboxyl functional groups were quenched in the presence of Hg^2+^. Hence, with the increasing concentration of Hg^2+^, the fluorescence of the CD hybrid probe experienced a color change from light purple to red, observable by the naked eye. Similarly, Liu et al. [164] proposed a ratiometric fluorescence sensor using a dual-emission CD-gold nanocluster functionalized with dithiothreitol. Owing to the strong metallophillic interactions between Hg^2+^ and Au^+^, the fluorescence intensity of the CDs at 466 nm remained constant, while the emission of gold nanoclusters at 598 nm was quenched. The resultant color change, from orange-red to blue, under UV light irradiation was obvious to the bare eye. The proposed fluorescence sensor exhibited an LOD of 8.7 nM. Zhao et al. [165] developed unique dual-emission CDs by solvothermal treatment of corn bract (Fig. 17). The as-prepared CDs presented dual-emission peaks at 470 and 678 nm, which originate from the intrinsic structure of the CDs and chlorophyll-derived porphyrins, respectively. The fluorescence at 678 nm was quenched by Hg^2+^, while the emission intensity at 470 nm was only partially affected. The fluorescence intensity ratio of both emission bands was linear in the Hg^2+^ concentration range from 0 to 40 µM with a low LOD of 9 nM. Table 4 summarizes some of the research works related to Hg^2+^ ion sensing using CD-based sensors [22, 157, 158, 160, 161, 165–177].

**Table 4:** Recent advances in the preparation of CDs, their corresponding properties, and Hg^2+^ ion sensing performance

Precursors	Synthesis route	QY (%)	Linear range	LOD	References
Sodium citrate, urea	Electrochemical carbonization	11.9	0.01–10 µM	3.3 nM	[22]
Citric acid, rubeanic acid	Microwave-assisted pyrolysis	17.6	0–20 µM	0.18 µM	[166]
Citric acid, urea, thiourea	Microwave-assisted pyrolysis	19.2	0.1–20 µM	62 nM	[167]
Chitosan gel, PEG	Microwave-assisted pyrolysis	13.4	0–2 × 10^4^ nM	10 pM	[168]
Citric acid/urea/l-cysteine	Microwave-assisted pyrolysis	25	0–40 µM	2 µM	[161]
Aconitic acid, ethylenediamine	Microwave-assisted pyrolysis	45.1	0–50 nM	5.5 nM	[169]
Citric acid, biuret	Microwave-assisted pyrolysis	15.3	2–22 µM	0.017 µM	[170]
Citric acid, diethylenetriamine	Thermal reflux	82.4	0–80 µM	0.201 µM	[171]
Tartaric acid, citric acid and ethanediamine	Solvothermal	42.2	0–18 µM	83.5 nM	[172]
Adenosine disodium triphosphate	Solvothermal	13.9	0.5–40 µM	0.1 µM	[160]
Sodium citrate, glutathione	Hydrothermal	21.03	0–15 µM	25 nM	[173]
Sodium citrate, histidine	Hydrothermal	29.7	0–40 µM	25 nM	[174]
Citric acid ethylenediamine	Hydrothermal	9.9	10 × 10^−9^–7 × 10^−7^ mol L^−1^	5.7 × 10^−10^ mol L^−1^	[175]
Glucose, polyamidoamine, 3-Aminopropyltriethoxysilane	Hydrothermal	52.6	0.2–15 nM	0.087 nM	[157]
Chitosan	Hydrothermal	31.8	0.25–6 µM, 80–300 µM	80 nM	[176]
Citric acid, ammonia	Hydrothermal	35.4	0–10 µM	1.48 nM	[177]
High-temperature-dried corn bract	Thermal pyrolysis	6.9	0–90 µM	4 µM	[165]
Biomass	Thermal pyrolysis	16.34–24.87	0–1.2 µM	10.3 nM	[158]

### Other Cations

Apart from ferric, mercuric, and cupric ions, fluorescent CDs have also been applied to detect a series of other metal ions, such as silver, chromium, gold, platinum, lead, palladium, potassium, cadmium, and zinc, through different detection mechanisms. For instance, Ahmed et al. [178] reported the application of CDs for the selective detection of silver ion via formation of silver nanoparticles. Upon addition of 25 µM Ag^+^, the color of the CDs changed to yellow, indicating the formation of silver nanoparticles, which was further confirmed by measuring the characteristic surface plasmon resonance at 410 nm. In addition, transmission electron microscopy images revealed the spherical morphology of the silver nanoparticles with sizes ranging from 10 to 15 nm, while the selected area diffraction pattern revealed their crystalline structure. The sensing mechanism was attributed to the capability of CDs for the reduction of Ag^+^ into silver nanoparticles, which resulted in the fluorescence emission changing from blue to cyan to green to colorless, accompanied by the quenching and a red-shift of the emission. Contrary to the popular fluorescence quenching mechanism, Gao et al. [179] reported the detection of Ag^+^ ions based on Ag^+^-induced fluorescence enhancement. The fluorescence enhancement originated from the reduction of Ag^+^ to Ag^0^ on the surface of the CDs, which enhanced the radiative emission of CDs. The proposed sensor presented an LOD of 320 nM and a linear operating range of 0–90 µM.

Similarly, many groups have reported the detection of other metals through different mechanisms with varied sensitivity. For instance, the detection of hexavalent chromium(VI) has been reported through different fluorescence quenching or enhancement mechanisms. Vaz et al. [180] explained that the quenching of fluorescent CDs in the presence of varying chromium(VI) concentrations in water and soil samples was due to IFE and static quenching mechanisms. The proposed sensor had an LOD of 0.03 µg mL^−1^ with a linear detection range of 0.1–12 µg mL^−1^. Shen et al. [181] alternatively presented the fluorescence quenching of their as-synthesized nitrogen and sulfur co-doped CDs by the antioxidation properties of chromium(VI). On the other hand, Liu et al. [182] attributed the CD fluorescence enhancement in the presence of chromium(VI) to the coordination between chromium(VI) with certain functional groups. The sensor in their work afforded an LOD of 0.21 µg L^−1^.

### Anions

It has been reviewed in the previous section that certain metals such as ferric and mercuric can quench the fluorescence of CDs by forming non-radiative complexes. The emission of CDs can be recovered by addition of certain anions that bind the metal cations, freeing the radiative pathway. Chen et al. [183] developed cyclam-functionalized CDs for the detection of copper and sulfide anions. Fluorescence energy transfer took place when copper ions were present on the surface of the cyclam-functionalized CDs, resulting in fluorescence quenching. Upon addition of sulfide ions to the quenched CD solution, these ions combine with the copper cations forming a stable complex and restoring the radiative path of the CDs. Quantitative analysis showed that their CDs had an LOD of 100 nM and 20 µM for copper and sulfide anions, respectively. Amin et al. [184] reported the selective detection of selenite ions based on an on–off–on CD nanoprobe. The fluorescence of CDs was quenched upon addition of europium ions due to the high affinity of the carboxylate and hydroxyl groups on the surface of CDs, leading to aggregation of the CDs. However, upon addition of selenite, in accordance with hard and soft (Lewis) acid and base theory, the selenite ions (hard base) strongly coordinated to the europium ions (hard acid), restoring the fluorescence. Such fluorescence recovery enabled the linear detection of selenite ions with an LOD of 54 ng mL^−1^. This on–off–on phenomena can be used to detect many different combinations of metals cations and anions, such as Hg^2+^–I^−^, Fe^3+^–F^−^, Ag^+^–S^−^, and Hg^2+^–S^−^. Other research groups have reported the detection of anions via surface modification or functionalization of CDs with specific chelating agents. For instance, Baruah et al. [185] functionalized CDs with β-cyclodextrin or calix [4] arene-25,26,27,28-tetrol for the detection of fluoride. Upon capping with the macromolecules, the fluorescence of CDs showed a decrease in the PL intensity. However, the PL intensity gradually increased with the increasing F^−^ concentration. The PL recovery was attributed to the presence of fluoride ions in the cavity of the functionalized CDs, which created a new and efficient radiative pathway.

### Biothiols

Compounds with thiol functional groups, such as homocysteine (Hcy), cysteine (Cys), and gluthathione (GSH), play vital roles in human physiology owing to their redox features. High levels of Hcy, also known as hyperhomocysteinemia, have been reported to result in endothelial cell damage, reduced elasticity of vessels, and changes in the hemostasis process. This abnormality has a significant correlation with cardiovascular disease and other critical complications, such as cerebral infarction, myocardial infection, and cardiovascular death [186]. On the other hand, Cys, an essential sulfur-containing amino acid, is involved in sensing and transducing changes in cellular redox states caused upon the generation of reactive oxygen species (ROS) and the presence of oxidized thiols. Elevated levels of Cys can cause oxidative DNA damage by promoting Fenton reactions, while a Cys deficiency is associated with lethargy, liver damage, and muscle and fat loss [187, 188]. GSH, a tripeptide containing amino acids glutamate, Cys, and glycine, is the most abundant and can be found in virtually every cell of the human body with the highest concentration in the liver. Virus infection, heavy metal intoxication, radiation, and even aging processes can cause free-radical damage to healthy cells and deplete GSH, which results in hampered immune function and increased susceptibility to infections [189, 190]. Hence, it is important to detect these biothiols in biosystems so as to diagnose and detect potential underlying illnesses.

Song et al. [191] developed a multifunctional green nanoprobe for GSH detection based on fluorescence quenching of nitrogen and sulfur co-doped CDs with an LOD of 6.7 µM and a linear range from 0 to 100 µM. In this work, the green fluorescence intensity of CDs decreased with the increasing concentration of GSH. The presence of other amino acids including Cys and Hcy in the mixture presented no significant interference on the selective detection of GSH, indicating that the nanoprobe had excellent selectivity toward GSH. The as-prepared CDs could also act as a multifunctional pH and temperature sensor. On the other hand, Guo designed a “turn-on” nitrogen and sulfur co-doped CD sensor capable of detecting Cys, GSH, and Hcy with an LOD as low as 86 nM [102]. The fluorescence emission peak at 481 nm was first quenched by cupric ions with half-filled d-orbitals, where electrons can be transferred from photoexcited CDs, resulting in non-radiative recombination. Upon addition of biothiols to the system, cupric ions are removed from CDs due to the binding preference of cupric ions for biothiols, followed by fluorescence recovery of the CDs.

To further improve the sensitivity and selectivity of the detection system, Wang et al. [192] prepared FRET-based CD-MnO_2_ nanosheets for glutathione sensing in human whole blood samples. CDs rich in carboxyl groups were first prepared using a facile hydrothermal route. Subsequently, the CDs were deposited on the surface of MnO_2_ nanosheets through electrostatic interactions, creating an effective FRET system where CDs serve as the donor and the MnO_2_ nanosheets as the acceptor. In the presence of GSH, the fluorescence of CDs was recovered. GSH is oxidized to glutathione disulfide by the MnO_2_ nanosheet, which is itself reduced to Mn^2+^, inhibiting the FRET process. The LOD was calculated to be 22 nM with a linear range from 0.2 to 600 µM; the sensor was capable of detecting GSH in human whole blood samples. Amjadi et al. [100] designed a FRET-based detection system using CDs as the donor and silver nanoparticles as the acceptor for the selective sensing of Cys. The system presented an LOD of 4 nM with a linear operating range of 6–300 nM.

Even though FRET has the capability of improving the sensitivity of sensing systems, it requires extensive design and selection of nanomaterials to act as the donor or acceptor. Hence, other sensing mechanisms such as IFE, which offers simplicity and flexibility, have been exploited for biothiol sensing. IFE is based on the absorption of emission light by absorbers in the sensing system, where the absorption spectra of the absorber overlaps with the fluorophore excitation spectra, emission spectra, or both. Wu et al. [193] developed IFE-based fluorometric sensing and imaging of GSH using CDs directly as the IFE fluorophore and 5,5′-dithiobis-(2-nitrobenzoic acid) (DTNB) as the recognition molecule for GSH. DTNB reacts with GSH producing 5-thio-2-nitrobenzoic acid (TNB), which is able to act as an IFE absorber owing to its high molar extinction coefficient. The group also confirmed that DTNB itself presents no interference on the fluorescence intensity of the CDs, while the reaction product of DNTB and GSH can quench the CD fluorescence on account of the good overlap between the absorption band of TNB and the excitation band of the CDs. The system operates linearly in a wide range from 0.2 to 1000 µM with a calculated LOD of 30 nM.

Deng et al. [194] developed a nanosensor comprised of CDs and AuNPs that is capable of selectively detecting cysteine with multiple signals, namely colorimetric, PL, and UCPL. The nanosensor was constructed by assembling CDs on the surface of AuNPs, forming an impact “shell” through Au–N interactions. This results in the aggregation of AuNPs due to the disordered assembly of AuNPs and CDs, which can be observed visually through the color change of the solution from red to purple. The PL and UCPL of CDs were also quenched by AuNPs, which was attributed to a FRET mechanism. Upon addition of Cys to the CD-AuNP mixture, the aggregated CD-AuNPs become disperse again with the color of the solution returning from purple to red. The desorption of CDs due to the competition between –SH and nitrogen-containing groups to the AuNPs restores the PL and UCPL of the CDs. This nanosensor has a reported LOD of 4 nM and a linear range from 0.01 to 2 µM.

### Reactive Oxygen and Reactive Nitrogen Species

The mitochondria is well known as the powerhouse of eukaryotes due to its most distinct function: the production of the energy “currency,” adenosine triphosphate (ATP), through respiration. In this process, electrons obtained from the digestion of carbohydrates and fats are sent to O_2_ via a chain of respiratory H^+^ pumps, creating many different types of ROS and reactive nitrogen species (RNS) such as ClO^−^, H_2_O_2_, ^1^O_2_, ONOO^−^, and NO [195]. In addition to ATP generation, the mitochondria also plays important roles in other metabolic reactions, such as steroid hormone and porphyrin synthesis, urea cycle, lipid metabolism, and interconversion of amino acids [196]. Also, the mitochondria are involved in ROS/RNS-induced apoptosis and mitophagy. Hence, oxidative stress to the mitochondria due to imbalance ROS/RNS generation and detoxification can lead to many neurogenerative diseases such as Alzheimer’s disease, ischemic stroke, ischemia–reperfusion, atherosclerosis, and cancer. Owning to these serious complications, it is vital to develop the selective and sensitive detection of ROS/RNS species.

Gong et al. [197] developed phosphorus and nitrogen co-doped CDs that can be used to detect multiple ROS and RNS intracellularly. The CDs exhibit blue fluorescence with a PL QY of 23.5% that can be selectively quenched by ClO^−^, ONOO^−^, and NO. The fluorescence peak of CDs at 406 nm gradually decreased with the increasing concentration of ROS/RNS. This system reported an LOD of 0.28, 0.38, and 1.1 µM for ClO^−^, ONOO^−^, and NO, respectively. The fluorescence quenching was attributed to the oxidation of CDs after addition of reactive species, resulting in changes in the surface properties of the CDs. This was further confirmed by the reduction in the decay times from 4.76 to 0.8 ns. In the work by Simões et al. [198], the synthesis parameters were varied using a multivariate full factorial experimental design methodology to produce CDs that were able to detect specific ROS/RNS. The optimal synthesis parameters were found to be a precursor solution consisting of 2.5 g citric acid and 1000 µL ethylenediamine in water under 5 min microwave irradiation to produce CDs capable of detecting NO with a 4.6 µM LOD. On the other hand, CDs capable of detecting ONOO^−^ were prepared under similar microwave irradiation conditions by modifying the precursor loading to 0.25 g citric acid and 500 µL ethylenediamine. This sensory system could detect ONOO^−^ concentrations as low as 2.7 and 2 µM at pH 7 and pH 10, respectively.

**Fig. 18:**
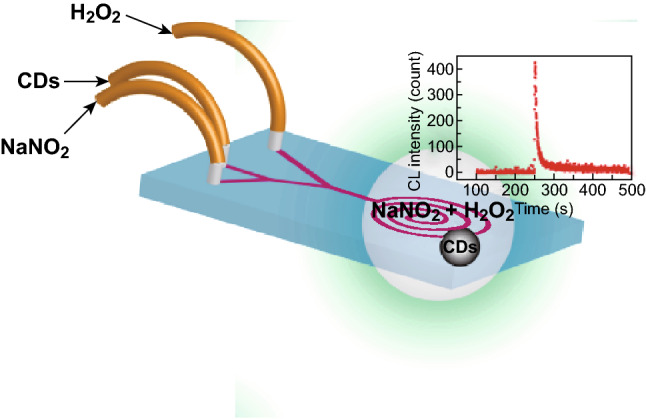
Schematic design of a microfluidic chip for peroxynitrous-acid-induced CL analysis [199]. Copyright © 2016 Elsevier B.V.

Apart from the usual fluorescence quenching mechanism, chemiluminescence (CL) is another phenomenon that can be used for the detection of ROS/RNS. CL refers to light emission originated from the energy released from certain chemical reactions. Lin et al. demonstrated the detection of peroxynitrous acid (ONOOH) by measuring the CL intensity of CDs. ONOOH, which is a weak acid with a p*K*a of 6.8, is converted into ONOO^−^-related species under basic conditions. The interaction of ONOO^−^ species with CDs results in enhancement of the CD CL in alkaline media. This sensing system has a sensitivity of up to 5 × 10^−9^ M and can be used to detect nitrate in water with a recovery rate of 98%. This system was further developed by Wu et al. [199] by introducing a microfluidic chip for peroxynitrous acid-induced CL for nitrite detection (Fig. 18). CDs and NaNO_2_ were premixed in the microchannels before reaction with acidified hydrogen peroxide in the spiral microchannels. The concentration and flow rate were optimized to achieve stronger CL signals. This approach was able to detect up to 1 × 10^−5^ M of nitrite and used for nitrite detection in water and beverages.

H_2_O_2_ is another highly reactive oxidant commonly detected using CD-based nanoprobes. H_2_O_2_ is widely used in industrial processes, water treatment, and disinfectants, and commonly found in many biochemical and medicinal reactions. Studies have shown that concentrations of H_2_O_2_ exceeding 0.7 µM pose a significant threat to living cells [200]. Abnormal levels of H_2_O_2_ in the human body can lead to several diseases such as dementia and even cancer. Lan et al. developed a turn-on fluorescence sensor that was capable of detecting H_2_O_2_ with an LOD of 84 nM. CDs were functionalized with diphenylphosphine moieties, where the former served as the PET donor and the latter the PET acceptor. The fluorescence of CDs was quenched after functionalization. However, in the presence of H_2_O_2_, diphenylphosphine would react with H_2_O_2_ to form diphenylphosphine oxide, obstructing the PET process and restoring the fluorescence of CDs. In addition to excellent fluorescence properties, CDs may also exhibit electrocatalytic capability, which may be exploited for sensing purposes. Su et al. employed an amperometric approach for the detection of H_2_O_2_. CDs were first prepared using a one-step hydrothermal process with silver nitrate and chitosan as the precursors [201]. Horseradish peroxidase was immobilized on the CDs before being placed on a glassy carbon electrode. The current response of the system exhibited a step-like increase with the addition of H_2_O_2_, reaching a steady-state current within 5 s. The system showed a linear response current at H_2_O_2_ concentrations in the range from 0.005 to 0.59 µM with an LOD of 0.0018 µM.

### Deoxyribonucleic Acid (DNA)

A CD-based ratiometric fluorescence nanosensor using an “on–off–on” fluorescence change scheme for DNA sensing was developed by Huang et al. [202]. Briefly, ethidium bromide (EB) with red emission was added to blue fluorescence-emitting CDs. The electron transfer process between EB and CDs effectively quenched the blue fluorescence, resulting in a fluorescence color change from blue to dark red, followed by enhancement of the EB fluorescence (bright red) upon introducing DNA into the mixture due to the strong intercalation of EB in DNA. This approach is advantageous as covalent immobilization of the probe molecule is not required, yet a broad dynamic linear range can be achieved, ranging from 1.0 to 100 μM with an LOD of 0.47 μM. Moreover, the sensor also demonstrated good selectivity (relative error below ± 5%) for the determination of 10 μM of DNA, even in the presence of coexisting substances such as trysin, lysozyme, bovine serum albumin, human serum albumin, and RNA. In the following year, Loo et al. [203] employed two different carboxylic CDs, derived from citric acid and malic acid, respectively, as nanoquenchers for DNA detection. This study presented two interesting findings, where fluorescence quenching of the carboxylic CDs occurred upon adsorption of the fluorescence-labeled single-strand DNA probe, while double-strand DNA formed from the hybridization of the fluorescence-labeled single-strand DNA probe and its complementary DNA did not react with the carboxylic CDs and the fluorescence was retained. The LOD of malic acid CDs was found to be 17.4 nM, better than that of citric acid CDs (45.6 nM) due to the large number of carboxylic groups in the former. Recently, Guo et al. proposed the application of positively charged, fluorescent N, S-co-doped CDs for DNA sensing through hybridization with complementary DNA sequences. The CDs acted as the nanoquencher toward the fluorophore-modified DNA while maintaining the intrinsic PL of the CDs, which served as an intrinsic reference signal for the ratiometric detection of DNA. Hybridization between the fluorophore-modified DNA and its complementary counterpart would lead to detachment of DNA from the CDs, thus restoring the fluorescence [204]. Despite the remarkable sensitivity and selectivity of these reported CD probe sensors for DNA detection, their capability to distinguish DNA targets and sequencing DNA is yet to be explored.

Another interesting CD-based DNA probe sensor was developed by Pang et al. [205] for the specific sensing of guanine, a primary nucleobase in DNA. The detection principle is unique in that the fluorescence of CDs is first quenched upon addition of copper ions, Cu^2+^, followed by fluorescence recovery when guanine is introduced in the CD-Cu^2+^ system. The work claimed a simple, facile, and one-step microwave-assisted preparation method, yet the sensor displayed a LOD of 0.67 × 10^−8^ mol L^−1^, much lower than those reported in other published works [206]. Moreover, the CD-Cu^2+^-based sensor evidently showed high selectivity toward guanine compared to other bases such as adenine, uracil, cytosine, and thymine. Nonetheless, the sensor was found to perform at its best under optimal conditions of neutral pH, boiling water temperature, and 8.0 × 10^−6^ mol L^−1^ of Cu^2+^. Under such conditions, the fluorescence intensity of CD-Cu^2+^-guanine was stable for at least 3 h at room temperature.

### Biomarkers

Biomarker sensing is a useful tool for early diagnosis of severe diseases. A wide variety of CD-based sensors using various sensing mechanisms have been reported thus far with great sensing capability that accurately meets the requirements to serve its purpose. For instance, Huang et al. [207] reported that hemoglobin (HGB) causes static quenching of CD fluorescence, unveiling the applicability of CDs for direct determination of the HGB marker associated with clinical diseases such as leukemia, and anemia. The developed CD fluorescence probe successfully achieved an LOD of 0.12 nM under controlled experimental conditions (i.e., pH 7.5 without NaCl in the salt solution) with a reaction time of just 1 min. Moreover, the work employed real human samples to evaluate the selectivity of the probe in the presence of other interfering chemical substances such as proteins, and amino acids. It was found that the effect of these interfering substances was negligible (relative error below ± 5%) toward the detection of 1.0 μM HGB. In contrast, a study conducted by Barati et al. [208] claimed that the quenching mechanism of their CD fluorescence probe in regard to HGB detection was based on IFE rather than static quenching. The developed CD fluorescence probe showed an LOD of 34 nM, which could be increased by 85-fold when H_2_O_2_ was intentionally added to the reaction. Barati et al. explained that such observation was attributed to the generation of hydroxyl radicals acting as strong oxidizing agents, resulting in high fluorescence quenching.

Dipicolinic acid (DPA) sensors for the diagnosis of anthrax disease caused by *Bacillus anthracis* have drawn much attention in the research field of CD-based sensing as this bacteria can be potentially used as a biological warfare agent [209]. Chen et al. [210] developed Tb^3+^-functionalized carbon dots as a nanoprobe sensor based on a ratiometric fluorescence sensing scheme. The weak blue luminescence of the CD-Tb^3+^ nanoprobe visibly changed to green emission upon reaction with a small amount of DPA of concentrations as low as 5 nM, much lower than the required infectious dosage of 60 μM. The reason behind such phenomenon was attributed to efficient energy transfer from DPA ligands to Tb^3+^, which resulted in two sharp peaks at 490 and 544 nm. In fact, the emission color change was detectable by the naked eye at 50 μM of DPA. The study also validated other attractive merits of the CD-Tb^3+^ nanoprobe sensor, such as good aqueous and photostability, broad operating pH range (3.5–10.5), good linearity over the nanomolar detection range, and insignificant interference from various interfering acids, metal cations, and anions. A comparable study performed by Rong et al. [211] demonstrated the viability of fabricating a color test strip using functionalized manganese-doped CDs for DPA sensing. The Mn-CDs were functionalized with ethylenediamine tetraacetic acid disodium salt and europium (Eu^3+^). In order to realize the detection, the functionalized Mn-CDs were excited at 270 nm, generating blue emission (461 nm) by default. However, upon addition of DPA, bright red fluorescence at 593 and 616 nm was observed. Since DPA presents an absorption band at 270 nm, it was deduced that DPA behaves as an antenna group collating the excitation light, followed by energy transfer to Eu^3+^. In short, the sensing principle of the sensor relies on absorption energy transfer emission from DPA to Eu^3+^ over the functionalized Mn-CDs. This CD-based test strip sensor showed good linearity in the range from 1.0 to 200 μM, with an LOD of 1.0 μM. Overall, the conclusions from these studies suggest that the advantages of CD-based sensors warrant their application as new nanosensing platforms since they have clearly overcome the limitations of traditional lanthanide ion-based sensing platforms, which suffer from poor stability due to a myriad of environmental and stability factors [212].

In recent years, glucose marker sensors have been the top search subject matter in the vast majority of the sensing field. Although the typical measured blood glucose levels are not low (one to tens of mmol L^−1^), an ultra-sensitive sensor is desirable to allow sample dilution for assay preparation in order to reduce potential interferences from the complex matrices of real samples [213]. Boronic acid, which bears a benzene ring, is commonly employed as binding site for glucose markers. Shen and Xia [214] fabricated boronic acid-functionalized CDs for nonenzymatic blood glucose detection. The authors selected phenylboronic acid as the sole precursor to prepare functionalized CDs via a one-step synthesis–modification approach through a hydrothermal carbonization process. Unlike other previously reported sensors, this boronic acid-functionalized CD sensor is advantageous in that the detection scheme does not involve expensive enzymes and tedious fabrication procedures. On top of this, the sensor shows good linearity in the range of 9–900 μM under basic conditions, which favors the formation of boronate complexes resulting from covalent binding between the *cis*-diols of glucose and boronic acid. As a downside, the study revealed that the interactions of glucose and the sensor reach equilibrium after 1 h of reaction, which is a rather long time, although it was claimed to be much shorter (by three times) than that of other AuNP-based sensors. While discrete detection of blood glucose levels may be able to meet the needs of most diabetic patients, this might not be sufficient for patients who are suffering from severe diabetes. Biocompatible CDs embedded in glucose-imprinted polymer microgels developed by Wang et al. [215] were responsive toward variations in the surrounding glucose levels, enabling the continuous monitoring of glucose at the localized site. This is feasible due to the capability of the microgel to swell and shrink (and vice versa) with changes in the glucose concentration. As in the case of Shen and Xia’s work [214], it was found that the swelling effect was attributed to the negatively charged boronate complexes that build up the Donnan potential, causing the microgels to swell. Aside from the proven negligible toxicity from 25 to 100 μg mL^−1^, the CDs immobilized in glucose-imprinted polymer networks exhibited a long luminescence lifetime of 71.96 ns, further reducing any potential interference from neighboring biological molecules with shorter fluorescent lifetimes. The nanoprobe sensor demonstrates good detection sensitivity over the clinical range of 0–30 mM at physiological pH, and the recovery of the fluorescence signal was stable even after five cycles of independent glucose detection.

Cancer tumor markers are the common target of many QD-based sensors for bio-applications. The determination of these markers has great importance for early diagnosis, disease condition monitoring, and recurrence prediction. Miao et al. [216] described a label-free CD-based carcinoembryonic antigen (CEA) sensor synthesized from tomato juice prepared via an environmentally friendly synthesis procedure. In principle, the CEA-aptamer was bound to the CD surface via π–π stacking interactions, leading to fluorescence quenching. However, the presence of CEA induces the detachment of the CEA-aptamer from the CD surface due to its strong binding affinity, resulting in fluorescence recovery and the possibility to quantitate the CEA concentration. The detection range showed good linearity from 1 ng mL^−1^ to 500 μg mL^−1^ with an LOD of 0.3 ng mL^−1^. The same underlying detection concept was implemented by Motaghi et al. [217], who selected AS1411, a nucleolin aptamer, to detect cancer cells. The sensitivity of the AS1411-CD sensor was found to be 4T1 < MCF7 < HeLa cells, suggesting that HeLa cells have more nucleolin on the surface than 4T1 and MCF7 cells. Excitation of the AS1411-CD sensor at 400 nm induced emission at 470 nm for a broad range of cell concentrations (10–4500 4T1 cells) with an LOD of 105 cell mL^−1^.

Alongside the development of proteomic and enzyme technologies, many protein and enzyme-based biomarkers have been discovered for many types of diseases. For instance, abnormal alkaline phosphatase (ALP) levels in human serum are often associated with various diseases, such as liver dysfunction and breast and prostatic cancer. Qian et al. reported dual-purpose carbon QDs for pyrophosphate anion (PPi) detection and ALP monitoring. Both analytes are correlated such that PPi can be hydrolyzed by ALP, being crucial for skeletal mineralization and vascular calcification. Hence, indirect analysis of ALP using PPi as the natural intermediate is promising [218]. In brief, the off–on–off sensing scheme was realized through the aggregation and disaggregation of CDs with copper ions. Copper was chosen for its strong quenching ability to allow easy recognition that aggregation and disaggregation had taken place. First, fluorescence quenching induced by the formation of CD-Cu complexes is recovered when PPi is introduced in the CD-Cu system due to the higher affinity toward Cu-PPi complex formation, affording free CDs. Further, when ALP is added to the system, the hydrolysis of PPi by ALP causes the copper ions to leave the PPi-Cu complex and aggregate with the CDs again, leading to a second cycle of fluorescence quenching. The study showed a broad detection range from 16.7 to 782.6 U L^−1^ and an LOD of 1.1 U L^−1^. Other green synthesized carbon dots using *Sterculia lychnophora* seeds have been reported for the detection of ALP by Qu et al. [101]. ALP was detected through CD fluorescence recovery when ascorbic acid-2-phosphate (AAP) was added to the system. ALP can bio-catalyze the AAP hydrolysis into ascorbic acid, further reducing manganese oxide to manganese ions and subsequently restoring the fluorescence of the CDs. The sensor exhibited an LOD of 0.4 U L^−1^ and good selectivity to ALP with negligible fluorescence changes when reacted with other control proteins. In addition to ALP, other CD-based sensors have been reported for the detection of protein and enzyme biomarkers such as hyaluronidase, acetylcholinesterase, and uric acid [219–221].

Dopamine (DA) is another biomarker of pivoting importance that can be detected using CD-based sensors. DA is a neurotransmitter in the brain released by nerve cells to regulate a wide range of functions, such as locomotion, emotion, learning, and neuroendocrine modulation [222]. Despite its simple molecular structure, an imbalanced level of dopamine can result in Parkinson’s disease and depression [223]. The detection of DA requires high sensitivity due to its presence in nanomolar concentration and high selectivity due to the coexistence of interfering species such as ascorbic acid, which is found in bodily fluids [224]. Li et al. [225] developed CDs using electrolysis synthesis methods capable of detecting dopamine with an LOD of 6 × 10^−8^ mol L^−1^. The CDs with a PL QY of 38% were functionalized with tyrosinase (TYR) to form a CD/TYR hybrid. TYR can oxidize dopamine to form dopamine quinone, which can be subsequently oxidized into dopaminechrome. During this process, the PL of the CD/TYR hybrid is quenched and the amount of quenching can be correlated with the concentration of dopamine present. This sensor system was further tested with human urine samples and the results were comparable to the high-performance liquid chromatography and fluorescence detection methods used in clinical settings. In a later report, the same group developed a low-temperature synthesis method to prepare nitrogen-doped CDs capable of detecting catechol and catechol derivatives [226]. C_3_N_4_ was added to ethane diamine and the mixture was refluxed at 80 °C for 12 h. The achieved LOD for dopamine, levodopa, carbidopa, and epinephrine were reported to be 61.2, 153, 86.2, and 215 nmol L^−1^, respectively. Overall, the merits of CD-based biomarker sensors such as ease of fabrication, green production, biocompatibility, and high sensitivity and selectivity demonstrate their advantages for scalability and practicality in bio-sensing applications.

### Bacteria and Cells

Wang et al. [227] developed a green synthesis approach using papaya powder as a natural carbon precursor capable of detecting both Fe^3+^ and *Escherichia coli* (*E. coli*). Aqueously prepared CDs experienced fluorescence emission enhancement in the presence of *E. coli* as compared to CDs prepared in ethanol, which exhibited no obvious response. This is due to the fact that CDs produced in aqueous medium containing saccharides such as mannose, which tend to present strong interactions with the FimH proteins available on the tip of the fimbriae of *E. coli*. A linear range of 10^5^–10^8^ cfu mL^−1^ was obtained with an LOD of 9.5 × 10^4^ cfu mL^−1^. Lai et al. [228] developed fluorescent CDs capable of detecting *E. coli* and tumor cells. CDs were first prepared by carbonizing ammonium citrate through solid-state dry heating before heating up with mannose or folic acid to produce mannose-functionalized CDs and folic acid-functionalized CDs. Both types of functionalized CDs exhibited emission peaks at 450 nm under 365 nm excitation. The mannose-functionalized CDs were applied for selective labeling of *E. coli* with an LOD as low as 100 cfu mL^−1^ in samples such as drinking water, juices, and urine. Other bacteria such as *Pseudomonas aeruginosa*, *Staphylococcus aureus*, and *Salmonella enteritidis* exhibited a negative response. On the other hand, folic acid-functionalized CDs displayed improved uptake compared to bare CDs and can thus be used to target tumor cells over-expressing the folate receptor (FR).

Duan et al. [229] developed a dual-FRET-based system capable of performing the simultaneous detection of two different types of pathogenic bacteria: *Vibrio parahaemolyticus* and *Salmonella typhimurium.* The system consists of green-emitting QDs (gQDs) and red-emitting QDs (rQDs), which serve as the donors, and amorphous carbon nanoparticles that serve as the acceptor. gQDs were functionalized with aptamers targeting *Vibrio parahaemolyticus*, while rQDs were functionalized with aptamers recognizing *Salmonella typhimurium.* The emission of both types of QDs was quenched in the presence of carbon nanoparticles. Upon addition of the target analytes, a complex is formed with the QDs and the quenching caused by carbon nanoparticles is suppressed. This system can detect these two pathogens in the range of 50–10^6^ cfu mL^−1^ with LODs as low as 25 cfu mL^−1^ for *Vibrio parahaemolyticus* and 35 cfu mL^−1^ for *Salmonella typhimurium*, which can be applied for detection in real food samples.

**Fig. 19:**
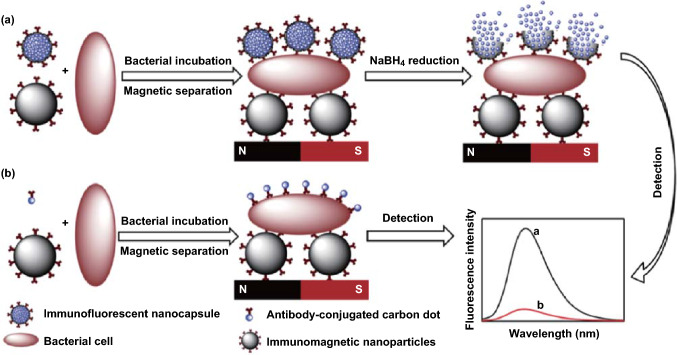
Schematic illustration of the detection of pathogenic bacteria with **a** CD-based nanoprobes and **b** conventional methods [230]. Copyright © 2018 American Chemical Society

Despite the advances on the use of CD-based fluorescent probes to detect pathogenic bacteria, detection at the single-cell level remains a challenge. Yang et al. [230] developed CD-encapsulated breakable organosilica nanocapsules (BONs) able to detect pathogenic bacteria at the single-cell level (Fig. 19). In their work, hundreds of CDs were encapsulated in each organosilica nanocapsule and the CD@BONs were further modified with anti-*Staphylococcus aureus* antibodies to recognize the target analyte through antibody–antigen interactions. Another type of immunomagnetic particles were also functionalized such that they were able to target *Staphylococcus aureus* bacteria. Both CD@BONs and the immunomagnetic nanoparticles were used simultaneously to detect *Staphylococcus aureus*. Subsequently, the targeted cells were magnetically separated and further reduced by sodium borohydride, releasing the CDs from the BONs and fluorescence measurements were carried out. This method of detection delivered enhanced sensitivity two orders of magnitude higher than that of the conventional method due to the improved fluorescence signal from the large number of CDs encapsulated in each nanocapsule. This system displayed good selectivity for *Staphylococcus aureus* when tested with other types of bacteria such as *E. coli, Salmonella*, and *Listeria monocytogenes.*

CDs have also been functionalized with specific ligands to selectively differentiate cancerous cells from normal cells. Among the targeting ligands, folic is a well-known low molecular weight vitamin that is vital for cell survival and has high binding affinity for the FR. FR is a type of cancer biomarker that is upregulated in cancerous cells but minimally distributed in normal cells. Hence, many groups have exploited these characteristics to visually identify cancer cells. For instance, Zhang et al. [231] prepared green fluorescent CDs by microwave irradiation of commercially available active dry yeast with a high yield of ~ 50%. Folic acid (FA) was further functionalized on CDs through EDC/NHS coupling and the resultant FA-CDs displayed excellent stability, low toxicity, and biocompatibility. The FA-CDs could penetrate the FR-positive HepG2 liver cancer cell membrane through receptor-mediated endocytosis. To further demonstrate the capability to distinguish cancerous cells from normal cells, a mixture of HepG2 cells and FR-negative PC12 cells was subjected to FA-CD labeling. It was found that HepG2 cells exhibit bright fluorescence intensity while PC12 cells do not. The difference in fluorescence intensity demonstrates that FA-CDs can differentiate FR-positive and FR-negative cells. On the other hand, Gao et al. [232] prepared label-free CDs from glycerol and (3-aminopropyl) trimethoxysilane capable of distinguishing cancerous cells such as MDA-MB-231, HeLa, Hep G2, U14, MCF-7, and A549 from normal cells.

### Nitroaromatic Explosives

Nitroaromatic compounds such as 2,4,6-trinitrophenol (TNP), 2,4,6-trinitrotoluene (TNT), and 1,3,5-trinitro-1,3,5-triazacyclohexane (RDX) are widely used in industries such as pharmaceutical, military, dye production, and leather and glass manufacturing. Despite their widespread applications, these compounds are renowned for being explosive and toxic and are a major environmental pollutant due to their relatively high water solubility. These pollutants enter the food chain and put the human health at risk, causing neurological diseases, abdominal pain, headaches, and even cancer in more severe cases. In view of the dangers caused by nitroaromatic compounds, Chen et al. [233] developed novel terbium-doped CDs for the detection of TNP. In their report, CDs were prepared through a facile carbonization process using a round-bottom flask containing citric acid and terbium(III) nitrate pentahydrate submerged in a thermostated oil bath at 190 °C for 30 min. The fluorescence of the as-synthesized CDs was quenched by TNP, rendering it a colorimetric assay for TNP detection. The group also confirmed the quenching mechanism through the investigation of the fluorescence lifetime energy levels of the doped CDs and TNP and the absorption spectra, which indicated that IFE and energy transfer were responsible for the fluorescence quenching. The proposed sensor provided a detection range from 500 nM to 100 µM with an LOD of 200 nM in complex water samples. In another report, Cheng et al. [234] developed the green synthesis of hydrophobic CDs capable of detecting TNP in hydrophobic media. The group also developed a qualitative test strip able of detecting TNP without the need for any laboratory equipment. The group attributed the fluorescence quenching of CDs in the presence of TNP to IFE. More recently, Ju et al. [235] investigated the quenching mechanism of CDs in the presence of TNP. The group proposed that the fluorescence quenching of CDs after the addition of TNP is attributed to electron transfer processes, which include hydrogen-bonding-assisted electron transfer and proton-assisted electron transfer.

Apart from TNP, CDs have been applied to detect other nitroaromatic compounds such as TNT. Shi et al. [236] reported a TNT sensor based on FRET-based fluorescence quenching using phosphorus-doped CDs prepared by hydrothermal treatment of a sucrose phosphate solution. The absorption of TNP, centered at 350 nm, overlapped the fluorescence spectrum of the CDs, peaked at 358 nm, giving rise to a FRET process. The as-synthesized CDs exhibited a fluorescence QY of 21.8% and could detect TNT in the linear range of 0.2–17 µM with an LOD of 16.9 nM. On the other hand, Zhang et al. [237] developed multifunctional CDs that exhibited selective detection of TNT with an LOD of 1 nM. TNT could interact with primary amines on the CDs through the formation of TNT-amino complexes and consequently lead to charge transfer from the amino groups to TNT, resulting in PL emission quenching. The degree of PL quenching was proportional to the concentration of TNT. The group also explored electrochemical sensing properties of the as-prepared CDs for enhanced detection of TNT. The reduction current of the first wave at − 0.37 V on a glassy carbon electrode functionalized with CDs was four times higher than that for a bare glassy carbon electrode. This method of detecting TNT provided an improved detection range from 5 nM to 30 µM with a 1 nM LOD.

### Pollutants

The sensing of pollutants related to food products has been of global research interest due to its paramount importance to the health of mankind. For this reason, new sensors able to provide a simpler and affordable alternative for the detection of pollutants in the food and agriculture industries are in high demand. Recently, Zou et al. [238] developed a nitrogen and sulfur-doped CD sensor for fluazinam, a fungicide that was first introduced to avoid crop diseases. However, it has been reported that over-application of fluazinam imposes a serious health effect on humans. The CD synthetic approach in this work employed l-cysteine as the source for C, N, and S. Under an optimal environment at pH 8.8, the sensor demonstrated outstanding selectivity toward fluazinam and negligible interference effects from 17 other potentially interfering substances. Fluazinam tends to bind the N, S-doped CDs via three different interaction paths: (1) hydrogen bonding, (2) *π*–*π* stacking, and (3) electrostatic interactions between the electron-rich N, S-doped CDs and the electron-deficient fluazinam. Successful detection of fluazinam was performed by fluorescence quenching, and a linear detection range from 0.05 to 4 μM was achieved. This study further demonstrated the applicability of the sensor for practical use on fluazinam spiked soil and apple samples, followed by translating the N, S-doped CDs into a physical test strip. While the LOD of aqueous N, S-doped CDs was found to be 10 nM, the test strip version showed LODs of 2.5 mM and 50 μM under sunlight and UV light source excitation, respectively. In another study based on the same analytical method, Xu et al. [239] reported a tartrazine (food colorant) CD sensor fabricated using aloe as the carbon source. The binding of tartrazine to the CDs was monitored through fluorescence quenching of the CDs, and the recorded detection range and LOD for this sensor were 0.25–32.50 μM and 73 nM, respectively. On the other hand, Wang et al. presented nitrogen-doped CDs assembled on an aptamer-modified AuNP sensor for the detection of aflatoxin B_1_ (AFB_1_), a class of carcinogenic metabolites produced during the growth of grains, corn, peanut, etc. Briefly, the electrostatic interaction of nitrogen-doped CDs and the aptamer-modified AuNPs effectively quenched the CD fluorescence, which was restored when AFB_1_ interacted with the aptamer, causing the release of the CDs to the surrounding. The degree of fluorescence recovery was then correlated to the concentration of AFB_1_, ranging from 5.0 pg mL^−1^ to 2.0 ng mL^−1^ with an LOD of 5.0 pg mL^−1^ [240].

A study was conducted by Hou et al. [241] to prepare molecularly imprinted polymers (MIPs) on CDs using microwave irradiation with the aim of detecting tetracycline (Tc) residues in food products. The MIP contributes with a large number of binding sites for Tc absorption. Overlap of the Tc absorption and CD excitation bands generates a FRET system, thus providing eco-friendly and highly efficient quenching performance of the nanoprobe sensor for Tc detection. On top of this, the work demonstrated the feasibility of reusing the sensor for at least six cycles after treatment with methanol/acetic acid (90:10, *v*/*v*) for 10 min to unbind the Tc from the CD surface. The sensor, however, showed affinity toward other Tc analogs, such as oxytetracycline and chlorotetracycline due to the similarity of their chemical structures. Nevertheless, the sensor showed no significant interference from other commonly found substances in food products, such as metal ions, vitamins, and amino acids. This CD-based Tc sensor showed a broad detection range (20 nM to 14 μM) and an LOD of 5.48 nM, which sufficiently meet the standards established by the European Union and US Food and Drug Administration. Similarly, other MIP-CDs involving methacrylic acid and basic 4-vinyl pyridine as the functional monomers were found to enhance the recognition capability for α-amanitin detection [242]. While the study recognized potential selectivity interferences from other substances with similar structural entities to the analogs and MIP template, the broad detection range of 0.05 to 4.0 μg mL^−1^ and LOD of 15 ng mL^−1^ of the CD-based sensor demonstrated a convenient way for the rapid analysis of food complex substances.

Apart from sensing food pollutants, the concept of food safety also includes pesticide detection. In general, organophosphorus compounds (OPs) are the most broadly used pesticides in modern agriculture. However, they pose a serious health hazard and may cause death if consumed above the safety limit. Hou et al. [243] developed l-tyrosine methyl ester-functionalized CDs for the detection of methyl parathion, which is included in the OP family. In the presence of tyrosinase and oxygen, the tyrosine methyl ester was transformed into quinone complexes, thereby quenching the CD fluorescence. However, the presence of methyl parathion reduces the activity rate of tyrosinase and slows down the quenching process. Therefore, the fluorescence intensity can be measured as the concentration of available methyl parathion. Despite its extremely broad detection range, 10 nM to 0.1 mM, and low LOD of 48 pM, the study also confirmed 30-day stability (kept at 4 °C), good reproducibility from different batches of the l-tyrosine methyl ester-functionalized CD-based sensor, and its usability for methyl parathion detection in contaminated food samples such as cabbage, milk, and fruit juice. Another class of OPs, namely dichlorvos, is also of research interest for the same research group [244]. The underlying detection scheme is very similar to that in their previous study, except that the fluorescence is quenched by FRET through the energy acceptor 5-thio-2-nitrobenzoic acid anion (TNB^−^) with quaternized CDs as the energy donor. The presence of dichlorvos inhibits the reaction, further restoring the fluorescence of the CDs, and the concentration can be determined accordingly. Under optimal conditions, a linear detection range of dichlorvos of 50 pM to 0.1 μM with an LOD of 19 pM was achieved. A more recent study was reported by Zheng et al. [245] pertaining to phoxim detection using CD-functionalized silver nanoparticles. Theoretically, the as-prepared CDs become negatively charged in acidic medium (pH 6.0) due to the abundance of amine and carboxyl functional groups on their surface and cross-linked with the positively charged phoxim via electrostatic bonding, resulting in aggregation of the CDs. A visible color change from yellow to red is observed upon addition of phoxim due to the red-shift of the absorption band, from 400 to 525 nm, induced by the aggregation process. Hence, quantitative measurement of the phoxim concentration can be realized through the calculated absorbance ratio, *A*_525nm_/*A*_400nm_, in the detection range of 0.1–100 μM. In addition, the calculated LOD was found to be 0.04 μM, much lower than the maximum permissible phoxim concentration in food samples in China.

Phenol and its analogs have been listed as high-priority pollutants by the US EPA due to the large volume of usage in many industries such as agriculture, paper, plastics, and pharmaceuticals [246]. 4-Nitrophenol, for instance, enters groundwater and surface water as a result of pesticide degradation and it is highly toxic and carcinogenic. For these reasons, a study conducted by Ahmed et al. aimed at delivering a simple, highly sensitive, and reliable CD-based sensor for the determination of 4-nitrophenol in aqueous solution. The fluorescence quenching of the sensor upon introduction of 4-nitrophenol in the system followed a FRET process, which was the strongest in the pH range 8–10 and lasted for 1 h, where the fluorescence intensity remained stable. Although the CD-based 4-nitrophenol sensor showed no potential interferences from other nitro compounds and pesticide degradation, the selectivity of the sensor was rather weak, due to cross-sensitivity toward Fe^3+^ ions. However, the authors claimed that the optimal pH range for 4-nitrophenol would cause Fe^3+^ ions to hydrolyze and precipitate as a hydroxide, thus circumventing their potential to interrupt the sensing of 4-nitrophenol. Overall, the CD-based 4-nitrophenol sensor exhibited a linear detection range from 0.1 to 50 μM with an LOD of 28 nM [247]. The following year, Hao et al. also developed an eco-friendly MIP-CD-based sensor for 4-nitrophenol detection in aqueous samples. The sensor was designed such that the polymer matrix contained abundant recognition sites for 4-nitrophenol to bind the sensor surface through hydrogen bonding, and consequently resulting in fluorescence quenching phenomena due to electron transfer from the CDs to 4-nitrophenol [248]. Although the study did not report on the cross-sensitivity toward Fe^3+^ ions, the group outlined the merits of the developed sensor in terms of reliability, accurate analysis of real samples, linear detection range of 0.2–50 μmol L^−1^, and LOD of 0.06 μmol L^−1^. Meanwhile, Xiao et al. presented boron and nitrogen-doped CDs (B, N-CDs) for *p*-nitrophenol detection, in which the sensing mechanism relied on IFE and the covalent B–O bonds between the B, N-CDs, and *p*-nitrophenol [249]. Interestingly, the B, N-CD sensor was generally stable over a broad pH environment range from 3 to 12 and under continuous UV irradiation for as long as 45 min. Moreover, the study successfully demonstrated improvements as a wider linear detection range (0.5–200 μM) and lower LOD (0.2 μM) compared to those of other similar QD-based *p*-nitrophenol sensors.

Ni et al. proposed a novel, label-free CD-based fluorescent nanoprobe for the straightforward sensing of hydroquinone, a type of phenol contaminant found in industry wastewater and agriculture discharge. The sensing mechanism was simply based on CD fluorescence quenching as a consequence of electron transfer between the probe and target. Under optimal experimental conditions, the amount of fluorescence quenching increased with the concentration of hydroquinone. The work demonstrated that the quenching effect originated from hydroquinone through a validation test using glutathione, a reducing agent for quinone. Significant fluorescence recovery was observed when glutathione was added intentionally to the post-detection mixture of CD-hydroquinone. Furthermore, selectivity testing of the CD sensor using other phenol substances, such as dopamine and catechol, and other metal cations, including Fe^3+^, has proven the high specificity of the sensor toward hydroquinone. A low LOD of 0.1 μM and linear detection range of 0.1–50 μM were achieved [250]. Bisphenol A is known to have deleterious health effects even at sub-ng dosage and may lead to illnesses such as impaired reproductive capacity and diabetes; hence, the need for rapid, accurate, and reliable bisphenol detection is of utmost importance. A new MIP-CD sensor to detect bisphenol A, which experienced a decrease in fluorescence intensity with the increasing bisphenol A concentration, was reported by Liu et al. [251]. The detection was based on interactions between the polymer matrix and bisphenol A through hydrogen bonding and van der Waals forces. While the sensor demonstrated a much lower LOD (30 nmol L^−1^) than those of other published methods, the use of this MIP-CD sensor to measure bisphenol A in real river water samples showed excellent accuracy when compared to conventional HPLC standards.

The green synthesis of CD sensors using lychee seeds was performed by Xue et al. [252] for the highly selective detection of methylene blue, a common color effluent pollutant in wastewater. In the study, the bright blue fluorescence emission (440 nm) from the as-prepared CDs was efficiently quenched by methylene blue within just 5 min of reaction time. The specificity of the CD sensor for methylene blue was investigated against different metal ions and organic dyes, and it was found that methylene blue exhibited significant quenching, indicating negligible effects of the other tested substances on the CD fluorescence. In addition, other forms of methylene blue such as MB^+^, MBH^0^, MBH^2+^, and MB^0^ were also tested, and the outcome of the experiments suggested that methylene blue exists in the form of MB^+^ under the assay conditions used in this work. Apart from a linear methylene blue detection range from 0.2 to 10 μM and LOD of 50 nM, high photostability and low toxicity properties of the CD sensor were demonstrated when evaluated against HepG2 cells, thus unveiling their potential for cellular imaging.

Another pollutant detection study using a CD-based sensor was carried out by Zhao et al. using citrate-stabilized AuNPs with amino-functionalized CDs on their surface for the detection of thiocyanate anions, a derivative from various industrial sources, such as electroplating, fabric dyes, and hydrometallurgy, that could possibly transform into highly toxic cyanide. The as-prepared sensor worked in dual-mode, such that the AuNPs serve as a colorimetric indicator for thiocyanate sensing and also a CD fluorescence quencher via FRET. In short, the assembly of amino-functionalized CDs on citrate-stabilized AuNPs causes aggregation of the AuNPs (visible blue solution) and fluorescence quenching of the CDs. However, when thiocyanate is introduced in the system, it competes with the CDs for the binding sites on the AuNPs, inhibiting the aggregation process and resulting in a visible red solution and unquenched CD fluorescence. A rather small detection range (LOD of 0.2–2 μM and 0.14 μM, and of 0.1–1.6 μM and 0.036 μM) was recorded by both colorimetric and fluorometric analyses for thiocyanate detection, respectively [253].

### Vitamins and Drugs

**Fig. 20:**
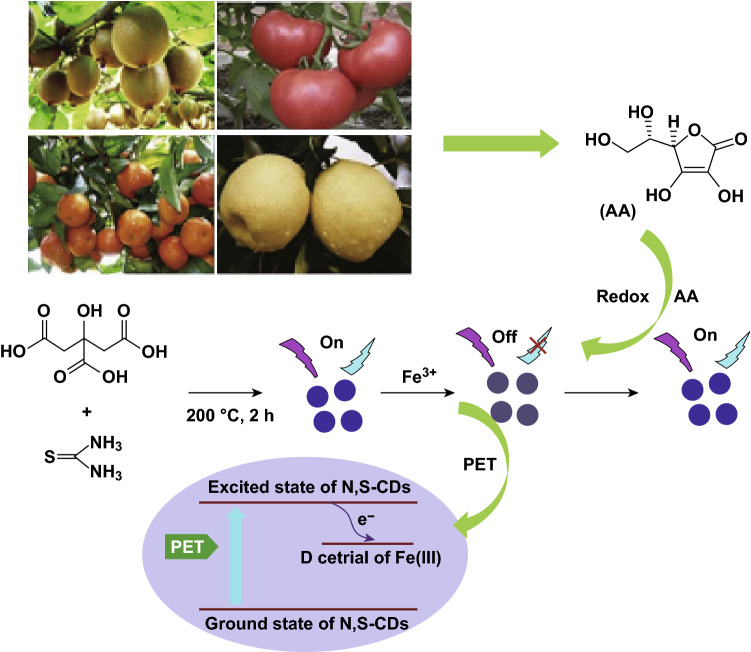
Schematic illustration of N, S-co-doped CDs for the detection of ascorbic acid in fruit [254]. Copyright © 2018 Elsevier Ltd.

By exploiting on–off–on processes, CDs can serve as a multifunctional fluorescent probe capable of detecting vitamins and drugs. For instance, Luo et al. [254] developed on–off–on nitrogen and sulfur co-doped CDs for the detection of ascorbic acid in fruit (Fig. 20). The fluorescence of CDs was quenched by Fe^3+^ ions due to an electron transfer process, but the fluorescence was recovered gradually with the increasing concentration of ascorbic acid in the sensing system, which can be explained by the redox reaction between Fe^3+^ and ascorbic acid. The enediol group of ascorbic acid is oxidized to the ortho-dione group of dehydroascorbic acid, accompanied by the reduction of Fe^3+^ to Fe^2+^, which has weaker capability to interact with CDs thus restoring their fluorescence. This sensory system has a reported LOD of 4.69 µmol L^−1^ with a linear range of 10–200 µmol L^−1^. Similarly, Liu et al. [255] reported a CD-MnO_2_ sensing system capable of detecting ascorbic acid in fresh fruits, vegetables, and commercial fruit juice samples. The fluorescence of CDs was first quenched upon optimal addition of MnO_2_ by IFE to establish a CD-MnO_2_ fluorescence probe. The fluorescence of CDs was recovered in the presence of ascorbic acid by redox reaction with MnO_2._ The LOD was found to be 42 nM with a wide linear range of 0.18–90 µM. Vitamin B12 has also been detected using fluorescent CDs. The excitation light of CDs can be absorbed by vitamin B12, resulting in a decrease in fluorescence intensity, which was attributed to IFE [256]. The proposed sensor provided a linear detection range from 0 to 60 µM with an LOD of 0.1 µM.

**Fig. 21:**
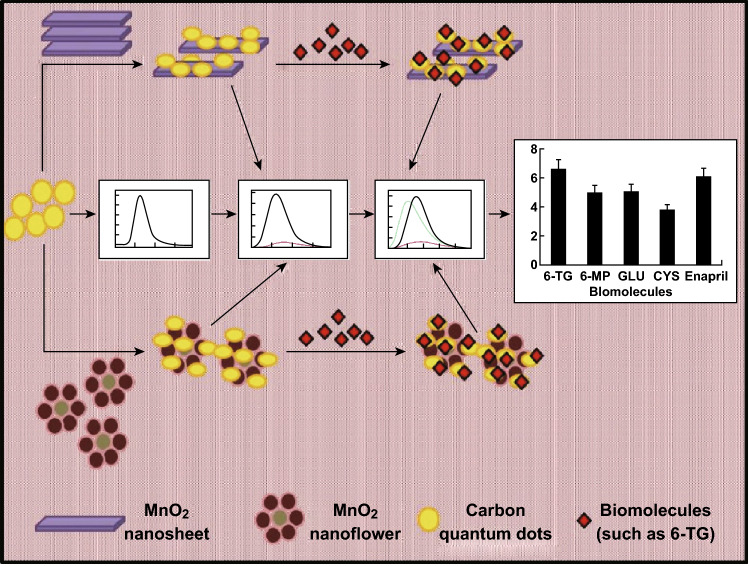
Schematic illustration of the proposed CQD-based “off–on” sensor for 6-TG [258]. Copyright © 2017 Elsevier B.V.

Many different anticancer drugs and antibiotics can be detected using fluorescent CD assays. Zeng et al. [257] introduced polyethyleneimine-modified CDs for the fast determination of 6-thioguanine (6-TG). The introduction of polyethyleneimine contributes to the introduction of –NH groups on the surface of CDs, which can strongly bind 6-TG. A PET process takes place, where photoexcited electrons transfer from CDs to 6-TG, resulting in a non-radiative pathway. The nanoprobe could operate from 4 to 800 µM with an LOD of 1.33 µM, and could be further applied for 6-TG detection in human serum and urine samples. To further improve the sensitivity of the nanoprobe, Garg et al. [258] developed a FRET-based system using CQDs as the electron donor and MnO_2_ nanosheets or nanoflowers as the electron acceptor (Fig. 21). The fluorescence of the CDs was quenched upon introduction of MnO_2_ particles. However, the fluorescence was restored upon addition of anticancer drug 6-TG and 6-mercaptopurine (6-MP) due to the formation of Mn–S bonds. This system exhibited LODs of 0.015 and 0.014 µM for 6-TG and 6-MP, respectively. The detection of other types of drugs, such as oxytetracycline [259], chlortetracycline and doxycycline [260], glucosamine [71], kanamycin [261], quercetin [262], lidocaine hydrochloride [263], has been reported using CD-based fluorescence nanoprobes using various quenching mechanism including PET, IFE, and FRET.

## Conclusion

As the youngest member in the carbon family, CDs have evolved into one of the most versatile nanomaterials, finding applications ranging from biological imaging, cancer therapy, display technology, energy storage, photocatalysis, and sensing technology. Several reasons make them an outstanding alternative to other QDs. First, the synthesis routes are generally simpler and can be prepared from a variety of carbon sources such as chemicals, green materials, and even waste. Second, the surface functionalization is simple and can render CDs with specific functionalities. Third, CDs have excellent physical properties, unique optical properties, and outstanding biocompatibility. Fourth, their performance in sensing applications can be enhanced by complexation with other fluorophores. Comparatively, other metal-free QDs such as black phosphorus QDs need to be prepared from a specific precursor, limiting the choice of suitable precursors. On the other hand, other members of the carbon family such as carbon nanotubes are commonly prepared using sophisticated methods such as chemical vapor deposition (CVD), laser ablation, and carbon arc-discharge techniques [264]. Graphene and graphene oxide are primarily prepared using CVD growth or exfoliation techniques such as the cases of Hummer’s, Brodie’s, and Staudenmaier’s methods [265]. Some of these methods have a high yield of over 75% but are often limited by the need for high experimental temperatures, specific equipment, toxic chemicals, and specific precursors.

Although many methods have been developed to prepare fluorescent CDs, the synthesis mechanism is still not fully understood for the precise control of the size, surface functionalities, and optical properties. For instance, microwave-assisted heating of citric acid in formamide solution has been reported by Sun et al. [266, 267] to afford red-emission CDs. Red emission was not observed when citric acid was heated up in other solvents such as water, ethanol, and dimethylformamide. Therefore, the contributing factors of the solvent to the formation mechanism of red-emission CDs need to be further investigated. Moreover, for CD-based sensors to be translated for commercialization purposes, the large-scale production of CDs with uniform properties is required, preferably from economic and environmentally friendly carbon sources. To date, most large-scale synthesis reports involve the preparation of CDs at gram scale, which needs to be further increased. Also, the preparation of CDs, especially from green precursors, can be a bottle-neck for reproducibility, since there are many uncontrollable factors affecting the carbon precursors. CDs prepared from different green precursors such as hair and urine can have widely different properties due to different genetics, diets, and other factors that may influence the composition of the precursors.

The PL mechanisms of CDs, both down-conversion and UCPL, are still under debate, suggesting that more theoretical and experimental work is needed to elucidate the CD PL origin. Advanced spectroscopy techniques, such as single molecule fluorescence microscopy, lifetime microscopy, and super resolution techniques, could provide explanations for the PL properties at the single particle level and identify the exact components of CDs that are responsible for a specific emission. Theoretical modeling such as materials simulation is a useful approach to understand the relationship between the precursor structure and the characteristics of CDs. This will allow the fast and accurate selection of precursors to modulate the surface functional groups and subsequently control the emission properties of CDs. In addition, CDs with broad absorption spectra, narrow emission spectra, large Stokes shifts, and emission in the long-wavelength region are highly desirable. These would allow multiplex fluorescence studies where narrow and non-overlapping emission spectra of different CDs can be detected simultaneously with a single excitation wavelength. CDs have comparable PL QYs in the blue and green emission range with semiconductor QDs; however, most reported CDs still have less-desirable red and near-infrared emission with low PL QYs. New strategies must be developed to increase the PL QY in the long-wavelength region, as this will open up a new avenue for biological applications.

Many groups have developed label-free CDs for various sensing applications. However, the selectivity and sensitivity of the nanoprobes toward the analytes are solely determined by the type of precursors. As shown in Table 2, the precursors to prepare CDs capable of detecting Fe^3+^ ions can originate from different chemical and green sources. They have similar selectivity toward Fe^3+^ ions but with vastly different sensitivity and linear operational ranges. Hence, careful selection of precursors may allow control over the selectivity and linear operating range toward a specific target analyte. In addition, complexation with other materials through covalent binding could greatly enhance the sensitivity and selectivity of nanoprobe systems. The complexation allows the nanoprobe system to work as a ratiometric or colorimetric sensor, which could be used for on-site detection without the need for expensive and bulky laboratory equipment. Functionalization of CDs with specific ligands is another approach to detect the corresponding targets. For instance, boronic acid groups can serve as binding sites for sensitive and selective glucose detection. Likewise, through careful control of the surface functional groups, CDs with multiple emissions will provide the capability to detect multiple types of analytes.

While most on-site detection only offers qualitative results (i.e., colorimetric changes in the presence of analytes), more efforts can be channeled into microfluidic and lab-on-chip technologies, where a single chip may display qualitative and quantitative results within a short period of time. Lastly, it can be foreseen that the widespread use of CDs may pose a danger to the environment and human beings. Even though CDs have been widely reported to be biocompatible and of low toxicity, their potential environmental release cannot be avoided and said CDs may find their way into the food chain and human body. Thus, investigations on the nanotoxicity of CDs, as well as green and environmentally friendly preparations of CDs with excellent properties are highly desirable.

## Abbreviations

CDs: Carbon dots
CNDs: Carbon nanodots
CQDs: Carbon quantum dots
NMR: Nuclear magnetic resonance
BPEI: Branched polyethylenimine
PEG: Polyethylene glycol
PL: Photoluminescence
UCPL: Up-conversion photoluminescence
NIR: Near infrared
LOD: Limit of detection
UV: Ultraviolet
HOMO: Highest occupied molecular orbital
LUMO: Lowest unoccupied molecular orbitals
QY: Quantum yield
DC: Direct current
PAA: Poly(acrylic acid)
P(AGA)(AA): Poly(acryloylglucosamine)(acrylic acid)
RAFT: Reversible addition–fragmentation chain transfer
AGA: *N*-acryloyl-d-glucosamine
TOAB: Tetraoctyl ammonium bromide
APBA: 3-Aminophenylboronic acid
EDC: 1-Ethyl-3-(3-dimethylaminopropyl) carbodiimide
AuNP: Gold nanoparticles
PET: Photo-induced electron transfer
FRET: Forster resonance energy transfer
IFE: Inner filter effect
ppb: Parts per billion
GSH: Glutathione
US EPA: United States Environmental Protection Agency
MB: Methylene blue
MnO_2_: Manganese dioxide
6-TG: 6-Thioguanine
CdTe: Cadmium telluride
BONs: Breakable organosilica nanocapsules
CNT: Carbon nanotubes

## References

[1] Ward RJ, Zucca FA, Duyn JH, Crichton RR, Zecca L (2014). The role of iron in brain ageing and neurodegenerative disorders. Lancet Neurol..

[2] Huang X (2003). Iron overload and its association with cancer risk in humans: evidence for iron as a carcinogenic metal. Mutat. Res.-Fund. Mol. M..

[3] Deugnier Y (2003). Iron and liver cancer. Alcohol.

[4] Toyokuni S (2009). Role of iron in carcinogenesis: cancer as a ferrotoxic disease. Cancer Sci..

[5] Bouchard MF, Sauvé S, Barbeau B, Legrand M, Brodeur M-È, Bouffard T, Limoges E, Bellinger DC, Mergler D (2011). Intellectual impairment in school-age children exposed to manganese from drinking water. Environ. Health Persp..

[6] Zhao J, Yan X, Zhou T, Wang J, Li H, Zhang P, Ding H, Ding L (2015). Multi-throughput dynamic microwave-assisted leaching coupled with inductively coupled plasma atomic emission spectrometry for heavy metal analysis in soil. J. Anal. Atom. Spectrom..

[7] Feist B, Mikula B (2014). Preconcentration of heavy metals on activated carbon and their determination in fruits by inductively coupled plasma optical emission spectrometry. Food Chem..

[8] Benipal G, Harris A, Srirajayatsayai C, Tate A, Topalidis V (2017). Examination of Al, As, Cd, Cr, Cu, Fe, Mg, Mn, Ni, Pb, Sb, Se, V, and Zn in sediments collected around the downtown Houston, Texas area, using inductively coupled plasma-optical emission spectroscopy. Microchem. J..

[9] Behbahani M, Tapeh NAG, Mahyari M, Pourali AR, Amin BG, Shaabani A (2014). Monitoring of trace amounts of heavy metals in different food and water samples by flame atomic absorption spectrophotometer after preconcentration by amine-functionalized graphene nanosheet. Environ. Monit. Assess..

[10] Daşbaşı T, Saçmacı Ş, Ülgen A, Kartal Ş (2015). A solid phase extraction procedure for the determination of Cd(II) and Pb(II) ions in food and water samples by flame atomic absorption spectrometry. Food Chem..

[11] Cui L, Wu J, Ju H (2015). Electrochemical sensing of heavy metal ions with inorganic, organic and bio-materials. Biosens. Bioelectron..

[12] Xu G, Zeng S, Zhang B, Swihart MT, Yong K-T, Prasad PN (2016). New generation cadmium-free quantum dots for biophotonics and nanomedicine. Chem. Rev..

[13] Georgakilas V, Perman JA, Tucek J, Zboril R (2015). Broad family of carbon nanoallotropes: classification, chemistry, and applications of fullerenes, carbon dots, nanotubes, graphene, nanodiamonds, and combined superstructures. Chem. Rev..

[14] Xu X, Ray R, Gu Y, Ploehn HJ, Gearheart L, Raker K, Scrivens WA (2004). Electrophoretic analysis and purification of fluorescent single-walled carbon nanotube fragments. J. Am. Chem. Soc..

[15] Sun Y-P, Zhou B, Lin Y, Wang W, Fernando KS (2006). Quantum-sized carbon dots for bright and colorful photoluminescence. J. Am. Chem. Soc..

[16] Bottini M, Balasubramanian C, Dawson MI, Bergamaschi A, Bellucci S, Mustelin T (2006). Isolation and characterization of fluorescent nanoparticles from pristine and oxidized electric arc-produced single-walled carbon nanotubes. J. Phys. Chem. B.

[17] Gonçalves H, Jorge PA, Fernandes J, da Silva JCE (2010). Hg(II) sensing based on functionalized carbon dots obtained by direct laser ablation. Sensor. Actuat. B-Chem..

[18] Li X, Wang H, Shimizu Y, Pyatenko A, Kawaguchi K, Koshizaki N (2011). Preparation of carbon quantum dots with tunable photoluminescence by rapid laser passivation in ordinary organic solvents. Chem. Commun..

[19] Hu S, Liu J, Yang J, Wang Y, Cao S (2011). Laser synthesis and size tailor of carbon quantum dots. J. Nanopart. Res..

[20] Bao L, Zhang ZL, Tian ZQ, Zhang L, Liu C, Lin Y, Qi B, Pang DW (2011). Electrochemical tuning of luminescent carbon nanodots: from preparation to luminescence mechanism. Adv. Mater..

[21] Liu M, Xu Y, Niu F, Gooding JJ, Liu J (2016). Carbon quantum dots directly generated from electrochemical oxidation of graphite electrodes in alkaline alcohols and the applications for specific ferric ion detection and cell imaging. Analyst.

[22] Hou Y, Lu Q, Deng J, Li H, Zhang Y (2015). One-pot electrochemical synthesis of functionalized fluorescent carbon dots and their selective sensing for mercury ion. Anal. Chim. Acta.

[23] Deng J, Lu Q, Mi N, Li H, Liu M (2014). Electrochemical synthesis of carbon nanodots directly from alcohols. Chem.-Eur. J..

[24] Tao H, Yang K, Ma Z, Wan J, Zhang Y, Kang Z, Liu Z (2012). In vivo NIR fluorescence imaging, biodistribution, and toxicology of photoluminescent carbon dots produced from carbon nanotubes and graphite. Small.

[25] Sun D, Ban R, Zhang P-H, Wu G-H, Zhang J-R, Zhu J-J (2013). Hair fiber as a precursor for synthesizing of sulfur-and nitrogen-co-doped carbon dots with tunable luminescence properties. Carbon.

[26] Chang MMF, Ginjom IR, Ngu-Schwemlein M, Ng SM (2016). Synthesis of yellow fluorescent carbon dots and their application to the determination of chromium(III) with selectivity improved by pH tuning. Microchim. Acta.

[27] Hu Y, Yang J, Tian J, Jia L, Yu J-S (2014). Waste frying oil as a precursor for one-step synthesis of sulfur-doped carbon dots with pH-sensitive photoluminescence. Carbon.

[28] Subramanian V, Zhu H, Vajtai R, Ajayan P, Wei B (2005). Hydrothermal synthesis and pseudocapacitance properties of MnO_2_ nanostructures. J. Phys. Chem. B.

[29] Meligrana G, Gerbaldi C, Tuel A, Bodoardo S, Penazzi N (2006). Hydrothermal synthesis of high surface LiFePO4 powders as cathode for Li-ion cells. J. Power Sources.

[30] Liang Q, Ma W, Shi Y, Li Z, Yang X (2013). Easy synthesis of highly fluorescent carbon quantum dots from gelatin and their luminescent properties and applications. Carbon.

[31] Xu Q, Pu P, Zhao J, Dong C, Gao C, Chen Y, Chen J, Liu Y, Zhou H (2015). Preparation of highly photoluminescent sulfur-doped carbon dots for Fe(III) detection. J. Mater. Chem. A.

[32] Li J-Y, Liu Y, Shu Q-W, Liang J-M, Zhang F, Chen X-P, Deng X-Y, Swihart MT, Tan K-J (2017). One-pot hydrothermal synthesis of carbon dots with efficient up-and down-converted photoluminescence for the sensitive detection of morin in a dual-readout assay. Langmuir.

[33] Mehta VN, Jha S, Basu H, Singhal RK, Kailasa SK (2015). One-step hydrothermal approach to fabricate carbon dots from apple juice for imaging of mycobacterium and fungal cells. Sensor. Actuat. B-Chem..

[34] Wang L, Zhou HS (2014). Green synthesis of luminescent nitrogen-doped carbon dots from milk and its imaging application. Anal. Chem..

[35] Edison TNJI, Atchudan R, Shim J-J, Kalimuthu S, Ahn B-C, Lee YR (2016). Turn-off fluorescence sensor for the detection of ferric ion in water using green synthesized N-doped carbon dots and its bio-imaging. J. Photoch. Photobio. B.

[36] Atchudan R, Edison TNJI, Sethuraman MG, Lee YR (2016). Efficient synthesis of highly fluorescent nitrogen-doped carbon dots for cell imaging using unripe fruit extract of Prunus mume. Appl. Surf. Sci..

[37] Liu Y, Zhou Q, Li J, Lei M, Yan X (2016). Selective and sensitive chemosensor for lead ions using fluorescent carbon dots prepared from chocolate by one-step hydrothermal method. Sensor. Actuat. B-Chem..

[38] Yu J, Song N, Zhang Y-K, Zhong S-X, Wang A-J, Chen J (2015). Green preparation of carbon dots by Jinhua bergamot for sensitive and selective fluorescent detection of Hg^2+^ and Fe^3+^. Sensor. Actuat. B-Chem..

[39] Wei J, Zhang X, Sheng Y, Shen J, Huang P, Guo S, Pan J, Feng B (2014). Dual functional carbon dots derived from cornflour via a simple one-pot hydrothermal route. Mater. Lett..

[40] Li L, Wang X, Fu Z, Cui F (2017). One-step hydrothermal synthesis of nitrogen-and sulfur-co-doped carbon dots from ginkgo leaves and application in biology. Mater. Lett..

[41] Liu R, Zhang J, Gao M, Li Z, Chen J, Wu D, Liu P (2015). A facile microwave-hydrothermal approach towards highly photoluminescent carbon dots from goose feathers. RSC Adv..

[42] Sahu S, Behera B, Maiti TK, Mohapatra S (2012). Simple one-step synthesis of highly luminescent carbon dots from orange juice: application as excellent bio-imaging agents. Chem. Commun..

[43] Purbia R, Paria S (2016). A simple turn on fluorescent sensor for the selective detection of thiamine using coconut water derived luminescent carbon dots. Biosens. Bioelectron..

[44] Niu J, Gao H (2014). Synthesis and drug detection performance of nitrogen-doped carbon dots. J. Lumin..

[45] Chandra S, Chowdhuri AR, Laha D, Sahu SK (2017). Fabrication of nitrogen-and phosphorous-doped carbon dots by the pyrolysis method for iodide and iron(III) sensing. Luminescence.

[46] Mao L-H, Tang W-Q, Deng Z-Y, Liu S-S, Wang C-F, Chen S (2014). Facile access to white fluorescent carbon dots toward light-emitting devices. Ind. Eng. Chem. Res..

[47] Wang F, Pang S, Wang L, Li Q, Kreiter M, Liu C-Y (2010). One-step synthesis of highly luminescent carbon dots in noncoordinating solvents. Chem. Mater..

[48] He H, Wang X, Feng Z, Cheng T, Sun X, Sun Y, Xia Y, Wang S, Wang J, Zhang X (2015). Rapid microwave-assisted synthesis of ultra-bright fluorescent carbon dots for live cell staining, cell-specific targeting and in vivo imaging. J. Mater. Chem. B.

[49] Li H, Xu Y, Ding J, Zhao L, Zhou T, Ding H, Chen Y, Ding L (2018). Microwave-assisted synthesis of highly luminescent N-and S-co-doped carbon dots as a ratiometric fluorescent probe for levofloxacin. Microchim. Acta.

[50] Xiao Q, Liang Y, Zhu F, Lu S, Huang S (2017). Microwave-assisted one-pot synthesis of highly luminescent N-doped carbon dots for cellular imaging and multi-ion probing. Microchim. Acta.

[51] Kwon W, Rhee S-W (2012). Facile synthesis of graphitic carbon quantum dots with size tunability and uniformity using reverse micelles. Chem. Commun..

[52] Zhang J, Abbasi F, Claverie J (2015). An efficient templating approach for the synthesis of redispersible size-controllable carbon quantum dots from graphitic polymeric micelles. Chem.-Eur. J..

[53] Linehan K, Doyle H (2014). Efficient one-pot synthesis of highly monodisperse carbon quantum dots. RSC Adv..

[54] Liu R, Wu D, Liu S, Koynov K, Knoll W, Li Q (2009). An aqueous route to multicolor photoluminescent carbon dots using silica spheres as carriers. Angew. Chem..

[55] Yang Y, Wu D, Han S, Hu P, Liu R (2013). Bottom-up fabrication of photoluminescent carbon dots with uniform morphology via a soft–hard template approach. Chem. Commun..

[56] Wu ZL, Liu ZX, Yuan YH (2017). Carbon dots: materials, synthesis, properties and approaches to long-wavelength and multicolor emission. J. Mater. Chem. B.

[57] Sun X, Lei Y (2017). Fluorescent carbon dots and their sensing applications. TrAC-Trend. Anal. Chem..

[58] Hu S-L, Niu K-Y, Sun J, Yang J, Zhao N-Q, Du X-W (2009). One-step synthesis of fluorescent carbon nanoparticles by laser irradiation. J. Mater. Chem..

[59] Qu S, Wang X, Lu Q, Liu X, Wang L (2012). A biocompatible fluorescent ink based on water-soluble luminescent carbon nanodots. Angew. Chem. Int.-Ed..

[60] Qu D, Zheng M, Du P, Zhou Y, Zhang L (2013). Highly luminescent S, N co-doped graphene quantum dots with broad visible absorption bands for visible light photocatalysts. Nanoscale.

[61] Dong Y, Wang R, Li H, Shao J, Chi Y, Lin X, Chen G (2012). Polyamine-functionalized carbon quantum dots for chemical sensing. Carbon.

[62] Liu C, Zhang P, Zhai X, Tian F, Li W, Yang J, Liu Y, Wang H, Wang W, Liu W (2012). Nano-carrier for gene delivery and bioimaging based on carbon dots with PEI-passivation enhanced fluorescence. Biomaterials.

[63] Yang Z, Xu M, Liu Y, He F, Gao F, Su Y, Wei H, Zhang Y (2014). Nitrogen-doped, carbon-rich, highly photoluminescent carbon dots from ammonium citrate. Nanoscale.

[64] Bourlinos AB, Trivizas G, Karakassides MA, Baikousi M, Kouloumpis A, Gournis D, Bakandritsos A, Hola K, Kozak O, Zboril R (2015). Green and simple route toward boron doped carbon dots with significantly enhanced non-linear optical properties. Carbon.

[65] Qian Z, Shan X, Chai L, Ma J, Chen J, Feng H (2014). Si-doped carbon quantum dots: a facile and general preparation strategy, bioimaging application, and multifunctional sensor. ACS Appl. Mater. Interfaces..

[66] Qiao F, Wang J, Ai S, Li L (2015). As a new peroxidase mimetics: The synthesis of selenium doped graphitic carbon nitride nanosheets and applications on colorimetric detection of H_2_O_2_ and xanthine. Sensor. Actuat. B-Chem..

[67] Gong N, Wang H, Li S, Deng Y, Chen XA, Ye L, Gu W (2014). Microwave-assisted polyol synthesis of gadolinium-doped green luminescent carbon dots as a bimodal nanoprobe. Langmuir.

[68] Li H, Shao F-Q, Huang H, Feng J-J, Wang A-J (2016). Eco-friendly and rapid microwave synthesis of green fluorescent graphitic carbon nitride quantum dots for vitro bioimaging. Sensor. Actuat. B-Chem..

[69] Ganiga M, Cyriac J (2016). FRET based ammonia sensor using carbon dots. Sensor. Actuat. B-Chem..

[70] Han C, Wang R, Wang K, Xu H, Sui M, Li J, Xu K (2016). Highly fluorescent carbon dots as selective and sensitive on-off-on probes for iron(III) ion and apoferritin detection and imaging in living cells. Biosens. Bioelectron..

[71] Das RK, Mohapatra S (2017). Highly luminescent, heteroatom-doped carbon quantum dots for ultrasensitive sensing of glucosamine and targeted imaging of liver cancer cells. J. Mater. Chem. B.

[72] Jiang K, Sun S, Zhang L, Lu Y, Wu A, Cai C, Lin H (2015). Red, green, and blue luminescence by carbon dots: full-color emission tuning and multicolor cellular imaging. Angew. Chem. Int.-Ed..

[73] Pan L, Sun S, Zhang A, Jiang K, Zhang L, Dong C, Huang Q, Wu A, Lin H (2015). Truly fluorescent excitation-dependent carbon dots and their applications in multicolor cellular imaging and multidimensional sensing. Adv. Mater..

[74] Zhang Y, He J (2015). Facile synthesis of S, N co-doped carbon dots and investigation of their photoluminescence properties. Phys. Chem. Chem. Phys..

[75] Li X, Zhang S, Kulinich SA, Liu Y, Zeng H (2014). Engineering surface states of carbon dots to achieve controllable luminescence for solid-luminescent composites and sensitive Be^2+^ detection. Sci. Rep..

[76] Wang H, Sun C, Chen X, Zhang Y, Colvin VL (2017). Excitation wavelength independent visible color emission of carbon dots. Nanoscale.

[77] Meng X, Chang Q, Xue C, Yang J, Hu S (2017). Full-colour carbon dots: from energy-efficient synthesis to concentration-dependent photoluminescence properties. Chem. Commun..

[78] Wang C, Xu Z, Cheng H, Lin H, Humphrey MG, Zhang C (2015). A hydrothermal route to water-stable luminescent carbon dots as nanosensors for pH and temperature. Carbon.

[79] Jia X, Li J, Wang E (2012). One-pot green synthesis of optically pH-sensitive carbon dots with upconversion luminescence. Nanoscale.

[80] Jin SH, Kim DH, Jun GH, Hong SH, Jeon S (2013). Tuning the photoluminescence of graphene quantum dots through the charge transfer effect of functional groups. ACS Nano.

[81] Dong Y, Pang H, Yang HB, Guo C, Shao J, Chi Y, Li CM, Yu T (2013). Carbon-based dots Co-doped with nitrogen and sulfur for high quantum yield and excitation-independent emission. Angew. Chem. Int.-Ed..

[82] Li H, Ming H, Liu Y, Yu H, He X, Huang H, Pan K, Kang Z, Lee S-T (2011). Fluorescent carbon nanoparticles: electrochemical synthesis and their pH sensitive photoluminescence properties. New J. Chem..

[83] Yu P, Wen X, Toh Y-R, Tang J (2012). Temperature-dependent fluorescence in carbon dots. J. Phys. Chem. C.

[84] Chen P-C, Chen Y-N, Hsu P-C, Shih C-C, Chang H-T (2013). Photoluminescent organosilane-functionalized carbon dots as temperature probes. Chem. Commun..

[85] Cao L, Wang X, Meziani MJ, Lu F, Wang H (2007). Carbon dots for multiphoton bioimaging. J. Am. Chem. Soc..

[86] Salinas-Castillo A, Ariza-Avidad M, Pritz C, Camprubí-Robles M, Fernández B (2013). Carbon dots for copper detection with down and upconversion fluorescent properties as excitation sources. Chem. Commun..

[87] Yin B, Deng J, Peng X, Long Q, Zhao J, Lu Q, Chen Q, Li H, Tang H, Zhang Y (2013). Green synthesis of carbon dots with down-and up-conversion fluorescent properties for sensitive detection of hypochlorite with a dual-readout assay. Analyst.

[88] Zhang C, Du L, Liu C, Li Y, Yang Z, Cao Y-C (2016). Photostable epoxy polymerized carbon quantum dots luminescent thin films and the performance study. Results Phys..

[89] da Silva JCE, Gonçalves HM (2011). Analytical and bioanalytical applications of carbon dots. TrAC-Trend. Anal. Chem..

[90] Li H, He X, Kang Z, Huang H, Liu Y, Liu J, Lian S, Tsang CHA, Yang X, Lee ST (2010). Water-soluble fluorescent carbon quantum dots and photocatalyst design. Angew. Chem. Int.-Ed..

[91] Kwon W, Lee G, Do S, Joo T, Rhee SW (2014). Size-controlled soft-template synthesis of carbon nanodots toward versatile photoactive materials. Small.

[92] Zhu S, Song Y, Zhao X, Shao J, Zhang J, Yang B (2015). The photoluminescence mechanism in carbon dots (graphene quantum dots, carbon nanodots, and polymer dots): current state and future perspective. Nano Res..

[93] Ding H, Yu S-B, Wei J-S, Xiong H-M (2015). Full-color light-emitting carbon dots with a surface-state-controlled luminescence mechanism. ACS Nano.

[94] Li Q, Ohulchanskyy TY, Liu R, Koynov K, Wu D, Best A, Kumar R, Bonoiu A, Prasad PN (2010). Photoluminescent carbon dots as biocompatible nanoprobes for targeting cancer cells in vitro. J. Phys. Chem. C.

[95] Krysmann MJ, Kelarakis A, Dallas P, Giannelis EP (2011). Formation mechanism of carbogenic nanoparticles with dual photoluminescence emission. J. Am. Chem. Soc..

[96] Ma Z, Ming H, Huang H, Liu Y, Kang Z (2012). One-step ultrasonic synthesis of fluorescent N-doped carbon dots from glucose and their visible-light sensitive photocatalytic ability. New J. Chem..

[97] Wen X, Yu P, Toh Y-R, Ma X, Tang J (2014). On the upconversion fluorescence in carbon nanodots and graphene quantum dots. Chem. Commun..

[98] Jiang G, Jiang T, Li X, Wei Z, Du X, Wang X (2014). Boronic acid functionalized N-doped carbon quantum dots as fluorescent probe for selective and sensitive glucose determination. Mater. Res. Express.

[99] Zhang Z, Shi Y, Pan Y, Cheng X, Zhang L, Chen J, Li M-J, Yi C (2014). Quinoline derivative-functionalized carbon dots as a fluorescent nanosensor for sensing and intracellular imaging of Zn^2+^. J. Mater. Chem. B.

[100] Amjadi M, Abolghasemi-Fakhri Z, Hallaj T (2015). Carbon dots-silver nanoparticles fluorescence resonance energy transfer system as a novel turn-on fluorescent probe for selective determination of cysteine. J. Photoch. Photobio. A.

[101] Qu F, Pei H, Kong R, Zhu S, Xia L (2017). Novel turn-on fluorescent detection of alkaline phosphatase based on green synthesized carbon dots and MnO_2_ nanosheets. Talanta.

[102] Guo Y, Yang L, Li W, Wang X, Shang Y, Li B (2016). Carbon dots doped with nitrogen and sulfur and loaded with copper(II) as a turn-on fluorescent probe for cystein, glutathione and homocysteine. Microchim. Acta.

[103] Li L, Wang C, Liu K, Wang Y, Liu K, Lin Y (2015). Hexagonal cobalt oxyhydroxide–carbon dots hybridized surface: high sensitive fluorescence turn-on probe for monitoring of ascorbic acid in rat brain following brain ischemia. Anal. Chem..

[104] Yuan C, Liu B, Liu F, Han M-Y, Zhang Z (2014). Fluorescence turn on detection of mercuric ion based on bis (dithiocarbamato) copper(II) complex functionalized carbon nanodots. Anal. Chem..

[105] Cayuela A, Soriano ML, Carrión MC, Valcárcel M (2014). Functionalized carbon dots as sensors for gold nanoparticles in spiked samples: formation of nanohybrids. Anal. Chim. Acta.

[106] Zheng P, Wu N (2017). Fluorescence and sensing applications of graphene oxide and graphene quantum dots: a review. Chem.-Asian J..

[107] Medintz IL, Clapp AR, Mattoussi H, Goldman ER, Fisher B, Mauro JM (2003). Self-assembled nanoscale biosensors based on quantum dot FRET donors. Nat. Mater..

[108] Zhang Y, Liu X, Fan Y, Guo X, Zhou L, Lv Y, Lin J (2016). One-step microwave synthesis of N-doped hydroxyl-functionalized carbon dots with ultra-high fluorescence quantum yields. Nanoscale.

[109] Albrecht C, Lakowicz JR (2008). Principles of fluorescence spectroscopy. Anal. Bioanal. Chem..

[110] Chen S, Yu Y-L, Wang J-H (2017). Inner filter effect-based fluorescent sensing systems: a review. Anal. Chim. Acta.

[111] Gong X, Lu W, Paau MC, Hu Q, Wu X, Shuang S, Dong C, Choi MM (2015). Facile synthesis of nitrogen-doped carbon dots for Fe^3+^ sensing and cellular imaging. Anal. Chim. Acta.

[112] Shangguan J, Huang J, He D, He X, Wang K, Ye R, Yang X, Qing T, Tang J (2017). Highly Fe^3+^-selective fluorescent nanoprobe based on ultrabright N/P codoped carbon dots and its application in biological samples. Anal. Chem..

[113] Liu W, Diao H, Chang H, Wang H, Li T, Wei W (2017). Green synthesis of carbon dots from rose-heart radish and application for Fe^3+^ detection and cell imaging. Sensor. Actuat. B-Chem..

[114] Rong M, Feng Y, Wang Y, Chen X (2017). One-pot solid phase pyrolysis synthesis of nitrogen-doped carbon dots for Fe^3+^ sensing and bioimaging. Sensor. Actuat. B-Chem..

[115] Liu Y, Duan W, Song W, Liu J, Ren C, Wu J, Liu D, Chen H (2017). Red emission B, N, S-co-doped carbon dots for colorimetric and fluorescent dual mode detection of Fe^3+^ ions in complex biological fluids and living cells. ACS Appl. Mater. Interfaces..

[116] Wu J, Feng Y, Shao Y, Sun Y (2018). High quality nitrogen and silicon Co-doped carbon dots (N/Si-CDs) for Fe^3+^ sensing. J. Nanosci. Nanotechnol..

[117] Chandra S, Laha D, Pramanik A, Ray Chowdhuri A, Karmakar P, Sahu SK (2016). Synthesis of highly fluorescent nitrogen and phosphorus doped carbon dots for the detection of Fe^3+^ ions in cancer cells. Luminescence.

[118] He G, Xu M, Shu M, Li X, Yang Z, Zhang L, Su Y, Hu N, Zhang Y (2016). Rapid solid-phase microwave synthesis of highly photoluminescent nitrogen-doped carbon dots for Fe^3+^ detection and cellular bioimaging. Nanotechnology.

[119] Lu W, Gong X, Nan M, Liu Y, Shuang S, Dong C (2015). Comparative study for N and S doped carbon dots: Synthesis, characterization and applications for Fe^3+^ probe and cellular imaging. Anal. Chim. Acta.

[120] Iqbal A, Tian Y, Wang X, Gong D, Guo Y, Iqbal K, Wang Z, Liu W, Qin W (2016). Carbon dots prepared by solid state method via citric acid and 1, 10-phenanthroline for selective and sensing detection of Fe^2+^ and Fe^3+^. Sensor. Actuat. B-Chem..

[121] Song Y, Zhu C, Song J, Li H, Du D, Lin Y (2017). Drug-derived bright and color-tunable N-doped carbon dots for cell imaging and sensitive detection of Fe^3+^ in living cells. ACS Appl. Mater. Interfaces..

[122] Yang R, Guo X, Jia L, Zhang Y, Zhao Z, Lonshakov F (2017). Green preparation of carbon dots with mangosteen pulp for the selective detection of Fe^3+^ ions and cell imaging. Appl. Surf. Sci..

[123] Ge L, Yu H, Ren H, Shi B, Guo Q, Gao W, Li Z, Li J (2017). Photoluminescence of carbon dots and their applications in Hela cell imaging and Fe^3+^ ion detection. J. Mater. Sci..

[124] Kim K, Kim J (2018). Synthesis of carbon quantum dots from jujubes for detection of iron(III) ions. J. Nanosci. Nanotechnol..

[125] Shen J, Shang S, Chen X, Wang D, Cai Y (2017). Facile synthesis of fluorescence carbon dots from sweet potato for Fe^3+^ sensing and cell imaging. Mater. Sci. Eng., C.

[126] Aslandaş AM, Balcı N, Arık M, Şakiroğlu H, Onganer Y, Meral K (2015). Liquid nitrogen-assisted synthesis of fluorescent carbon dots from Blueberry and their performance in Fe^3+^ detection. Appl. Surf. Sci..

[127] Sun C, Zhang Y, Wang P, Yang Y, Wang Y, Xu J, Wang Y, William WY (2016). Synthesis of nitrogen and sulfur co-doped carbon dots from garlic for selective detection of Fe^3+^. Nanoscale Res. Lett..

[128] Wen X, Shi L, Wen G, Li Y, Dong C, Yang J, Shuang S (2015). Green synthesis of carbon nanodots from cotton for multicolor imaging, patterning, and sensing. Sensor. Actuat. B-Chem..

[129] Bandi R, Gangapuram BR, Dadigala R, Eslavath R, Singh SS, Guttena V (2016). Facile and green synthesis of fluorescent carbon dots from onion waste and their potential applications as sensor and multicolour imaging agents. RSC Adv..

[130] Tyagi A, Tripathi KM, Singh N, Choudhary S, Gupta RK (2016). Green synthesis of carbon quantum dots from lemon peel waste: applications in sensing and photocatalysis. RSC Adv..

[131] Atchudan R, Edison TNJI, Chakradhar D, Perumal S, Shim J-J, Lee YR (2017). Facile green synthesis of nitrogen-doped carbon dots using *Chionanthus retusus* fruit extract and investigation of their suitability for metal ion sensing and biological applications. Sensor. Actuat. B-Chem..

[132] Wang C, Jiang K, Xu Z, Lin H, Zhang C (2016). Glutathione modified carbon-dots: from aggregation-induced emission enhancement properties to a turn-on sensing of temperature/Fe^3+^ ions in cells. Inorg. Chem. Front..

[133] Deng M, Wang S, Liang C, Shang H, Jiang S (2016). A FRET fluorescent nanosensor based on carbon dots for ratiometric detection of Fe^3+^ in aqueous solution. RSC Adv..

[134] Song L, Cui Y, Zhang C, Hu Z, Liu X (2016). Microwave-assisted facile synthesis of yellow fluorescent carbon dots from o-phenylenediamine for cell imaging and sensitive detection of Fe^3+^ and H_2_O_2_. RSC Adv..

[135] Brewer GJ (2009). Risks of copper and iron toxicity during aging in humans. Chem. Res. Toxicol..

[136] Brewer GJ (2008). The risks of free copper in the body and the development of useful anticopper drugs. Curr. Opin. Clin. Nutr..

[137] Gaggelli E, Kozlowski H, Valensin D, Valensin G (2006). Copper homeostasis and neurodegenerative disorders (Alzheimer’s, prion, and Parkinson’s diseases and amyotrophic lateral sclerosis). Chem. Rev..

[138] Liu Q, Zhang N, Shi H, Ji W, Yuan W, Hu Q (2018). One-step microwave synthesis of carbon dots for highly sensitive and selective detection of copper ion in aqueous solution. New J. Chem..

[139] Chen D, Xu M, Wu W, Li S (2017). Multi-color fluorescent carbon dots for wavelength-selective and ultrasensitive Cu^2+^ sensing. J. Alloys Compd..

[140] Gedda G, Lee C-Y, Lin Y-C, Wu H-F (2016). Green synthesis of carbon dots from prawn shells for highly selective and sensitive detection of copper ions. Sensor. Actuat. B-Chem..

[141] Liu L, Gong H, Li D, Zhao L (2018). Synthesis of carbon dots from pear juice for fluorescence detection of Cu^2+^ ion in water. J. Nanosci. Nanotechnology.

[142] Kumari A, Kumar A, Sahu SK, Kumar S (2018). Synthesis of green fluorescent carbon quantum dots using waste polyolefins residue for Cu^2+^ ion sensing and live cell imaging. Sensor. Actuat. B-Chem..

[143] Liu Y, Zhao Y, Zhang Y (2014). One-step green synthesized fluorescent carbon nanodots from bamboo leaves for copper(II) ion detection. Sensor. Actuat. B-Chem..

[144] Fu Z, Cui F (2016). Thiosemicarbazide chemical functionalized carbon dots as a fluorescent nanosensor for sensing Cu^2+^ and intracellular imaging. RSC Adv..

[145] Vedamalai M, Periasamy AP, Wang C-W, Tseng Y-T, Ho L-C, Shih C-C, Chang H-T (2014). Carbon nanodots prepared from o-phenylenediamine for sensing of Cu^2+^ ions in cells. Nanoscale.

[146] Rao H, Liu W, Lu Z, Wang Y, Ge H, Zou P, Wang X, He H, Zeng X, Wang Y (2016). Silica-coated carbon dots conjugated to CdTe quantum dots: a ratiometric fluorescent probe for copper(II). Microchim. Acta.

[147] Liu C, Ning D, Zhang C, Liu Z, Zhang R, Zhao J, Zhao T, Liu B, Zhang Z (2017). Dual-colored carbon dot ratiometric fluorescent test paper based on a specific spectral energy transfer for semiquantitative assay of copper ions. ACS Appl. Mater. Interfaces..

[148] Zhao A, Zhao C, Li M, Ren J, Qu X (2014). Ionic liquids as precursors for highly luminescent, surface-different nitrogen-doped carbon dots used for label-free detection of Cu^2+^/Fe^3+^ and cell imaging. Anal. Chim. Acta.

[149] Liu J-M, Lin L-P, Wang X-X, Lin S-Q, Cai W-L, Zhang L-H, Zheng Z-Y (2012). Highly selective and sensitive detection of Cu^2+^ with lysine enhancing bovine serum albumin modified-carbon dots fluorescent probe. Analyst.

[150] Gao B, Zhao F, Miao Y, Min H, Xu L, Huang C (2017). Boron-and nitrogen-doped photoluminescent polymer carbon nanoparticles as nanosensors for imaging detection of Cu^2+^ and biothiols in living cells. RSC Adv..

[151] Gogoi N, Barooah M, Majumdar G, Chowdhury D (2015). Carbon dots rooted agarose hydrogel hybrid platform for optical detection and separation of heavy metal ions. ACS Appl. Mater. Interfaces..

[152] Rong M-C, Zhang K-X, Wang Y-R, Chen X (2017). The synthesis of B, N-carbon dots by a combustion method and the application of fluorescence detection for Cu^2+^. Chinese Chem. Lett..

[153] Wang Y, Wu W-T, Wu M-B, Xie H, Hu C, Wu X-Y, Qiu J-S (2015). Yellow-visual fluorescent carbon quantum dots from petroleum coke for the efficient detection of Cu^2+^ ions. New Carbon Mater..

[154] Niu X, Liu G, Li L, Fu Z, Xu H, Cui F (2015). Green and economical synthesis of nitrogen-doped carbon dots from vegetables for sensing and imaging applications. RSC Adv..

[155] Harada M (1995). Minamata disease: methylmercury poisoning in Japan caused by environmental pollution. Crit. Rev. Toxicol..

[156] Harada M (1978). Congenital Minamata disease: intrauterine methylmercury poisoning. Teratology.

[157] Tang W, Wang Y, Wang P, Di J, Yang J, Wu Y (2016). Synthesis of strongly fluorescent carbon quantum dots modified with polyamidoamine and a triethoxysilane as quenchable fluorescent probes for mercury(II). Microchim. Acta.

[158] Ye Q, Yan F, Luo Y, Wang Y, Zhou X, Chen L (2017). Formation of N, S-codoped fluorescent carbon dots from biomass and their application for the selective detection of mercury and iron ion. Spectrochim. Acta A.

[159] Yuan YH, Li RS, Wang Q, Wu ZL, Wang J, Liu H, Huang CZ (2015). Germanium-doped carbon dots as a new type of fluorescent probe for visualizing the dynamic invasions of mercury(II) ions into cancer cells. Nanoscale.

[160] Zhang M, Wang W, Yuan P, Chi C, Zhang J, Zhou N (2017). Synthesis of lanthanum doped carbon dots for detection of mercury ion, multi-color imaging of cells and tissue, and bacteriostasis. Chem. Eng. J..

[161] Li L, Yu B, You T (2015). Nitrogen and sulfur co-doped carbon dots for highly selective and sensitive detection of Hg(II) ions. Biosens. Bioelectron..

[162] Li Y, Zhang Z-Y, Yang H-F, Shao G, Gan F (2018). Highly selective fluorescent carbon dots probe for mercury(II) based on thymine–mercury(II)–thymine structure. RSC Adv..

[163] Xu H, Zhang K, Liu Q, Liu Y, Xie M (2017). Visual and fluorescent detection of mercury ions by using a dually emissive ratiometric nanohybrid containing carbon dots and CdTe quantum dots. Microchim. Acta.

[164] Liu W, Wang X, Wang Y, Li J, Shen D, Kang Q, Chen L (2018). Ratiometric fluorescence sensor based on dithiothreitol modified carbon dots-gold nanoclusters for the sensitive detection of mercury ions in water samples. Sensor. Actuat. B-Chem..

[165] Zhao J, Huang M, Zhang L, Zou M, Chen D, Huang Y, Zhao S (2017). Unique approach to develop carbon dot-based nanohybrid near-infrared ratiometric fluorescent sensor for the detection of mercury ions. Anal. Chem..

[166] Wang Y, Kim S-H, Feng L (2015). Highly luminescent N, S–Co-doped carbon dots and their direct use as mercury(II) sensor. Anal. Chim. Acta.

[167] Tabaraki R, Sadeghinejad N (2018). Microwave assisted synthesis of doped carbon dots and their application as green and simple turn off–on fluorescent sensor for mercury(II) and iodide in environmental samples. Ecotox. Environ. Safe..

[168] Gupta A, Chaudhary A, Mehta P, Dwivedi C, Khan S, Verma NC, Nandi CK (2015). Nitrogen-doped, thiol-functionalized carbon dots for ultrasensitive Hg(II) detection. Chem. Commun..

[169] Zhao F, Qian J, Quan F, Wu C, Zheng Y, Zhou L (2017). Aconitic acid derived carbon dots as recyclable on–off–on fluorescent nanoprobes for sensitive detection of mercury(II) ions, cysteine and cellular imaging. RSC Adv..

[170] Yan F, Shi D, Zheng T, Yun K, Zhou X, Chen L (2016). Carbon dots as nanosensor for sensitive and selective detection of Hg^2+^ and l-cysteine by means of fluorescence off–on switching. Sensor. Actuat. B-Chem..

[171] He J, Zhang H, Zou J, Liu Y, Zhuang J, Xiao Y, Lei B (2016). Carbon dots-based fluorescent probe for off-on sensing of Hg(II) and I^−^. Biosens. Bioelectron..

[172] Huang H, Weng Y, Zheng L, Yao B, Weng W, Lin X (2017). Nitrogen-doped carbon quantum dots as fluorescent probe for off-on detection of mercury ions, l-cysteine and iodide ions. J. Colloid Interf. Sci..

[173] Ma Y, Zhang Z, Xu Y, Ma M, Chen B, Wei L, Xiao L (2016). A bright carbon-dot-based fluorescent probe for selective and sensitive detection of mercury ions. Talanta.

[174] Lu S, Wu D, Li G, Lv Z, Chen Z, Chen L, Chen G, Xia L, You J, Wu Y (2016). Carbon dots-based ratiometric nanosensor for highly sensitive and selective detection of mercury(II) ions and glutathione. RSC Adv..

[175] Liang Y, Zhang H, Zhang Y, Chen F (2015). Simple hydrothermal preparation of carbon nanodots and their application in colorimetric and fluorimetric detection of mercury ions. Anal. Method..

[176] Wang L, Li B, Xu F, Shi X, Feng D, Wei D, Li Y, Feng Y, Wang Y, Jia D (2016). High-yield synthesis of strong photoluminescent N-doped carbon nanodots derived from hydrosoluble chitosan for mercury ion sensing via smartphone APP. Biosens. Bioelectron..

[177] Zhang Y, Cui P, Zhang F, Feng X, Wang Y, Yang Y, Liu X (2016). Fluorescent probes for off–on highly sensitive detection of Hg^2+^ and l-cysteine based on nitrogen-doped carbon dots. Talanta.

[178] Ahmed KBA, Kumar S, Veerappan A (2016). A facile method to prepare fluorescent carbon dots and their application in selective colorimetric sensing of silver ion through the formation of silver nanoparticles. J. Lumin..

[179] Gao X, Lu Y, Zhang R, He S, Ju J, Liu M, Li L, Chen W (2015). One-pot synthesis of carbon nanodots for fluorescence turn-on detection of Ag^+^ based on the Ag^+^-induced enhancement of fluorescence. J. Mater. Chem. C.

[180] Vaz R, Bettini J, Júnior JGF, Lima EDS, Botero WG, Santos JCC, Schiavon MA (2017). High luminescent carbon dots as an eco-friendly fluorescence sensor for Cr(VI) determination in water and soil samples. J. Photoch. Photobio. A.

[181] Shen J, Shang S, Chen X, Wang D, Cai Y (2017). Highly fluorescent N, S-co-doped carbon dots and their potential applications as antioxidants and sensitive probes for Cr(VI) detection. Sensor. Actuat. B-Chem..

[182] Liu Y, Hu J, Li Y, Wei H-P, Li X-S, Zhang X-H, Chen S-M, Chen X-Q (2015). Synthesis of polyethyleneimine capped carbon dots for preconcentration and slurry sampling analysis of trace chromium in environmental water samples. Talanta.

[183] Chen J, Li Y, Lv K, Zhong W, Wang H, Wu Z, Yi P, Jiang J (2016). Cyclam-functionalized carbon dots sensor for sensitive and selective detection of copper(II) ion and sulfide anion in aqueous media and its imaging in live cells. Sensor. Actuat. B-Chem..

[184] Amin N, Afkhami A, Madrakian T (2018). Construction of a novel off-on fluorescence sensor for highly selective sensing of selenite based on europium ions induced crosslinking of nitrogen-doped carbon dots. J. Lumin..

[185] Baruah U, Gogoi N, Majumdar G, Chowdhury D (2015). β-Cyclodextrin and calix [4] arene-25, 26, 27, 28-tetrol capped carbon dots for selective and sensitive detection of fluoride. Carbohyd. Polym..

[186] Ganguly P, Alam SF (2015). Role of homocysteine in the development of cardiovascular disease. Nutr. J..

[187] Yang X, Guo Y, Strongin RM (2011). Conjugate addition/cyclization sequence enables selective and simultaneous fluorescence detection of cysteine and homocysteine. Angew. Chem. Int.-Ed..

[188] Barford D (2004). The role of cysteine residues as redox-sensitive regulatory switches. Curr. Opin. Struc. Biol..

[189] Choi J, Liu R-M, Kundu RK, Sangiorgi F, Wu W, Maxson R, Forman HJ (2000). Molecular mechanism of decreased glutathione content in human immunodeficiency virus type 1 Tat-transgenic mice. J. Biol. Chem..

[190] Staal FJ, Ela SW, Roederer M, Anderson M, Herzenberg L (1992). Glutathione deficiency and human immunodeficiency virus infection. The Lancet..

[191] Song Z, Quan F, Xu Y, Liu M, Cui L, Liu J (2016). Multifunctional N, S co-doped carbon quantum dots with pH-and thermo-dependent switchable fluorescent properties and highly selective detection of glutathione. Carbon.

[192] Wang Y, Jiang K, Zhu J, Zhang L, Lin H (2015). A FRET-based carbon dot–MnO_2_ nanosheet architecture for glutathione sensing in human whole blood samples. Chem. Commun..

[193] Wu D, Li G, Chen X, Qiu N, Shi X, Chen G, Sun Z, You J, Wu Y (2017). Fluorometric determination and imaging of glutathione based on a thiol-triggered inner filter effect on the fluorescence of carbon dots. Microchim. Acta.

[194] Deng J, Lu Q, Hou Y, Liu M, Li H, Zhang Y, Yao S (2015). Nanosensor composed of nitrogen-doped carbon dots and gold nanoparticles for highly selective detection of cysteine with multiple signals. Anal. Chem..

[195] Brookes PS, Yoon Y, Robotham JL, Anders M, Sheu S-S (2004). Calcium, ATP, and ROS: a mitochondrial love-hate triangle. Am. J. Physiol.-Cell Physiol..

[196] O’Reilly CM, Fogarty KE, Drummond RM, Tuft RA, Walsh JV (2003). Quantitative analysis of spontaneous mitochondrial depolarizations. Biophys. J..

[197] Gong Y, Yu B, Yang W, Zhang X (2016). Phosphorus, and nitrogen co-doped carbon dots as a fluorescent probe for real-time measurement of reactive oxygen and nitrogen species inside macrophages. Biosens. Bioelectron..

[198] Simões EF, da Silva JCE, Leitão JM (2015). Peroxynitrite and nitric oxide fluorescence sensing by ethylenediamine doped carbon dots. Sensor. Actuat. B-Chem..

[199] Wu J, Wang X, Lin Y, Zheng Y, Lin J-M (2016). Peroxynitrous-acid-induced chemiluminescence detection of nitrite based on microfluidic chip. Talanta.

[200] Stone JR, Yang S (2006). Hydrogen peroxide: a signaling messenger. Antioxid. Redox Sign..

[201] Su Y, Zhou X, Long Y, Li W (2018). Immobilization of horseradish peroxidase on amino-functionalized carbon dots for the sensitive detection of hydrogen peroxide. Microchim. Acta.

[202] Huang S, Wang L, Zhu F, Su W, Sheng J, Huang C, Xiao Q (2015). A ratiometric nanosensor based on fluorescent carbon dots for label-free and highly selective recognition of DNA. RSC Adv..

[203] Loo AH, Sofer Z, Bouša D, Ulbrich P, Bonanni A, Pumera M (2016). Carboxylic carbon quantum dots as a fluorescent sensing platform for DNA detection. ACS Appl. Mater. Interfaces..

[204] Guo R, Chen B, Li F, Weng S, Zheng Z, Chen M, Wu W, Lin X, Yang C (2018). Positive carbon dots with dual roles of nanoquencher and reference signal for the ratiometric fluorescence sensing of DNA. Sensor. Actuat. B-Chem..

[205] Pang S, Zhang Y, Wu C, Feng S (2016). Fluorescent carbon dots sensor for highly sensitive detection of guanine. Sensor. Actuat. B-Chem..

[206] Li L, Lu Y, Ding Y, Zhang F, Wang Y (2011). Facile aqueous synthesis of functionalized CdTe nanoparticles and their application as fluorescence probes for determination of adenine and guanine. Can. J. Chem..

[207] Huang S, Wang L, Huang C, Xie J, Su W, Sheng J, Xiao Q (2015). A carbon dots based fluorescent probe for selective and sensitive detection of hemoglobin. Sensor. Actuat. B-Chem..

[208] Barati A, Shamsipur M, Abdollahi H (2015). Hemoglobin detection using carbon dots as a fluorescence probe. Biosens. Bioelectron..

[209] Enserink M (2001). This time it was real: knowledge of anthrax put to the test. Science.

[210] Chen H, Xie Y, Kirillov AM, Liu L, Yu M, Liu W, Tang Y (2015). A ratiometric fluorescent nanoprobe based on terbium functionalized carbon dots for highly sensitive detection of an anthrax biomarker. Chem. Commun..

[211] Rong M, Liang Y, Zhao D, Chen B, Pan C, Deng X, Chen Y, He J (2018). A ratiometric fluorescence visual test paper for an anthrax biomarker based on functionalized manganese-doped carbon dots. Sensor. Actuat. B-Chem..

[212] McCoy CP, Stomeo F, Plush SE, Gunnlaugsson T (2006). Soft matter ph sensing: from luminescent lanthanide ph switches in solution to sensing in hydrogels. Chem. Mater..

[213] Qu K, Wang J, Ren J, Qu X (2013). Carbon dots prepared by hydrothermal treatment of dopamine as an effective fluorescent sensing platform for the label-free detection of iron(III) ions and dopamine. Chem.-Eur. J..

[214] Shen P, Xia Y (2014). Synthesis-modification integration: one-step fabrication of boronic acid functionalized carbon dots for fluorescent blood sugar sensing. Anal. Chem..

[215] Wang H, Yi J, Velado D, Yu Y, Zhou S (2015). Immobilization of carbon dots in molecularly imprinted microgels for optical sensing of glucose at physiological pH. ACS Appl. Mater. Interfaces..

[216] Miao H, Wang L, Zhuo Y, Zhou Z, Yang X (2016). Label-free fluorimetric detection of CEA using carbon dots derived from tomato juice. Biosens. Bioelectron..

[217] Motaghi H, Mehrgardi MA, Bouvet P (2017). Carbon dots-AS1411 aptamer nanoconjugate for ultrasensitive spectrofluorometric detection of cancer cells. Sci. Rep..

[218] Qian ZS, Chai LJ, Huang YY, Tang C, Jia Shen J, Chen JR, Feng H (2015). A real-time fluorescent assay for the detection of alkaline phosphatase activity based on carbon quantum dots. Biosens. Bioelectron..

[219] Liu S, Zhao N, Cheng Z, Liu H (2015). Amino-functionalized green fluorescent carbon dots as surface energy transfer biosensors for hyaluronidase. Nanoscale.

[220] Amjadi M, Hallaj T, Kouhi Z (2018). An enzyme-free fluorescent probe based on carbon dots: MnO_2_ nanosheets for determination of uric acid. J. Photoch. Photobio. A.

[221] Zhao D, Chen C, Sun J, Yang X (2016). Carbon dots-assisted colorimetric and fluorometric dual-mode protocol for acetylcholinesterase activity and inhibitors screening based on the inner filter effect of silver nanoparticles. Analyst.

[222] Korten A, Lodder J, Vreeling F, Boreas A, van Raak L, Kessels F (2001). Stroke and idiopathic Parkinson’s disease: does a shortage of dopamine offer protection against stroke?. Movement Disord..

[223] Elsworth JD, Roth RH (1997). Dopamine synthesis, uptake, metabolism, and receptors: relevance to gene therapy of Parkinson’s disease. Exp. Neurol..

[224] Casalini S, Leonardi F, Cramer T, Biscarini F (2013). Organic field-effect transistor for label-free dopamine sensing. Org. Electron..

[225] Li H, Liu J, Yang M, Kong W, Huang H, Liu Y (2014). Highly sensitive, stable, and precise detection of dopamine with carbon dots/tyrosinase hybrid as fluorescent probe. RSC Adv..

[226] Li H, Kong W, Liu J, Liu N, Huang H, Liu Y, Kang Z (2015). Fluorescent N-doped carbon dots for both cellular imaging and highly-sensitive catechol detection. Carbon.

[227] Wang N, Wang Y, Guo T, Yang T, Chen M, Wang J (2016). Green preparation of carbon dots with papaya as carbon source for effective fluorescent sensing of iron(III) and *Escherichia coli*. Biosens. Bioelectron..

[228] Lai IP-J, Harroun SG, Chen S-Y, Unnikrishnan B, Li Y-J, Huang C-C (2016). Solid-state synthesis of self-functional carbon quantum dots for detection of bacteria and tumor cells. Sensor. Actuat. B-Chem..

[229] Duan N, Wu S, Dai S, Miao T, Chen J, Wang Z (2015). Simultaneous detection of pathogenic bacteria using an aptamer based biosensor and dual fluorescence resonance energy transfer from quantum dots to carbon nanoparticles. Microchim. Acta.

[230] Yang L, Deng W, Cheng C, Tan Y, Xie Q, Yao S (2018). Fluorescent immunoassay for the detection of pathogenic bacteria at the single-cell level using carbon dots-encapsulated breakable organosilica nanocapsule as labels. ACS Appl. Mater. Interfaces..

[231] Zhang J, Zhao X, Xian M, Dong C, Shuang S (2018). Folic acid-conjugated green luminescent carbon dots as a nanoprobe for identifying folate receptor-positive cancer cells. Talanta.

[232] Gao G, Jiang Y-W, Yang J, Wu F-G (2017). Mitochondria-targetable carbon quantum dots for differentiating cancerous cells from normal cells. Nanoscale.

[233] Chen BB, Liu ZX, Zou HY, Huang CZ (2016). Highly selective detection of 2,4,6-trinitrophenol by using newly developed terbium-doped blue carbon dots. Analyst.

[234] Cheng F, An X, Zheng C, Cao S (2015). Green synthesis of fluorescent hydrophobic carbon quantum dots and their use for 2,4,6-trinitrophenol detection. RSC Adv..

[235] Ju B, Wang Y, Zhang Y-M, Zhang T, Liu Z, Li M, Zhang SX-A (2018). Photo-stable and low-toxic yellow-green carbon dots for highly selective detection of explosive 2,4,6-trinitrophenol based on dual electron transfer mechanism. ACS Appl. Mater. Interfaces..

[236] Shi D, Yan F, Zheng T, Wang Y, Zhou X, Chen L (2015). P-doped carbon dots act as a nanosensor for trace 2,4,6-trinitrophenol detection and a fluorescent reagent for biological imaging. RSC Adv..

[237] Zhang L, Han Y, Zhu J, Zhai Y, Dong S (2015). Simple and sensitive fluorescent and electrochemical trinitrotoluene sensors based on aqueous carbon dots. Anal. Chem..

[238] Zou S, Hou C, Fa H, Zhang L, Ma Y, Dong L, Li D, Huo D, Yang M (2017). An efficient fluorescent probe for fluazinam using N, S co-doped carbon dots from l-cysteine. Sensor. Actuat. B-Chem..

[239] Xu H, Yang X, Li G, Zhao C, Liao X (2015). Green synthesis of fluorescent carbon dots for selective detection of tartrazine in food samples. J. Agr. Food Chem..

[240] Wang B, Chen Y, Wu Y, Weng B, Liu Y, Lu Z, Li CM, Yu C (2016). Aptamer induced assembly of fluorescent nitrogen-doped carbon dots on gold nanoparticles for sensitive detection of AFB1. Biosens. Bioelectron..

[241] Hou J, Li H, Wang L, Zhang P, Zhou T, Ding H, Ding L (2016). Rapid microwave-assisted synthesis of molecularly imprinted polymers on carbon quantum dots for fluorescent sensing of tetracycline in milk. Talanta.

[242] Feng L, Tan L, Li H, Xu Z, Shen G, Tang Y (2015). Selective fluorescent sensing of α-amanitin in serum using carbon quantum dots-embedded specificity determinant imprinted polymers. Biosens. Bioelectron..

[243] Hou J, Dong J, Zhu H, Teng X, Ai S, Mang M (2015). A simple and sensitive fluorescent sensor for methyl parathion based on l-tyrosine methyl ester functionalized carbon dots. Biosens. Bioelectron..

[244] Hou J, Tian Z, Xie H, Tian Q, Ai S (2016). A fluorescence resonance energy transfer sensor based on quaternized carbon dots and Ellman’s test for ultrasensitive detection of dichlorvos. Sensor. Actuat. B-Chem..

[245] Zheng M, Wang C, Wang Y, Wei W, Ma S, Sun X, He J (2018). Green synthesis of carbon dots functionalized silver nanoparticles for the colorimetric detection of phoxim. Talanta.

[246] Mazhari BBZ, Agsar D (2016). Detection of phenols from industrial effluents using streptomyces mediated gold nanoparticles. Indian J. Mater. Sci..

[247] Ahmed GHG, Laí-o RB, Calzón JAG, García MED (2015). Highly fluorescent carbon dots as nanoprobes for sensitive and selective determination of 4-nitrophenol in surface waters. Microchim. Acta.

[248] Hao T, Wei X, Nie Y, Xu Y, Yan Y, Zhou Z (2016). An eco-friendly molecularly imprinted fluorescence composite material based on carbon dots for fluorescent detection of 4-nitrophenol. Microchim. Acta.

[249] Xiao N, Liu SG, Mo S, Li N, Ju YJ, Ling Y, Li NB, Luo HQ (2018). Highly selective detection of p-nitrophenol using fluorescence assay based on boron, nitrogen co-doped carbon dots. Talanta.

[250] Ni P, Dai H, Li Z, Sun Y, Hu J, Jiang S, Wang Y, Li Z (2015). Carbon dots based fluorescent sensor for sensitive determination of hydroquinone. Talanta.

[251] Liu G, Chen Z, Jiang X, Feng D-Q, Zhao J, Fan D, Wang W (2016). In-situ hydrothermal synthesis of molecularly imprinted polymers coated carbon dots for fluorescent detection of bisphenol A. Sensor. Actuat. B-Chem..

[252] Xue M, Zou M, Zhao J, Zhan Z, Zhao S (2015). Green preparation of fluorescent carbon dots from lychee seeds and their application for the selective detection of methylene blue and imaging in living cells. J. Mater. Chem. B.

[253] Zhao D, Chen C, Lu L, Yang F, Yang X (2015). A dual-mode colorimetric and fluorometric light on sensor for thiocyanate based on fluorescent carbon dots and unmodified gold nanoparticles. Analyst.

[254] Luo X, Zhang W, Han Y, Chen X, Zhu L, Tang W, Wang J, Yue T, Li Z (2018). N, S co-doped carbon dots based fluorescent on-off-on sensor for determination of ascorbic acid in common fruits. Food Chem..

[255] Liu J, Chen Y, Wang W, Feng J, Liang M, Ma S, Chen X (2015). Switch-on fluorescent sensing of ascorbic acid in food samples based on carbon quantum dots–MnO_2_ probe. J. Agr. Food Chem..

[256] Ding L, Yang H, Ge S, Yu J (2018). Fluorescent carbon dots nanosensor for label-free determination of vitamin B 12 based on inner filter effect. Spectrochim. Acta A.

[257] Zeng H, Li L, Ding Y, Zhuang Q (2018). Simple and selective determination of 6-thioguanine by using polyethylenimine (PEI) functionalized carbon dots. Talanta.

[258] Garg D, Mehta A, Mishra A, Basu S (2018). A sensitive turn on fluorescent probe for detection of biothiols using MnO_2_@ carbon dots nanocomposites. Spectrochim. Acta A.

[259] Qu F, Sun Z, Liu D, Zhao X, You J (2016). Direct and indirect fluorescent detection of tetracyclines using dually emitting carbon dots. Microchim. Acta.

[260] Fu X, Lv R, Su J, Li H, Yang B, Gu W, Liu X (2018). A dual-emission nano-rod MOF equipped with carbon dots for visual detection of doxycycline and sensitive sensing of MnO^4−^. RSC Adv..

[261] Wang Y, Ma T, Ma S, Liu Y, Tian Y, Wang R, Jiang Y, Hou D, Wang J (2017). Fluorometric determination of the antibiotic kanamycin by aptamer-induced FRET quenching and recovery between MoS_2_ nanosheets and carbon dots. Microchim. Acta.

[262] Zou Y, Yan F, Zheng T, Shi D, Sun F, Yang N, Chen L (2015). Highly luminescent organosilane-functionalized carbon dots as a nanosensor for sensitive and selective detection of quercetin in aqueous solution. Talanta.

[263] Zhang Y, Gao Z, Zhang W, Wang W, Chang J, Kai J (2018). Fluorescent carbon dots as nanoprobe for determination of lidocaine hydrochloride. Sensor. Actuat. B-Chem..

[264] Eatemadi A, Daraee H, Karimkhanloo H, Kouhi M, Zarghami N, Akbarzadeh A, Abasi M, Hanifehpour Y, Joo SW (2014). Carbon nanotubes: properties, synthesis, purification, and medical applications. Nanoscale Res. Lett..

[265] Zhu Y, Murali S, Cai W, Li X, Suk JW, Potts JR, Ruoff RS (2010). Graphene and graphene oxide: synthesis, properties, and applications. Adv. Mater..

[266] Sun S, Zhang L, Jiang K, Wu A, Lin H (2016). Toward high-efficient red emissive carbon dots: facile preparation, unique properties, and applications as multifunctional theranostic agents. Chem. Mater..

[267] Martinac B (2017). Single-molecule FRET studies of ion channels. Prog. Biophys. Mol. Biol..

